# A Mass Spectrometry Database for Sea Cucumber Triterpene Glycosides

**DOI:** 10.3390/metabo13070783

**Published:** 2023-06-23

**Authors:** Roman S. Popov, Natalia V. Ivanchina, Alexandra S. Silchenko, Sergey A. Avilov, Vladimir I. Kalinin, Timofey V. Malyarenko, Valentin A. Stonik, Pavel S. Dmitrenok

**Affiliations:** G.B. Elyakov Pacific Institute of Bioorganic Chemistry, Far Eastern Branch of Russian Academy of Sciences, 159 Prospect 100-let Vladivostoku, Vladivostok 690022, Russia; ivanchina@piboc.dvo.ru (N.V.I.); silchenko_als@piboc.dvo.ru (A.S.S.); avilov_sa@piboc.dvo.ru (S.A.A.); kalininv@piboc.dvo.ru (V.I.K.); malyarenko-tv@mail.ru (T.V.M.); stonik@piboc.dvo.ru (V.A.S.)

**Keywords:** mass spectrometry database, sea cucumber, triterpene glycosides, metabolite annotation and identification, mass spectrometry, ultraperformance liquid chromatography, *Eupentacta fraudatrix*

## Abstract

Sea cucumber triterpene glycosides are a class of secondary metabolites that possess distinctive chemical structures and exhibit a variety of biological and pharmacological activities. The application of MS-based approaches for the study of triterpene glycosides allows rapid evaluation of the structural diversity of metabolites in complex mixtures. However, the identification of the detected triterpene glycosides can be challenging. The objective of this study is to establish the first spectral library containing the mass spectra of sea cucumber triterpene glycosides using ultraperformance liquid chromatography-quadrupole time-of-flight mass spectrometry. The library contains the electrospray ionization tandem mass spectra and retention times of 191 triterpene glycosides previously isolated from 15 sea cucumber species and one starfish at the Laboratory of the Chemistry of Marine Natural Products of the G.B. Elyakov Pacific Institute of Bioorganic Chemistry. In addition, the chromatographic behavior and some structure-related neutral losses in tandem MS are discussed. The obtained data will accelerate the accurate dereplication of known triterpene glycosides and the annotation of novel compounds, as we demonstrated by the processing of LC-MS/MS data of *Eupentacta fraudatrix* extract.

## 1. Introduction

**Figure 1:**
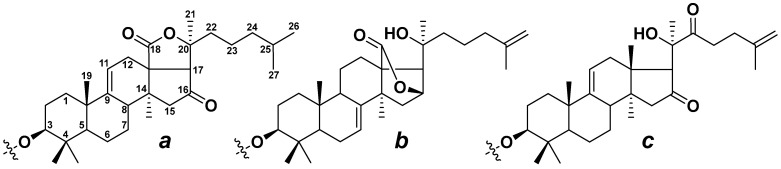
Structures of lanostane-type aglycons occurring in sea cucumber triterpene glycosides with 18(20)-lactone (**a**), 18(16)-lactone (**b**), and without a lactone cycle (**c**).

Sea cucumbers are a source of a wide range of secondary metabolites, including triterpene glycosides, which are of particular interest due to their diverse biological and pharmacological effects, including cytotoxic, hemolytic, antiviral, antifungal, and immunomodulatory properties [1,2,3,4,5]. The sea cucumber triterpene glycosides are characterized by unique chemical structures with a conservative structural framework but significant natural diversity in some features, leading to a vast potential for structural variability. Triterpene glycosides have lanostane- and norlanostane-type aglycons, and most of them contain an 18(20)-lactone, although aglycons with an 18(16)-lactone or without a lactone cycle are not uncommon (Figure 1). The polycyclic nuclei of the aglycons preferably contain a 7(8)- or 9(11)-double bond and may have oxygen-containing substituents at C-12, C-17, or C-16. The side chains are a major source of aglycon structural diversity due to chain length reduction and modification by oxygen-containing substituents and double bonds. The carbohydrate moiety of triterpene glycosides is attached to C-3 of the aglycon and can contain up to six monosaccharide units, with glucose (Glc), quinovose (Qui), xylose (Xyl), 3-*O*-methylglucose (MeGlc), and 3-*O*-methylxylose (MeXyl) being the most common monosaccharides. Xylose is always the first unit of the oligosaccharide chain. Quinovose and glucose are often the second and third monosaccharides, correspondingly, and methylated monosaccharides are always terminal. The oligosaccharide chains of many triterpene glycosides may contain up to four sulfate groups. Short oligosaccharide chains having three or four units are usually considered linear, while longer chains are branched at the first or second monosaccharide unit [6,7,8,9,10].

Due to the extreme complexity of the sea cucumber extracts and the enormous diversity of triterpene glycoside structures, conventional approaches in natural product research, which require the isolation of individual compounds by a combination of chromatographic techniques followed by structure elucidation by nuclear magnetic resonance (NMR) spectroscopy, mass spectrometry (MS), or other analytical techniques, are time- and labor-intensive procedures. The application of MS-based methods, such as liquid chromatography-electrospray mass spectrometry (LC-ESI MS), to the investigation of complex fractions of sea cucumber triterpene glycosides enables the acquisition of qualitative and quantitative information about the chemical composition, which can subsequently be used to facilitate the isolation of biologically active compounds as well as the study of biosynthetic pathways and biological roles of target metabolites. A recent review demonstrated the advantages of MS-based metabolomics as a powerful research tool for assessing the biochemical diversity of sea cucumbers and starfish [11].

The application of MS-based approaches to the study of complex mixtures of natural products can be fraught with difficulties, of which metabolite identification is a current bottleneck [12]. High confidence identification can be achieved by comparing data, including monoisotopic mass, MS/MS spectra, and retention times with data from a reference standard [13], which requires either an extensive chemical library or specific databases. Chemical libraries of natural products are very limited and cover only a small part of the total scope of biochemical diversity, especially in the field of marine natural products, due to the inaccessibility of sources and the enormously labor-intensive processes of synthesis or isolation of individual compounds and the maintenance of chemical collections. Therefore, the availability of comprehensive, open-access databases is extremely important for the successful application of mass spectrometry to the analysis of natural products [12].

The advancement of metabolomics as a powerful research tool in the field of natural products over the last two decades has promoted the development of open-access resources and databases containing experimental data and MS/MS spectra, such as MassBank [14], Metlin [15], MassBank of North America (https://mona.fiehnlab.ucdavis.edu, accessed on 1 April 2023), BMDMS-NP [16], and GNPS [17]. The latter is a web-based mass spectrometry platform that combines research tools for storing, analyzing, annotating, and sharing experimental data with an open-access MS/MS database combining several dozen spectral libraries, including several specialized natural product libraries such as the Lichen DataBase (LDB) [18], the Monoterpene Indole Alkaloids DataBase (MIADB) [19], the IsoQuinoline and Annonaceous Metabolites Data Base (IQAMDB) [20], the phytochemical database Sam Sik Kang Legacy Library [21], and others.

However, despite the increasing coverage of natural products in existing spectral libraries, data on marine natural products remains very limited. To our knowledge, there are no currently open spectral libraries containing MS/MS spectra of sea cucumber triterpene glycosides. This limitation, coupled with the significant structural diversity of these compounds, restricts the broad application of mass spectrometry in the investigation of triterpene glycosides.

Over the past decades, more than 700 new marine natural products, including a large series of sea cucumber triterpene glycosides, have been isolated and structurally elucidated at the G.B. Elyakov Pacific Institute of Bioorganic Chemistry. Herein, we report on the establishment of a sea cucumber triterpene glycoside database comprising MS/MS spectra and retention times for 191 compounds previously isolated from 15 species of Holothuroidea and one starfish at the Laboratory of the Chemistry of Marine Natural Products of the G.B. Elyakov Pacific Institute of Bioorganic Chemistry. The dataset and spectral library are open-access and can be downloaded as Supplementary Material or from MetaboLights using the identifier MTBLS7506.

## 2. Materials and Methods

### 2.1. Chemicals

Acetonitrile (UHPLC grade) was purchased from Panreac (Barcelona, Spain), and methanol (HPLC grade) was purchased from J.T. Baker (Deventer, The Netherlands). Water was obtained from an aquaMAX Ultra 370 Series water purification system (YoungIn Chromass, Anyang, Republic of Korea). MS-grade formic acid was purchased from Merck (Darmstadt, Germany).

The chemical library of triterpene glycosides is maintained in the Laboratory of the Chemistry of Marine Natural Products of the G.B. Elyakov Pacific Institute of Bioorganic Chemistry, FEB RAS. All the triterpene glycosides have previously been isolated as individual compounds from various sea cucumber species by a combination of column chromatography and HPLC, and the structures of these compounds have been elucidated by several independent methods, including NMR spectroscopy and MS. Specimens of sea cucumbers and starfish were collected from various locations, including the South China Sea, the Sea of Okhotsk, the Sea of Japan, the Bering Sea, the Arabian Sea, and the Eastern Weddell Sea, between 1982 and 2019, by scuba diving, Sigsbee trawling, or scallop dredging. Sampling and identification were performed by Prof. Valery S. Levin, Dr. Igor Yu. Dolmatov, Dr. Vadim G. Stepanov, Dr. Alexey V. Smirnov, Dr. Salim Sh. Dautov, and Boris B. Grebnev; voucher specimens are kept in the collection of the G.B. Elyakov Pacific Institute of Bioorganic Chemistry, FEB RAS. Most of the triterpene glycosides were obtained between 2011 and 2022 and stored in the dried state at a low temperature (−20 °C).

### 2.2. Sample Preparation

The dried samples of triterpene glycosides were dissolved in 50% methanol in water (*v*/*v*) (for non-sulfated, mono- and disulfated compounds) or in water (for tri- and tetrasulfated compounds) at a concentration of 0.2 mg/mL. If necessary, certain samples were centrifuged at 10,000× *g* for 5 min. The 0.5 mL solution was transferred to a 2 mL autosampler vial for LC-MS analysis.

### 2.3. Data Acquisition

Data were acquired using a Bruker Elute UHPLC system (Bruker Daltonics, Bremen, Germany) consisting of an Elute Pump HPG 1300, an Elute Autosampler UHPLC, and an Elute Column Oven coupled to a Bruker Impact II Q-TOF mass spectrometer (Bruker Daltonics, Bremen, Germany). Chromatographic separation was performed using an InfinityLab Poroshell 120 SB-C18 column (2.1 × 150 mm, 1.9 μm, Agilent Technologies, Santa Clara, CA, USA) at a flow rate of 0.3 mL/min and a temperature of 40 °C. The mobile phase consisted of water (eluent A) and acetonitrile (eluent B), both acidified with 0.1% formic acid. The gradient program used was as follows: eluent B was increased from 15 to 30% over 3 min, then from 30 to 60% eluent B from 3 to 20 min, from 60 to 100% eluent B from 20 to 21 min, held isocratically at 100% eluent B from 21 to 25 min, and finally reduced from 100 to 15% eluent B over 25 to 25.2 min. After returning to the initial conditions, equilibration was achieved after 2.8 min. The injection volume was 2 μL in the partial loop injection mode.

Mass spectrometry detection was performed using an ESI ionization source in negative ion mode. Optimized ionization parameters for ESI were as follows: a capillary voltage of 4 kV, nebulization with nitrogen at 2.5 bar, and a dry gas flow of 6 L/min at a temperature of 215 °C. Mass spectra were recorded in the *m/z* mass range of 80–2000 at 1.5 Hz. The isCID energy was increased to 90.0 eV to avoid unnecessary adduct and dimer formation.

Collision-induced dissociation (CID) product ion mass spectra were recorded in auto-MS/MS mode. The precursor ions were isolated with an isolation width of 4 Th. The collision energy was optimized to obtain the most informative MS/MS spectra by adjusting it according to the precursor ion charge, utilizing values of 120, 60, 43, and 40 eV for precursor ion charges of 1, 2, 3, and 4, respectively, based on early preliminary experiments. Mass spectrometry acquisitions were split into four scan events, with the first being an MS scan, followed by three MS/MS scans of the precursor ion with the highest intensity detected during the first scan event. The MS/MS spectra were obtained using the MultiCE option, which allowed for acquisition with varying collision energies. During the acquisition cycle, the first MS/MS scan was obtained at 75% of the target collision energy, followed by a second scan at 100%, and a third scan at 120%. Detailed mass spectra acquisition parameters are provided as supplementary material (Table S1). If the substance was unsuitable for reversed-phase LC, the sample was directly injected into the ESI ion source using a syringe pump at a flow rate of 0.1 mL/min.

The mass spectrometer was operated using otofControl (ver. 4.1, Bruker Daltonics, Bremen, Germany). The instrument was calibrated using the ESI-L Low Concentration Tuning Mix (Agilent Technologies, Santa Clara, CA, USA).

### 2.4. Data Processing and Spectral Library Constitution

A set of 191 files in Bruker .d format containing raw data was generated as a result of the analysis of triterpene glycoside reference standards. Raw data were manually inspected, and extracted ion chromatograms were constructed for the corresponding calculated *m/z* of the precursor ions using DataAnalysis software (Ver. 4.4, Bruker Daltonics, Bremen, Germany). The most intense MS and informative MS/MS scans obtained at different collision energies of each compound were added to the spectral library using DataAnalysis and LibraryEditor software (ver. 4.4, Bruker Daltonics, Bremen, Germany). The resulting library in .mlb format contained 191 MS and 494 MS/MS spectra, along with information about the name, structure, molecular formula, retention time, and acquisition parameters, and is available as Supplementary Material.

For the MS spectral library in .mgf format constitution, the LC-MS raw files were converted to an open-source mzML format using the MSConvert utility [22]. The mzML files were then processed using the MZmine 2.53 software [23], which allowed the scans associated with the signal of interest to be exported into separate mzML files. The files containing the most informative MS/MS scans were processed using MZmine to generate a list of masses from the raw MS data. Mass detection was performed using the wavelet transform algorithm with the following parameters: noise level—20; scale level—10; and wavelet window size—100%. The resulting peak lists from each file were exported as separate mgf files, which were then combined into a single mgf file using a custom script. A set of mzML and mgf files containing raw and derived MS and MS/MS spectra at different collision energies, as well as a merged mgf file containing all MS/MS data, are deposited on MetaboLights [24] under the identifier MTBLS7506 and are also available as Supplementary Material.

The mgf file containing the combined MS/MS data was used as input data to create a molecular network using the online molecular networking workflow (version release_28.2) at GNPS with a parent mass tolerance of 0.02 Da and an MS/MS fragment ion tolerance of 0.05 Da. A molecular network was created with edges filtered to have a cosine score above 0.7 and more than 6 matching peaks. Furthermore, edges between two nodes were kept in the network if and only if each node appeared in the top 10 most similar nodes to the other. The maximum size of a molecular family was set to 100, and the lowest-scoring edges were removed from molecular families until the molecular family size was below this threshold. The molecular networks were visualized using Cytoscape software [25]. The molecular networks can be accessed at [26].

Statistical analyses of the chromatographic data proceeded with GraphPad Prism software (ver. 9.5.1, GraphPad Prism software Inc., La Jolla, CA, USA). The unpaired nonparametric Kruskal-Wallis tests, followed by Dunn’s post hoc tests, were used for the comparison of several groups. Differences between groups were considered statistically significant when *p* < 0.05. The log *P* values were calculated using the Chemistry Development Kit (CDK) ver. 2.8 [27].

### 2.5. LC-MS Analysis of the Eupentacta Fraudatrix Extract

Technical validation of the established database was achieved by using the spectral library to dereplicate the extract of the previously studied sea cucumber, *Eupentacta fraudatrix* (Djakonov et Baranova). Ten animals collected at Amursky Bay (Peter the Great Gulf, the Sea of Japan) in April 2017 at a depth of 1.0–1.5 m were chopped and extracted with 250 mL of ethanol. After filtration, the 2 mL of extracts were subjected to a drying process and later reconstituted in 1 mL of 50% methanol in water (*v*/*v*). Purification and desalting were performed by solid-phase extraction using an Oasis HLB extraction cartridge (60 mg, Waters, Medford, MA, USA). The sorbent was conditioned with 3 mL of methanol followed by 3 mL of water. The 250 μL of the extract was loaded into the SPE cartridge, and then the SPE cartridge was washed with 1 mL of water. Triterpene glycosides were eluted with 1 mL of 50% methanol and subjected to LC-MS analyses. The analysis conditions were the same as those described previously, except for the disable multiCE option. The LC-MS raw file was converted to mzML format using MSConvert and subsequently processed using MZmine 2.53, which allowed for the generation of an mgf file containing MS/MS data of the detected features (detailed MZmine parameters are provided as supplementary material (Table S2)), and the results were exported to GNPS for metabolite identification and FBMN analysis (parameters used were the same as those described above). The molecular network with the *E*. *fraudatrix* data can be accessed at [28].

## 3. Results and Discussion

A total of 15 sea cucumber species belonging to 5 families were the sources of the analyzed compounds (Table 1, Table S3). The family Cucumariidae was the most abundant, with 9 represented species yielding 68 triterpene glycosides. The families Psolidae and Sclerodactylidae were represented with 2 species each, providing 40 and 59 metabolites, respectively. The families Phyllophoridae and Caudinidae were represented by one species each. In addition, several triterpene glycosides were isolated from the starfish *Solaster pacificus* (*Solasteridae*, *Valvatida*).

The triterpene glycosides used in this study demonstrate a significant range of structural diversity, encompassing a broad spectrum of both oligosaccharide fragments and aglycons structures (the structures of all compounds are shown in Figures S1–S14). The set of triterpene glycosides analyzed included 61 non-sulfated and 130 sulfated metabolites, including 55 monosulfated, 43 disulfated, 23 trisulfated, and 9 of the rarest tetrasulfated compounds. The majority of glycosides (76 substances) contained oligosaccharide chains comprising four monosaccharide units, while the penta- and hexaoside groups consisted of 65 and 45 compounds, respectively. Additionally, five compounds had shorter oligosaccharide chains: three glycosides had three monosaccharide units each, and two compounds were biosides. The pool of aglycon structures comprised both compounds with a lactone cycle, including holostane derivatives with 18(20)-lactone (150 compounds) and the rarer 18(16)-lactone (16 compounds), as well as compounds without a lactone cycle (25 compounds). Some glycosides had oxygen-containing substituents in the polycyclic nuclei and the side chains, including acetoxy, hydroxy, and keto groups, as well as epoxy- and hydroperoxy groups.

Thus, the structures of the compounds used in this study encompass the majority of the known structural variants of sea cucumber triterpene glycosides, and the information acquired may facilitate the investigation of mass spectrometric behavior, fragmentation pathways, and the correlation of structures with retention times.

### 3.1. Analysis of the Mass Spectrometry Data

The established sea cucumber triterpene glycoside spectral library contains MS/MS spectra of 191 compounds. In addition, 494 MS/MS spectra obtained at various collision energies are available as Supplementary Material. Supplementary Table S3 presents a comprehensive list of the analyzed triterpene glycosides, along with their retention times and accurate mass measurements of precursor ions.

Although the positive ion mode provides intense B- and C-type product ions arising from the cleavages of glycosidic bonds (following the nomenclature of Domon and Costello [29]) in sulfated and non-sulfated compounds [30], the negative ion mode is generally more sensitive, especially for compounds with multiple sulfate groups. Furthermore, it is often unfeasible to obtain the molecular ion for glycosides containing more than two sulfate groups in the positive ion mode. In the negative ion mode, monosulfated and non-sulfated compounds were detected as [M−Na]^−^ and [M−H]^−^, respectively, whereas di-, tri-, and tetrasulfated glycosides produced multiply-charged ions, [M−nNa]^n−^, where n represents the number of sulfate groups.

The MS/MS spectra were acquired for each compound by applying three different collision energy levels (75, 100, and 125%), which allowed spectra with different degrees of precursor ion fragmentation to be collected. However, it should be noted that for many sulfated compounds, MS/MS spectra obtained at a collision energy level of 75% show only the precursor ion, and the most informative spectra were obtained at a collision energy level of 125%. Conversely, for non-sulfated compounds, a collision energy level of 75% (90 eV) was found to be optimal for obtaining informative MS/MS spectra. Higher energy values led to excessive fragmentation, resulting in uninformative or low-intensity spectra.

**Figure 2:**
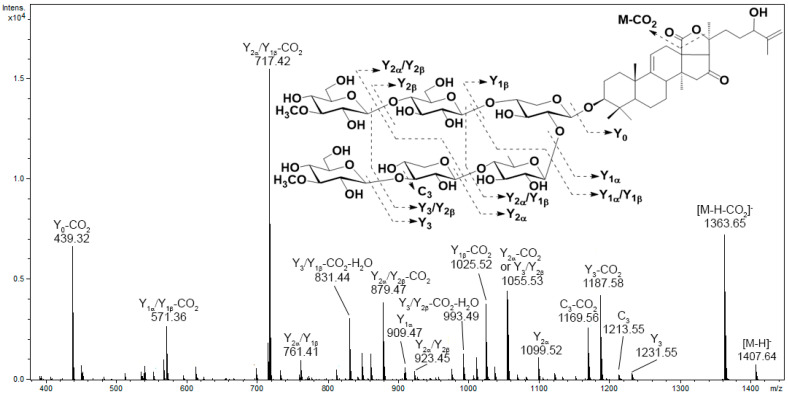
The MS/MS spectrum of the [M−H]^−^ precursor ion of psolusoside C_1_.

The majority of tandem mass spectra in the negative ion mode have included characteristic neutral losses of monosaccharide units (176 Da for MeGlc, 162 Da for Glc, 146 Da for Qui or MeXyl, and 132 Da for Xyl) and product ions arising from the polycyclic nucleus or side chain bond cleavage. The analysis of the obtained MS data allowed us to conclude the characteristic fragmentation pathways of triterpene glycosides under CID conditions. It was found that the presence and number of sulfate groups determine the optimal collision energy and primary fragmentation pathways. The product ion spectra of most non-sulfated triterpene glycosides contained an intense Y-type product ion series. In the MS/MS spectra of glycosides with a lactone cycle, ions related to the cleavage of lactone cycle bonds followed by the elimination of the CO_2_ molecule were observed. A neutral loss of 60 Da relates to the loss of an acetic acid molecule and is a characteristic of glycosides having an acetoxy group, while a neutral loss of 104 Da is indicative of the loss of a C_2_H_4_O_2_ + CO_2_ fragment and is a characteristic feature of glycosides that contain an acetoxy group and an 18(20)-lactone cycle [30]. In addition, some compounds tend to lose H_2_O molecules under CID conditions. For example, the product ion spectrum of [M−H]^−^ precursor ion at *m/z* 1407 of psolusoside C_1_ show fragment peaks at *m/z* 1363 [M−H−CO_2_]^−^, 1231 [M−H−MeGlc]^−^ (Y_3_ or Y_2β_), 1213 [M−H−MeGlc−H_2_O]^−^ (C_3_ or C_2β_), 1187 [M−H−MeGlc−CO_2_]^−^, 1169 [M−H−MeGlc−CO_2_−H_2_O]^−^, 1099 [M−H−MeGlc−Xyl]^−^ (Y_2α_), 1081 [M−H−MeGlc−Xyl−H_2_O]^−^, 1055 [M−H−MeGlc−Xyl−CO_2_]^−^, 1037 [M−H−MeGlc−Xyl−CO_2_−H_2_O]^−^ and similar series arising from loss CO_2_ and H_2_O molecules from fragment ions Y_1β_, Y_1α_, Y_3_/Y_2β_, Y_3_/Y_1β_, Y_2α_/Y_2β_, Y_2α_/Y_1β_, Y_1α_/Y_2β_, Y_1α_/Y_1β_ and Y_0_ (Figure 2). In addition, weakly characteristic B- and C-type product ion series were observed.

**Figure 3:**
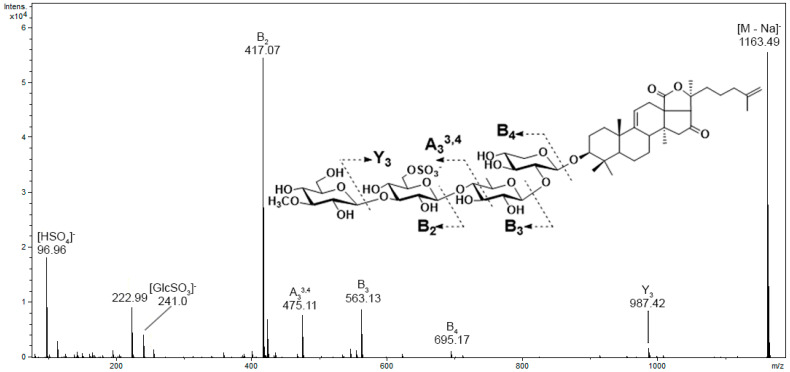
The MS/MS spectrum of the [M−Na]^−^ precursor ion of psolusoside E.

The product ion spectra of sulfated glycosides shows an intense diagnostic ion at *m/z* 96.96, which indicates the presence of a sulfate group. The MS/MS spectra also exhibited a product ion series arising from the cleavage of glycosidic bonds, with the charge localized on the sulfate group. Another fragmentation product specific for all sulfated glycosides were the ions associated with sulfated monosaccharide units at *m/z* 255, 241, and 211 for sulfated methylglucose, glucose, and xylose, respectively. For example, MS/MS spectra of the [M−Na]^−^ precursor ion at *m/z* 1163 of psolusoside E contain intensive fragment peaks at *m/z* 987 [M−Na−MeGlc]^−^, 695 [MeGlc+GlcSO_3_+Qui+Xyl−Na]^−^, 563 [MeGlc+GlcSO_3_+Qui−Na]^−^, 475 [MeGlc+GlcSO_3_+C_2_H_2_O_2_−Na]^−^ 417 [MeGlc+GlcSO_3_−Na]^−^, 241 [GlcSO_3_−Na]^−^, and 96.9 [HSO_4_]^−^ (Figure 3).

In addition to B- and Y-type series, in the product ion spectra of di-, tri-, and tetrasulfated glycosides A- and X-type product ion series, formed by the cross-ring cleavages of monosaccharide units, and ions associated with the elimination of additional sulfate groups were detected. For example, MS/MS spectra of [M−2Na]^2−^ precursor ion at *m/z* 621 of disulfated glycoside psolusoside A contain fragment peaks at *m/z* 1145 [M−2Na−HSO_4_]^−^, 987 [M−2Na−MeGlcSO_3_]^−^, 545 [MeGlcSO_3_+GlcSO_3_+Qui−2Na−HSO_4_]^−^, 521 [GlcSO_3_+Qui+Xyl−Na]^−^, 399 [MeGlcSO_3_+GlcSO_3_−2Na−HSO_4_]^−^, 387 [MeGlcSO_3_+GlcSO_3_+Qui+Xyl−2Na]^2−^, 321 [MeGlcSO_3_+GlcSO_3_+Qui−2Na]^2−^, 307 [MeGlcSO_3_+GlcSO_3_+C_5_H_10_O_3_−2Na]^2−^, 277 [MeGlcSO_3_+GlcSO_3_+C_3_H_6_O−2Na]^2−^, 255 [MeGlcSO_3_−Na]^−^, 248 [MeGlcSO_3_+GlcSO_3_−2Na]^2−^, and 96.9 [HSO_4_]^−^ (Figure S15). MS/MS spectra of the trisulfated glycoside quadrangularisoside D_2_ also shows the formation of B-, C-, Y-, and Z-type product ion series, as well as A_3_, A_4_, and X_2_ product ions resulting from cross-ring cleavages of the sulfated quinovose and xylose (Figure S16).

Thus, the information provided in CID spectra typically allows the straightforward determination of the structure and sequence of the glycone moiety. On the other hand, the elucidation of the structure of aglycon by fragmentation in CID spectra is often challenging because most of the glycosides exhibit limited aglycon fragmentation. However, detailed analysis and comparison of the fragmentation patterns of a series of monosulfated glycosides have resulted in the identification of characteristic structure-related neutral losses.

Cucumarioside H_7_ has a saturated side chain without substituents. The fragmentation of this compound shows a diagnostic series of neutral losses arising from cleavage of the C-20–C-22 bond (f-ions corresponding to the loss of the side chain, nomenclature proposed by Griffiths [31]), accompanied by the elimination of CO_2_, C_2_H_4_O_2_, and fragments of D-ring, as well as b-type ions produced by retro-Diels–Alder fragmentation of the B-ring (Table 2). Product ion spectra of lefevreoside B and typicoside A_1_ displayed similar series of neutral losses, but all masses of neutral losses were shifted by 2 and 4 Da, respectively, indicating one and two double bonds in the side chains of these molecules. The spectrum of cucumarioside H_5_, which has a 22Z,24-diene system in the side chain, shows the formation of a series of neutral losses that were identical to those of typicoside A_1_, which has a 22E,24-diene system in the side chain.

The most interesting fragmentation pattern was observed in the spectra of glycosides having an aglycon with Δ^25^ double bonds. The MS/MS spectra revealed the h-type fragment ions arising from the cleavage of the C-22–C-23 bond (Table 2). For example, in the product ion spectra of colochiroside A_1_ and lefevreoside C, which have aglycons with Δ^25^ double bonds, neutral losses similar to those in the product ion spectra of typicoside A_1_ and the fragment peak at *m/z* 1123 corresponding to the loss of the C_5_H_10_ fragment were observed. Similar h-type ions arising from the loss of 70 Da (C_5_H_10_) were observed in the MS/MS spectra of cucumarioside A_2_-2, philinopside E, and colochiroside A_2_, all of which contained a Δ^25^ double bond. These compounds, as well as colochiroside A_3_, do not have an acetoxy group at C-16, which is indicated by the shift in the mass of neutral losses of b_1_ and b_2_ ions and the production of a neutral loss of 170 Da (C_9_H_14_O_3_) (168 Da in colochiroside A_3_).

The spectrum of colochiroside B_1_, which has a 24-hydroxy-25-ene side chain, also shows neutral losses similar to those observed in the MS/MS spectrum of colochiroside A_1_, but masses of neutral losses were shifted by 16 Da. Colochiroside B_2_ and cucumarioside H_2_ show similar b_1_ and b_2_ ions but also exhibited neutral losses of 134 (C_6_H_14_O_3_) and 178 (C_7_H_14_O_5_) Da. Colochiroside B_3_ exhibited a neutral loss series shifted by 2 Da compared to that of colochiroside B_1_, which was caused by the replacement of a hydroxyl with a keto group. Product ion spectra of the glycosides with substitutions at C-23 (okhotoside A_1_-1 and cucumarioside A_0_-1 with 23-oxo side chains and frondoside D with 23-hydroxy side chains) also exhibited a similar characteristic series of neutral losses.

**Figure 4:**
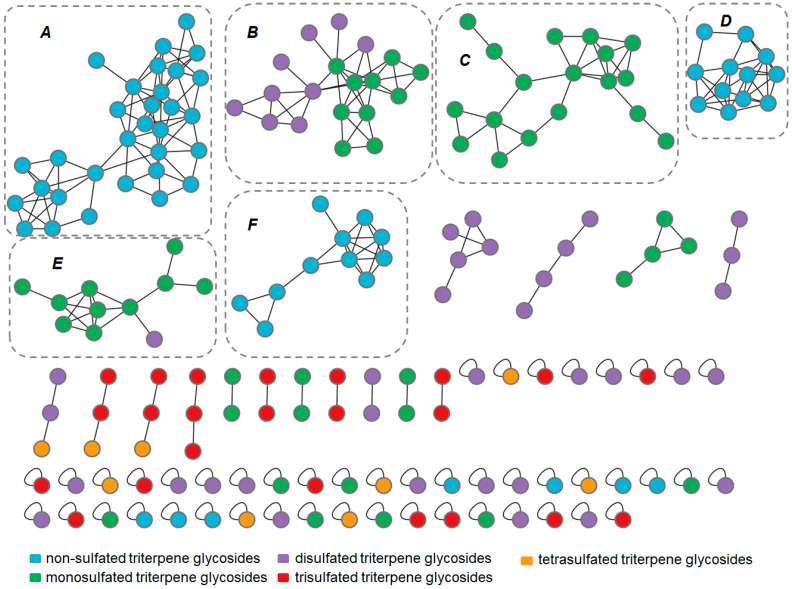
Molecular network of sea cucumber triterpene glycoside spectral library. Nodes were colored according to the number of sulfate groups in molecules.

Figure 4 shows the molecular networks generated with the sea cucumber triterpene glycosides spectral library as input. The resulting networks consisted of 834 edges and 191 nodes, which represent the analyzed compounds. Although the results were not homogenous, since the view depends on the cosine threshold definition and other parameters, some effect of compound structures on the topology of molecular networks can be observed. Overall, the obtained molecular networks tended to cluster triterpene glycosides according to several structural features. Thus, the number of sulfate groups had the strongest influence on clustering. All the non-sulfated compounds were split into three clusters, as were the monosulfated compounds, which were mostly in separate clusters. Most of the small clusters were represented by nodes related to tri- and tetrasulfated triterpene glycosides. Other structural features that influenced clustering were the number of monosaccharides, the number of acetate groups, and the structure of the side chains of the aglycons. Thus, clusters A and D (Figure 4) consisted mainly of non-sulfated cladolosides, but cluster A included cladolosides with two acetoxy groups at C-16 and C-22, whereas cluster D consisted of cladolosides without an acetoxy group. Cluster F contained the non-sulfated tetraosides, cucumariosides of the A-group. Clusters C and E consisted of monosulfated triterpene glycosides, whereas cluster B contained both mono- and disulfated compounds. Most of the compounds in cluster B were characterized by the presence of a Δ^25^ double bond, whereas the glycosides in cluster C had Δ^22^, Δ^23^, or Δ^24^ double bonds. Most triterpene glycosides forming cluster E had shortened side chains.

### 3.2. Analysis of the Chromatographic Behavior of Triterpene Glycosides

The analyzed metabolites had retention times that ranged from 4.3 to 18.9 min (Table S3). It should be noted that under the conditions used, chromatographic peaks were often observed to be broadened and asymmetrical for di- and trisulfated compounds. Reproducible elution profiles and retention times for tetrasulfated compounds were not achievable under the reversed-phase conditions used. Therefore, direct injection of the sample into the ESI ion source was applied for the analysis of psolusosides P and Q, chilensosides D, E, F, and G, and chitonoidosides K_1_ and L, along with two trisulfated quadrangularisosides D and D_1_.

Retention times in reversed-phase chromatography are known to be highly dependent on the lipophilicity of a compound. However, it was found that the correlation between retention times and log P, which may be considered a function of compound lipophilicity [32], is weak (R2 = 0.52) (Figure S19). Moreover, a considerable number of triterpene glycosides with similar calculated log *P* values displayed significant variations in retention times. For instance, cucumarioside A_14_ and chitonoidoside A, despite having comparable calculated log *P* values (−0.41 and −0.383), exhibited distinct chromatographic behaviors with retention times of 6.4 and 16.3 min, respectively. We have attempted to examine the effect of individual substituents and structural features on the chromatographic behavior of triterpene glycosides.

Compared to non-sulfated triterpene glycosides, sulfated compounds exhibited shorter mean retention times (*p* = 0.0002, Figure S20a). The appearance of the first sulfate group had no significant effect, but the presence of a di- or trisulfated oligosaccharide chain led to a significant reduction of retention time (*p* = 0.007 and *p* < 0.0001, respectively, Figure S20b). For example, the non-sulfated holotoxin A_1_ has a retention time of 13.1 min, while its monosulfated analog, cladoloside L_1_, has a retention time of 12.3 min. The addition of the second and third sulfate groups reduced the retention time more significantly. Monosulfated cucumarioside H_3_ exhibited a chromatographic peak at 8.8 min, whereas its disulfated analog, cucumarioside I_4_, had a retention time of 7.4 min. Similarly, disulfated chilensoside B displayed a peak at 10.8 min, whereas trisulfated analog chilensoside C eluted at 9.8 min.

The introduction of an additional monosaccharide unit caused a slight reduction in retention time (Figure S20c). For example, the appearance of the additional glucose unit in the hexasaccharide chain of kuriloside H (6.6 min) resulted in a small alteration of chromatographic behavior compared to its precursor, which has a pentasaccharide chain, kuriloside I_1_ (7.0 min). However, the impact became greater when a branching monosaccharide was added: the addition of xylose to C-2 of the quinovose residue of okhotoside A_1_-1 (12.3 min) reduced the retention time of the resulting pentaoside, cucumarioside A_0_-1 (11.2 min). Replacing a monosaccharide unit also resulted in an alteration in retention time, as was demonstrated by several examples. For instance, substituting the quinovose residue in cladoloside F_1_ with a xylose residue in cladoloside E_1_ caused a decrease in retention time of 0.6 min, from 15.1 to 14.5 min. In contrast, replacing two glucose units in cladoloside M_2_ with xylose units in cladoloside D_1_ increased the retention time from 12.1 to 13.3 min. These observations suggest that monosaccharide replacement increases retention time in the row glucose—xylose—quinovose.

The effect of the double bond position in the polycyclic nucleus was found to be negligible (Figure S20d), whereas the presence of a lactone cycle had a significant impact, particularly the formation of 18(20)-lactone, which led to an increase in retention time (*p* < 0.0001, Figure S20e). Cucumarioside A_9_, which has two hydroxy groups at positions C-20 and C-18, shows a chromatographic peak at 8.4 min. In contrast, its analog with an 18(20)-lactone cycle (cucumarioside A_7_) had a retention time of 9.7 min. The presence of acetoxy groups (especially two groups) was found to increase retention time (Figure S20f). For example, kurilosides K and K_1_, which differ in the presence of the acetoxy group at C-16, exhibited a notable difference in their chromatographic behavior, with retention times of 4.8 and 6.1 min, respectively. Similarly, cladoloside K_2_, which has an acetate group at C-16, had a retention time of 8.3 min, while its analog with an additional acetate group at C-22 (cladoloside K_1_) shows a shift in retention time to 12.7 min (similar to the case of cladolosides D_1_ and D_2_).

The elution of triterpene glycosides in reversed-phase conditions is significantly affected by the structure of the aglycon side chain and the presence of oxygen-containing substituents in the side chain (Figure S20g). The compounds with short side chains had the smallest retention times (e.g., fallaxosides C_1_, C_2_, D_2_, and D_7_), while cucumarioside A_3_ (with a butoxy-group attached to C-25) had the longest retention time. The addition of a double bond in the side chain did not result in a statistically significant change in retention time (Figure S20h), but when comparing the elution of similar compounds, it can be observed that glycosides with saturated side chains have longer retention times. Cucumarioside A_15_, which possesses a saturated side chain, exhibited a retention time of 18.7 min. In contrast, its analog, cucumarioside A_1_, containing a Δ^24^ double bond, exhibited a retention time of 17.1 min. The addition of a double bond at the C-25 position resulted in a smaller reduction of the retention time by approximately 1.1 min, as observed in rows of cladolosides E_1_–E_2_, F_1_–F_2_, P–P_1_, and D–D_1_. The position of the double bond seems to have little influence on retention time (the retention times of magnumosides A_3_ with Δ^25^ double bond and A_4_ with Δ^24^ double bond differ by 0.3 min). However, isomers with different double bond configurations in the side chain show distinct differences in chromatographic behavior. For example, compounds with a 22Z,24-diene fragment in the side chain (such as pacificusoside G and cucumarioside C_1_) elute about 0.4 min earlier than analogs with a 22E,24-diene fragment in the side chain (such as pacificusoside E and cucumarioside C_2_).

The presence of an oxygen-containing substituent in the side chain has been observed to significantly decrease the retention time (Figure S20i). The introduction of a hydroxyl group, a hydroperoxy group, and a keto group demonstrate the most significant impact on chromatographic behavior (Figure S20j). Conversely, the presence of an ester bond significantly increased the retention time. The presence of a hydroxy group at C-24 in magnumoside C_2_ (5.4 min) significantly changed the retention time compared to a similar compound without a substituent in the side chain (magnumoside C_3_, 9.1 min). The replacement of the hydroxyl group with a keto group also altered the retention time (e.g., frondoside D with a 23-hydroxy fragment in the side chain shows a peak at 9.1 min, while similar okhotoside A_1_-1 with a 23-oxo fragment in the side chain eluted at 12.3 min). The position of the oxygen-containing substituent did not strongly influence the chromatographic behavior, but metabolites with substituents at the C-25 position had longer retention times than their isomers with substituents at the C-24 position, e.g., quadrangularisosides A (9.0 min) and A_1_ (8.8 min).

Thus, the retention times of triterpene glycosides on reverse-phase chromatography are affected by various structural features, and structural changes have different effects on chromatographic behavior. Some of them, such as the presence of sulfate groups, changes in the aglycon side chain structure, lactone cycle formation, and the presence of substituents in the side chain, significantly affect the chromatographic behavior of triterpene glycosides, while other structural variations result in a small alteration.

### 3.3. LC-MS Analysis of the Eupentacta fraudatrix Extract

Technical validation was achieved by using the created spectral library to dereplicate triterpene glycosides in the extract of the previously studied Far Eastern sea cucumber, *E. fraudatrix*. Previous studies of the chemical composition of *E. fraudatrix* have led to the isolation of 37 triterpene glycosides, including 19 non-sulfated glycosides, 12 monosulfated glycosides, and 6 disulfated triterpene glycosides [30,33,34,35,36,37]. LC-ESI MS profiling of triterpene glycosides detected a total of 54 compounds, including 44 structurally annotated compounds, by comprehensive analysis of retention times, MS/MS data, elemental compositions, and biogenetic hypotheses. The identification of detected compounds was primarily based on the obtained data, including molecular formulae, MS/MS spectra, and chromatographic behavior. However, the structural identification was limited to the previously isolated compounds from this sea cucumber. The comparison with corresponding standards enabled the identification of 12 glycosides, while the remaining 8 glycosides were annotated based on elemental compositions and MS/MS data only [30].

LC-ESI MS profiling of *E. fraudatrix* followed by data processing in MZmine 2.53 yielded a total of 552 features. Dereplication of triterpene glycosides was achieved following two strategies: dereplication of the profiled compounds by GPNS against the mgf spectral library by MS data only, and identification of compounds against the local database by MZmine and Bruker Library Editor by comparison of MS/MS spectra and retention times with the data obtained for the authentic chemical standards.

The use of the MS data for library search enabled the annotation of 39 features. It is noteworthy that the metabolome of sea cucumbers displays a substantial prevalence of isomeric triterpene glycosides, with several of these isomers exhibiting minimal structural differences and having closely related MS/MS spectra. Relying solely on mass spectrometry data for identification purposes in such cases may lead to erroneous results. Indeed, the analyzed *E. fraudatrix* extract shows many isomeric triterpene glycosides with identical *m/z* and similar MS/MS spectra. Specifically, among five compounds with a molecular ion mass of *m/z* 1307, three were annotated as cucumarioside H_5_ based on the close similarity of their MS/MS spectra. Using retention times enabled a more accurate determination of the metabolites in the sample, resulting in their complete identification. Identification via a database containing not only mass spectra but also chromatographic data allowed us to refine results and identify 27 compounds (Table 3).

Among the identified metabolites, 16 compounds had previously been isolated from *E. fraudatrix*, including 7 non-sulfated glycosides (cucumariosides A_1_, A_7_, A_11_, A_15_, C_1_, C_2_, and D), 7 sulfated pentaosides (cucumariosides H_2_, H_3_, H_4_, H_5_, H_6_, and H_8_), and 2 disulfated pentaosides (cucumariosides I_3_ and I_4_). Additionally, 12 compounds that had been previously isolated from other species were identified for the first time in *E. fraudatrix*. These included colochirosides A_1_, B_1_, and B_2_, magnumoside B_3_, pacificusosides A, B, E, G, and J, quadrangularisoside A, and typicosides A_1_ and C_2_. It is noteworthy that the structures of these compounds corresponded to the common structural patterns observed in known triterpene glycosides of *E. fraudatrix*. According to the literature, the triterpene glycosides found in *E. fraudatrix* contain 3-*O*-MeXyl or 3-*O*-MeGlc as the terminal monosaccharide unit. Colochirosides A_1_, B_1_, B_2_, magnumoside B_3_, quadrangularisoside A, and typicoside A_1_ have a linear oligosaccharide chain with four monosaccharides—methylglucose as the terminal monosaccharide unit, xylose, quinovose, and sulfated xylose. Typicoside C_2_ has a similar oligosaccharide chain pattern, with sulfated glucose as the third unit. The non-sulfated glycosides, pacificusosides A and B, share a similar oligosaccharide chain pattern with known cucumariosides of the C-group, consisting of five monosaccharide units, including methylxylose, glucose, quinovose, and xylose in the main chain and xylose as the branching unit at the second monosaccharide. Pacificusoside J has a similar structure of an oligosaccharide chain with methylated glucose as the terminal monosaccharide unit. Pacificusosides G and E are tetraosides without terminal methylated units. Furthermore, it is worth noting that pacificusosides were previously found in the Far Eastern starfish *Solaster pacificus* and were considered food markers. The identification of pacificusosides in *E. fraudatrix* supports the hypothesis that these substances are obtained by the starfish through their diet and can persist in the starfish organism without undergoing significant metabolic transformations.

The sea cucumber metabolome is characterized by the presence of triterpene glycosides that share a common main structural pattern but vary in minor structural details. An approach based on identifying metabolites with similar MS/MS spectra and characterizing their structures via differences in their fragmentation patterns can significantly expand the scope of annotated metabolites in the LC-MS analysis of complex samples. Indeed, using the GPNS analog search tool for *E. fraudatrix* significantly extended the list of annotated metabolites, revealing 113 structural analogs that exhibit similar MS/MS fragmentation patterns to those of known compounds in the spectral library. Several typical mass differences were observed that correspond to specific changes in the structure of the compounds. An increase or decrease of *m/z* of the precursor ion by 176 Da corresponds to the methylglucose, while changes of 146 and 132 Da correspond to quinovose (or methylated xylose) and xylose units, respectively. An increase in mass of 58 Da corresponds to the addition of an acetate group, while a common difference of 30 Da suggests the substitution of glucose with xylose. Smaller differences of 18, 16, 14, 12, and 2 Da correspond to the loss or addition of H_2_O, O, CH_2_, C, and double bonds, respectively.

**Figure 5:**
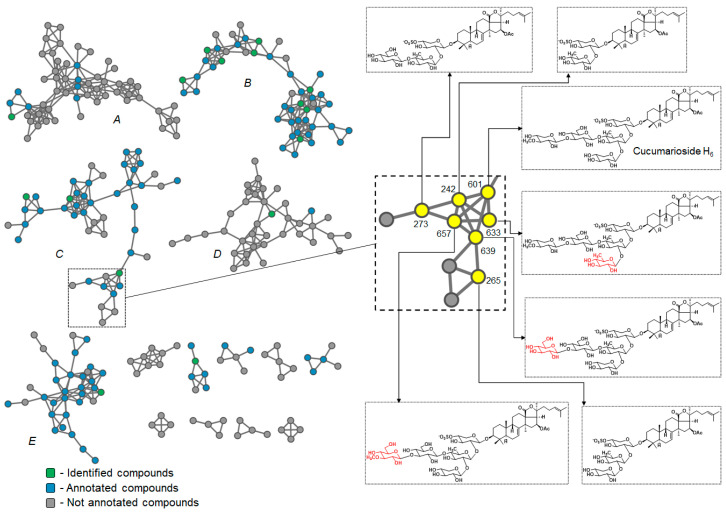
Molecular networks of the profiled compounds detected in *E. fraudatrix* extract annotated by the sea cucumber triterpene glycoside library.

To highlight the chemical diversity of *E. fraudatrix* extract, the molecular network was generated using the MS/MS spectra as input data. The resulting networks consisted of 834 edges and 552 nodes, which formed 14 clusters containing more than four nodes (Figure 5). The majority of the compounds were clustered in five clusters (A–E). Most of the identified and annotated features were found in clusters B, C, and E. Cluster B was mostly composed of non-sulfated triterpene glycosides, while clusters C and E predominantly comprised monosulfated glycosides. On the other hand, clusters A and D mostly consisted of unannotated metabolites, with cluster D mainly containing features related to disulfated triterpene glycosides.

Closely related compounds were found to be represented by closely spaced nodes in the molecular networks. For example, the node related to feature **601** was identified as cucumarioside H_6_, which had several neighbors that exhibited similar fragmentation patterns. The MS/MS spectra of six compounds (**242**, **265**, **273**, **633**, **639**, and **657**; the numbers correspond to the feature numbers defined by MZmine), as well as the MS/MS spectra of cucumarioside H_6_, revealed a neutral loss series of 128, 202, 228, 360, and 372 Da (Figure S21), which is characteristic for triterpene glycosides containing a holostane aglycon with a Δ^24^ side chain (Table 2). Furthermore, a characteristic fragment peak Y_1_ was detected at *m/z* 723 in the MS/MS spectra of all these compounds, indicating similar aglycon and sulfated xylose as the first monosaccharide unit. However, the masses of the molecular ions and the product ion series were shifted compared to cucucumarioside H_6_. The fragmentation patterns of compounds **242**, **265**, and **273** were shifted by 440, 308, and 278 Da, respectively, due to the loss of the monosaccharide units MeXyl, Glc, and Qui in **242**, MeXyl and Glc in **265**, and MeXyl and Qui in **273** compared to cucumarioside H_6_. The molecular masses as well as the product ion series in the MS/MS spectra of compounds **633**, **639**, and **657** were shifted by 14, 16, and 30 Da, corresponding to the additional fragments CH_2_, O, and CH_2_O. The MS/MS spectra of **633** exhibited a product ion at *m/z* 1177 related to loss of C_6_H_10_O_4_ (Qui or MeXyl), while a product ion [M−Na−132]^−^ related to loss of branching xylose was not observed. This finding, along with the increased retention time, indicates a replacement of the branching xylose unit with quinovose. The MS/MS spectra of **639** (*m/z* 1325 [M−Na]^−^) and **657** (*m/z* 1339 [M−Na]^−^) both produced ion Y_3_ at *m/z* 1163 associated with the loss of C_6_H_10_O_5_ (Glc) and C_7_H_12_O_5_ (MeGlc), respectively. These data, as well as the reduced retention time, indicate a replacement of the terminal MeXyl with Glc in **639** and with MeGlc in **657**. These data allow us to propose structures for triterpene glycosides **242**, **265**, **273**, **633**, **639**, and **657** (Figure 5).

Thus, the use of the obtained database of tandem mass spectra and retention times of 191 triterpene glycosides allowed the accurate dereplication of known triterpene glycosides and the annotation of some novel compounds. The example shown demonstrates the reliability of the database created, but at the same time, it has certain limitations that are common to such databases. The dataset is limited to the available compounds, but it will be expanded in the future as new compounds are isolated. Another limitation relates to data inconsistency. It is important to recognize that tandem mass spectra are heavily influenced by the instrument used and fragmentation settings, similar to how retention times are strongly influenced by the chromatographic setup and column used. Although the database includes MS/MS spectra obtained with different fragmentation energy settings, this limitation complicates spectral comparisons and may lead to the erroneous annotation of isomeric or structurally similar compounds.

## 4. Conclusions

This study presents a comprehensive database of sea cucumber triterpene glycosides, consisting of 191 compounds. The data were generated using ultra-performance liquid chromatography-quadrupole time-of-flight mass spectrometry and included retention times and MS/MS spectra. The used compounds were isolated from 15 species of the class Holothuroidea and one starfish species and comprise the majority of known structural variants of sea cucumber triterpene glycosides. By analyzing the fragmentation patterns, characteristic structure-related neutral losses were identified, which aided in the identification of isomeric and novel compounds. The retention time data provide important insights into the factors that influence the chromatographic behavior of triterpene glycosides in RP LC-MS analysis. This database represents a valuable resource for the identification and annotation of sea cucumber triterpene glycosides. To the best of our knowledge, this is the first MS database of sea cucumber triterpene glycosides. This resource will be highly valuable to researchers in the field of natural products, serving as a powerful tool for dereplication and streamlining the process of isolating novel bioactive compounds. The spectral library provided will greatly expedite the annotation of metabolites in MS-based research on echinoderms, including targeted or untargeted metabolomic studies, investigations into the biosynthesis and biological functions of triterpene glycosides, and chemotaxonomic analyses of newly reported or unstudied species. Additionally, the data obtained can serve as a reference dataset for investigating the MS fragmentation patterns of natural products, further enhancing research capabilities in this area.

## Figures and Tables

**Table 1:** Analyzed triterpene glycosides of sea cucumbers and starfish and their sources.

Order	Family	Species	Compounds
Dendrochirotida	Cucumariidae	*Actinocucumis typica*	Typicosides A_1_, A_2_, B_1_, C_1_, and C_2_
		*Colochirus quadrangularis*	Quadrangularisosides A, A_1_, B, B_1_, B_2_, C, C_1_, D, D_1_–D_4_, and E
		*Colochirus robustus*	Colochirosides A_1_–A_3_, B_1_–B_3_, C, D, and E; Hemoiedemoside B; Lefevreosides B and C; Neothyonidioside
		*Cucumaria djakonovi*	Cucumarioside A_0_-1; Frondoside D; Okhotoside A_1_-1
		*Cucumaria fallax*	Fallaxosides C_1_, C_2_, D_1_, D_2_, D_6_, and D_7_
		*Cucumaria japonica*	Cucumarioside A_2_-2
		*Pseudocolochirus violaceus*	Violaceusosides C, D, and E; Violaceusides II and A; Holothurinoside A; Liouvilloside A; Philinopside E
		*Staurocucumis turqueti*	Turquetoside A
		*Thyonidium* (=*Duasmodactyla*) *kurilensis*	Kurilosides A, A_1_-A_3_, C_1_, D, D_1_, E, F, G, H, I, I_1_, J, K, and K_1_; DS-Kurilosides L and M
	Phyllophoridae	*Neothynidium* (=*Massinium*) *magnum*	Magnumosides A_3_, A_4_, B_3_, and C_1_–C_4_
	Psolidae	*Psolus chitonoides*	Chitonoidosides A, A_1_, B C, D, E, E_1_, F, G, H, I, J, K, K_1_, and L
		*Psolus fabricii*	Psolusosides A, B, B_1_, B_2_, C_1_–C_3_, D_1_–D_5_, E, F, G, H, I, J, K, L, M, N, O, P, and Q
	Sclerodactylidae	*Cladolabes schmeltzii*	Cladolosides A_2_, B, B_1_, B_2_, C, C_1_, C_2_, D, D_1_, D_2_, E_1_, E_2_, F_1_, F_2_, G, H_1_, I_1_, I_2_, J_1_, K_1_, K_2_, L_1_, M, M_1_, M_2_, N, O, P, P_1_–P_3_, Q, and R; Holotoxin A_1_
		*Eupentacta fraudatrix*	Cucumariosides A_1_–A_4_, A_6_, A_7_, A_9_–A_15_, D, H_2_–H_8_, and I_1_–I_4_
Molpadida	Caudinidae	*Paracaudina chilensis*	Chilensosides A, A_1_, B, C, D, E, F, and G
Valvatida	Solasteridae	*Solaster pacificus*	Pacificusosides A, B, C, E, G, H, and J; Cucumariosides C_1_ and C_2_

**Table 2:** Fragmentation patterns related to the side chain structures of some monosulfated glycosides.

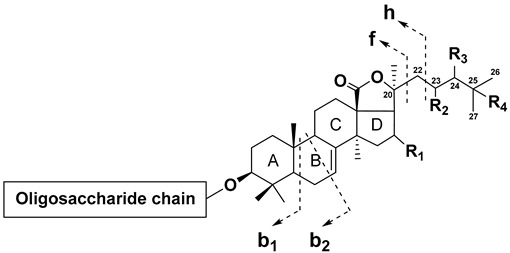												
R_1_	R_2_	R_3_	R_4_	Double Bonds	Neutral Losses, Da							Example Compound *
					b_1_	b_2_	f + C_6_H_8_O_4_	f + C_4_H_6_O_4_	f + C_3_H_4_O_4_	f + CO_2_	h	
OAc	H	H	H	-	374	362	230	204	190	130	-	Cucumarioside H_7_
OAc	H	H	H	Δ^24^	372	360	228	202	188	128	-	Lefevreoside B
OAc	H	H	H	Δ^22*E*,24^	370	358	226	200	-	-	-	Typicoside A_1_
OAc	H	H	H	Δ^22*Z*,24^	370	358	226	200	-	-	-	Cucumarioside H_5_
OAc	H	H	-	Δ^25^	372	360	228	202	188	128	70	Colochiroside A_1_
=O	H	H	-	Δ^25^	328	316	-	-	-	128	70	Philinopside E
H	H	H	-	Δ^25^	314	302	-	-	-	128	70	Colochiroside A_2_
H	H	H	H	Δ^24^	314	302	-	-	-	-	-	Colochiroside A_3_
OAc	H	OH	-	Δ^25^	388	376	244	218	204	144	86	Colochiroside B_1_
OAc	H	H	OH	Δ^23^	388	376	244	218	-	-	-	Colochiroside B_2_
OAc	H	=O	-	Δ^25^	386	374	242	216	202	142	84	Colochiroside B_3_
OAc	=O	H	H	-	388	376	244	218	204	144	-	Okhotoside A_1_-1
OAc	OH	H	H	-	390	378	246	220	206	-	-	Frondoside D

* The MS/MS spectra of the compounds are shown in Figures S17 and S18.

**Table 3:** Matches between profiled compounds detected in *E. fraudatrix* extract and the sea cucumber triterpene glycoside library.

Compound	Retention Time, min			*m/z* of Precursor Ion			Match Score
	Standard Compound	Features Detected in *E. fraudatrix*	Δ (min)	Calculated *m/z*	Measured *m/z*	Δ (ppm)	
Cucumarioside H_8_	6.3	6.3	0.0	1281.5216	1281.5135	6.3	0.930
Cucumarioside I_3_	6.7	6.6	0.1	702.2487	702.2474	1.7	0.752
Cucumarioside I_4_	7.4	7.4	0.0	630.2093	630.2104	−1.7	0.777
Cucumarioside H_2_	7.7	7.6	0.1	1325.5478	1325.5474	0.3	0.905
Colochiroside B_1_	8.1	8.1	0.0	1193.5055	1193.4989	5.6	0.801
Pacificusoside A	8.1	8.2	0.1	1201.5284	1201.5260	2.0	0.911
Colochiroside B_2_	8.3	8.3	0.0	1193.5055	1193.5030	2.1	0.936
Cucumarioside H_3_	8.8	8.8	0.0	1181.4691	1181.4680	1.0	0.954
Quadrangularisoside A	9.0	9.0	0.0	1209.5004	1209.4946	4.8	0.907
Cucumarioside A_7_	9.7	9.8	0.1	1113.5487	1113.5455	2.9	0.826
Cucumarioside A_11_	10.0	10.0	0.0	1113.5487	1113.5454	3.0	0.917
Pacificusoside J	10.1	10.1	0.0	1131.5229	1131.5194	3.1	0.951
Pacificusoside B	10.5	10.6	0.1	1101.5123	1101.5099	2.2	0.947
Magnumoside B_3_	10.6	10.8	0.2	1135.5000	1135.4929	6.3	0.889
Cucumarioside H_4_	11.7	11.5	0.2	1353.5791	1353.5759	2.3	0.941
Typicoside C_2_	12.0	12.0	0.0	643.2354	643.2363	−1.5	0.887
Cucumarioside H_5_	12.4	12.6	0.2	1307.5372	1307.5366	0.5	0.916
Colochiroside A_1_	13.2	12.9	0.3	1193.5051	1193.5055	−0.3	0.825
Cucumarioside H_6_	13.6	13.4	0.2	1309.5529	1309.5569	−3.1	0.892
Typicoside A_1_	13.7	13.7	0.0	1175.4950	1175.4933	1.4	0.800
Cucumarioside D	14.2	14.3	0.1	1257.5910	1257.5961	−4.1	0.763
Pacificusoside G	14.5	14.6	0.1	1081.5225	1081.5248	−2.1	0.820
Cucumarioside C_1_	14.7	14.8	0.1	1227.5804	1227.5812	−0.6	0.944
Pacificusoside E	14.9	15.0	0.1	1081.5225	1081.5195	2.7	0.975
Cucumarioside C_2_	15.1	15.2	0.1	1227.5804	1227.5812	−0.6	0.976
Cucumarioside A_1_	17.1	17.2	0.1	1097.5538	1097.5474	5.9	0.860
Cucumarioside A_15_	18.7	18.7	0.0	1099.5694	1099.5643	4.7	0.970

## Data Availability

The obtained data (raw scans in .mzML format, separate mgf files for every compound, a merged mgf file, and the Bruker .mlb spectral library) are available as Supplementary Materials. MS scans containing raw and derived MS and MS/MS spectra were also deposited on MetaboLights public repository (www.ebi.ac.uk/metabolights/MTBLS7506, accessed on 20 June 2023).

## Supplementary Materials

The following supporting information can be downloaded at: https://www.mdpi.com/article/10.3390/metabo13070783/s1, Table S1: Mass spectra acquisition parameters; Table S2: The batch steps and parameters used for data preprocessing in MZmine; Table S3: List of the analyzed triterpene glycosides and their corresponding data obtained by LC-MS; Figure S1: Structures of cucumarioside A2-2 and psolusosides B, B1, B2, C1–C3, D1-D5, and E; Figure S2: Structures of psolusosides F, G, H, I, J, K, L, M, N, O, P, and Q, and kurilosides A and A1; Figure S3: Structures of kurilosides A2, A3, C1, D, D1, E, F, G, H, I, I1, J, K, and K1; Figure S4: Structures of DS-kurilosides L and M and quadrangularisosides A, A1, B, B1, B2, C, C1, D, and D1–D4; Figure S5: Structures of quadrangularisoside E, chilensosides A, A1, B, C, D, E, F, and G, and chitonoidosides A, A1, B, C, and D; Figure S6: Structures of chitonoidosides E, E1, F, G, H, I, J, K, K1, and L and magnumosides A3, A4, B3, and C1; Figure S7: Structures of magnumosides C2–C4, colochirosides A1–A3, B1–B3, C, D, and E and neothyonidioside and lefevreoside B; Figure S8: Structures of hemoiedemoside B, lefevreoside C, and typicosides A1, A2, B1, C1, and C2, fallaxosides C1, C2, D1, D2, D6, and D7, and violaceuside A; Figure S9: Structures of violaceusosides C, D, and E, violaceuside II, holothurinoside A, liouvilloside A, philinopside E, and cladolosides A2, B, B1, B2, C, C1, and C2; Figure S10: Structures of cladolosides D, D1, D2, E1, E2, F1, F2, G, H1, I1, I2, J1, K1, and K2; Figure S11: Structures of cladolosides L1, M, M1, M2, N, O, P, P1–P3, Q, and R, holotoxin A1 and cucumarioside A1; Figure S12: Structures of cucumariosides A2–A4, A6, A7, A9–A15, D, and H2; Figure S13: Structures of cucumariosides H3–H8, and I1–I4, cucumarioside A0-1, and frondoside D; Figure S14: Structures of okhotoside A1-1, turquetoside A, cucumariosides C1 and C2, and pacificusosides A, B, C, E, G, H, and J; Figure S15: The MS/MS spectrum of the [M−2Na]2− precursor ion of psolusoside A; Figure S16: The MS/MS spectrum of the [M−3Na]3− precursor ion of quadrangularisoside D2; Figure S17: The MS/MS spectra of the [M–Na]− precursor ions of cucumarioside H7: (a), lefevreoside B (b), typicoside A1 (c), cucumarioside H5 (d), colochiroside A1 (e), and philinopside E (f); Figure S18: The MS/MS spectra of the [M–Na]− precursor ions of colochiroside A2: (a), colochiroside A3 (b), colochiroside B1 (c), colochiroside B2 (d), colochiroside B3 (e), okhotoside A1-1 (f), and frondoside D (g); Figure S19: The plot of log P vs. retention time of triterpene glycosides analyzed by LC-MS; Figure S20: Variations in the retention times of triterpene glycosides related to some structural features: (a) the presence of a sulfate group; (b) the number of sulfate groups; (c) the number of monosaccharide units; (d) the position of the double bond in the polycyclic nucleus of the aglycone; (e) the presence and the type of a lactone cycle; (f) the number of acetoxy groups; (g) the number of carbon atoms in the side chain; (h) the number of double bonds in the side chain; (i) the number of oxygen-containing substituents in the side chain; and (j) the type of oxygen-containing substituent in the side chain. Asterisks (* p < 0.05, ** p < 0.01, *** p < 0.001 **** p < 0.0001) indicate significant differences between groups; Figure S21: The MS/MS spectra of the [M–Na]− precursor ions of cucumarioside H6 (601), and structure-related compounds detected in E. fraudatrix extract (242, 265, 273, 633, 639, and 357); Supplementary Materials S2: MS raw and derived scans; Supplementary Materials S3: Sea cucumber triterpene glycoside spectral library.
