# Supplementary material for: A Mass Spectrometry Database for Sea Cucumber Triterpene Glycosides
Source: Metabolites. 2023 Jun 23;13(7):783. doi: 10.3390/metabo13070783 (PMC10384350; doi:10.3390/metabo13070783)
Supplement: Supplementary file 1 [file metabolites-13-00783-s001.zip › metabolites-2359027_Supplementary Materials.pdf]

Supplementary Materials:

Article

A Mass Spectrometry Database for Sea Cucumber Triterpene Glycosides

Roman S. Popov *, Natalia V. Ivanchina, Alexandra S. Silchenko, Sergey A. Avilov, Vladimir I. Kalinin, Timofey V. Malyarenko, Valentin A. Stonik and Pavel S. Dmitrenok *

G.B. Elyakov Pacific Institute of Bioorganic Chemistry, Far Eastern Branch of Russian Academy of Sciences, 159 Prospect 100-let Vladivostoku, Vladivostok 690022, Russia; ivanchina@piboc.dvo.ru (N.V.I.); silchenko_als@piboc.dvo.ru (A.S.S.); avilov_sa@piboc.dvo.ru (S.A.A.); kalininv@piboc.dvo.ru (V.I.K.); malyarenko-tv@mail.ru (T.V.M.); stonik@piboc.dvo.ru (V.A.S.)

* Correspondence: popov_rs@piboc.dvo.ru (R.S.P.); paveldmt@piboc.dvo.ru (P.S.D.); Tel.: +7-423-231-1132 (P.S.D.)

Contents

Table S1. Mass spectra acquisition parameters.

Table S2. The batch steps and parameters used for data preprocessing in MZmine.

Table S3. List of analyzed triterpene glycosides and their corresponding data obtained by LC-MS.

Figure S1. Structures of cucumarioside A_2-2, psolusosides B, B_1, B_2, C_1–C_3, D_1-D_5, and E.

Figure S2. Structures of psolusosides F, G, H, I, J, K, L, M, N, O, P, and Q, kurilosides A and A_1.

Figure S3. Structures of kurilosides A_2, A_3, C_1, D, D_1, E, F, G, H, I, I_1, J, K, and K_1.

Figure S4. Structures of DS-kurilosides L and M, quadrangularisosides A, A_1, B, B_1, B_2, C, C_1, D, and D_1–D_4.

Figure S5. Structures of quadrangularisoside E, chilensosides A, A_1, B, C, D, E, F, and G, and chitonoidosides A, A_1, B, C, and D.

Figure S6. Structures of chitonoidosides E, E_1, F, G, H, I, J, K, K_1, and L, magnumosides A_3, A_4, B_3, and C_1.

Figure S7. Structures of magnumosides C_2–C_4, colochirosides A_1–A_3, B_1–B_3, C, D, and E, neothyonidioside and lefevreoside B.

Figure S8. Structures of hemoiedemoside B, lefevreoside C, typicosides A_1, A_2, B_1, C_1, and C_2, fallaxosides C_1, C_2, D_1, D_2, D_6, and D_7 and violaceuside A.

Figure S9. Structures of violaceusosides C, D, and E, violaceuside II, holothurinoside A, liouvilloside A, philinopside E and cladolosides A_2, B, B_1, B_2, C, C_1, and C_2.

Figure S10. Structures of cladolosides D, D_1, D_2, E_1, E_2, F_1, F_2, G, H_1, I_1, I_2, J_1, K_1, and K_2.

Figure S11. Structures of cladolosides L_1, M, M_1, M_2, N, O, P, P_1–P_3, Q, and R, holotoxin A_1 and cucumarioside A_1.

Figure S12. Structures of cucumariosides A_2–A_4, A_6, A_7, A_9–A_{15}, D, and H_2.

Figure S13. Structures of cucumariosides H_3–H_8, and I_1–I_4, cucumarioside A_0-1 and frondoside D.

Figure S14. Structures of okhotoside A_1-1, turquetoside A, cucumariosides C_1 and C_2, pacificusosides A, B, C, E, G, H, and J.

Figure S15. The MS/MS spectrum of $[M-2Na]^{2-}$ precursor ion of psolusoside A.

Figure S16. The MS/MS spectrum of $[M{-}3Na]^{3-}$ precursor ion of quadrangularisoside D_2.

Figure S17. The MS/MS spectra of $[M{-}Na]^-$ precursor ions of cucumarioside H_7 (a), lefevreoside B (b), typicoside A_1 (c), cucumarioside H_5 (d), colochiroside A_1 (e), and philinopside E (f).

Figure S18. The MS/MS spectra of $[M{-}Na]^-$ precursor ions of colochiroside A_2 (a), colochiroside A_3 (b), colochiroside B_1 (c), colochiroside B_2 (d), colochiroside B_3 (e), okhotoside A_1-1 (f), and frondoside D (g).

Figure S19. The plot of log P vs. retention time of triterpene glycosides analyzed by LC-MS.

Figure S20. Variations in the retention times of triterpene glycosides related to some structural features: (a) the presence of a sulfate group; (b) the number of sulfate groups; (c) the number of monosaccharide units; (d) the position of the double bond in the polycyclic nucleus of the aglycone; (e) the presence and the type of a lactone cycle; (f) the number of acetoxy groups; (g) the number of carbon atoms in the side chain; (h) the number of double bonds in the side chain; (i) the number of oxygen-containing substituents in the side chain; (j) the type of oxygen-containing substituent in the side chain. Asterisks (* $p < 0.05$, ** $p < 0.01$, *** $p < 0.001$ **** $p < 0.0001$) indicate significant differences between groups.

Figure S21. The MS/MS spectra of $[M{-}Na]^-$ precursor ions of cucumarioside H_6 (**601**) and structure-related compounds detected in *E. fraudatrix* extract (**242**, **265**, **273**, **633**, **639**, **357**).

Table S1. Mass spectra acquisition parameters.

Type	Parameter	Value
Main	Polarity	Negative
	Mass Range	80–2000
	Scan Mode	Auto MS/MS
	Spectra rate	1.50 Hz
	Rolling Average	Off
Source	Capillary	4000 V
	End Plate Offset	500 V
	Dry Gas	6 L/min
	Nebulizer	2.5 bar
	Dry Temperature	215 °C
Tune	Funnel 1 RF	300.0 Vpp
	Funnel 2 RF	400.0 Vpp
	isCID Energy	90.0 eV
	Hexapole RF	80.0 Vpp
	Ion Energy	5.0 eV
	Low Mass	100.0 *m*/*z*
	Collision Energy	10.0 eV
	Collision RF	700.0 Vpp
	Transfer Time	110.0 µs
	Pre Pulse Storage	8.0 µs
MS/MS	Threshold (per 1000 sum.)	700 cts.
	Active Exclusion	On
	Exclude after	3 Spectra
	No. of Precursors	1
	Exclude Mass List	80-350 *m*/*z*
	Auto MS/MS Multi CE	On
	Collision Energy List for separate spectra acquisition	75, 100, 125%
	Collision Energy at Charge State of 1	120 eV
	Collision Energy at Charge State of 2	60 eV
	Collision Energy at Charge State of 3	43 eV
	Collision Energy at Charge State of 4	40 eV
	CID Acquisition with precursor ion intensity lower than 1500 cts.	1.0 Hz
	CID Acquisition with precursor ion intensity higher than 50000 cts.	3.0 Hz

Table S2. The batch steps and parameters used for data preprocessing in MZmine.

Batch step	Module	Parameters	Value
Raw data import	—	—	—
Mass detection	Wavelet transform	MS1 Noise level	50
		MS2 Noise level	20
		Scale level	5
		Wavelet window size	50%
	Shoulder peaks filter	Peak model function	Lorentzian extended
Chromatogram detection	ADAP Chromatogram Builder	Min group size in number of scans	6
		Group intensity threshold	130
		Min highest intensity	250
		m/z tolerance	0.02 *m/z*
Chromatogram deconvolution	Wavelets (ADAP)	S/N Threshold	8
		Min feature height	250
		Coefficient/area threshold	40
		Peak duration range	0.05–2.0 min
		RT wavelet range	0.0–0.05 min
Isotopic peak removal	Isotopic peaks grouper	*m/z* tolerance	0.03 *m/z*
		Retention time tolerance	0.1 min
Filtering	Feature list row filter	Keep only peaks with MS2 scan	
Export results	Export/Submit to GNPS-FBMN with Merge MS/MS	Select spectra to merge	same sample
		m/z merge mode	most intense
		Intensity merge mode	sum intensities
		Expected mass deviation	0.05 *m/z*
		Cosine threshold	70%
		Peak count threshold	20%
		Isolation window offset	0
		Isolation window width	3

Table S3. List of analyzed triterpene glycosides and their corresponding data obtained by LC-MS.

No.	Compound	Elemental Composition	Rt (min)	Precursor ion				Species (Family, Order)	Source article DOI
				Calculated *m/z*	Molecular Ion Type	Measured *m/z*	Δ, ppm		
1	Cucumarioside A_2-2	$C_{59}H_{91}O_{29}SNa$	12.7	1295.5372	$[M-Na]^-$	1295.5353	1.5	*Cucumaria japonica* (Cucumariidae, Dendrochirotida)	10.1007/BF00580052
2	Psolusoside A	$C_{54}H_{82}O_{28}S_2Na_2$	12.6	621.2223	$[M-2Na]^{2-}$	621.2271	-7.8	*Psolus fabricii* (Psolidae, Dendrochirotida)	10.3390/md17060358
3	Psolusoside B	$C_{55}H_{84}O_{30}S_2Na_2$	9.7	644.2250	$[M-2Na]^{2-}$	644.2213	5.7	*Psolus fabricii* (Psolidae, Dendrochirotida)	10.3390/md17060358
4	Psolusoside B_1	$C_{55}H_{82}O_{31}S_2Na_2$	8.0	651.2146	$[M-2Na]^{2-}$	651.2141	0.8	*Psolus fabricii* (Psolidae, Dendrochirotida)	10.3390/md17110631
5	Psolusoside B_2	$C_{55}H_{82}O_{31}S_2Na_2$	8.0	651.2146	$[M-2Na]^{2-}$	651.2133	2.0	*Psolus fabricii* (Psolidae, Dendrochirotida)	10.3390/md17110631
6	Psolusoside C_1	$C_{66}H_{104}O_{32}$	7.3	1407.6438	$[M-H]^-$	1407.6398	2.8	*Psolus fabricii* (Psolidae, Dendrochirotida)	10.1177/1934578x1801301213
7	Psolusoside C_2	$C_{66}H_{102}O_{32}$	7.1	1405.6281	$[M-H]^-$	1405.6237	3.2	*Psolus fabricii* (Psolidae, Dendrochirotida)	10.1177/1934578x1801301213
8	Psolusoside C_3	$C_{66}H_{104}O_{32}$	7.4	1407.6438	$[M-H]^-$	1407.6392	3.3	*Psolus fabricii* (Psolidae, Dendrochirotida)	10.1177/1934578X19861253
9	Psolusoside D_1	$C_{67}H_{106}O_{32}$	13.1	1421.6594	$[M-H]^-$	1421.6532	4.4	*Psolus fabricii* (Psolidae, Dendrochirotida)	10.1177/1934578x1801301213
10	Psolusoside D_2	$C_{67}H_{104}O_{33}$	8.8	1435.6387	$[M-H]^-$	1435.6347	2.8	*Psolus fabricii* (Psolidae, Dendrochirotida)	10.1177/1934578X19861253
11	Psolusoside D_3	$C_{67}H_{106}O_{34}$	8.4	1453.6493	$[M-H]^-$	1453.6481	0.8	*Psolus fabricii* (Psolidae, Dendrochirotida)	10.1177/1934578X19861253
12	Psolusoside D_4	$C_{67}H_{106}O_{33}$	7.1	1437.6544	$[M-H]^-$	1437.6487	3.9	*Psolus fabricii* (Psolidae, Dendrochirotida)	10.1177/1934578X19861253
13	Psolusoside D_5	$C_{67}H_{106}O_{33}$	7.0	1437.6544	$[M-H]^-$	1437.6492	3.6	*Psolus fabricii* (Psolidae, Dendrochirotida)	10.1177/1934578X19861253
14	Psolusoside E	$C_{54}H_{83}O_{25}SNa$	14.5	1163.4950	$[M-Na]^-$	1163.4899	4.4	*Psolus fabricii* (Psolidae, Dendrochirotida)	10.3390/md17060358
15	Psolusoside F	$C_{54}H_{83}O_{25}SNa$	14.6	1163.4950	$[M-Na]^-$	1163.4895	4.7	*Psolus fabricii* (Psolidae, Dendrochirotida)	10.3390/md17060358
16	Psolusoside G	$C_{54}H_{82}O_{29}S_2Na_2$	11.7	629.2197	$[M-2Na]^{2-}$	629.2166	4.9	*Psolus fabricii* (Psolidae, Dendrochirotida)	10.3390/md17060358
17	Psolusoside H	$C_{47}H_{71}O_{21}SNa$	13.7	1003.4214	$[M-Na]^-$	1003.4161	5.3	*Psolus fabricii* (Psolidae, Dendrochirotida)	10.3390/md17060358
18	Psolusoside I	$C_{54}H_{82}O_{29}S_2Na_2$	10.5	629.2197	$[M-2Na]^{2-}$	629.2174	3.7	*Psolus fabricii* (Psolidae, Dendrochirotida)	10.3390/md17060358
19	Psolusoside J	$C_{53}H_{79}O_{32}S_3Na_3$	8.8	441.1244	$[M-3Na]^{3-}$	441.1243	0.3	*Psolus fabricii* (Psolidae, Dendrochirotida)	10.3390/md17110631
20	Psolusoside K	$C_{53}H_{79}O_{32}S_3Na_3$	8.0	441.1244	$[M-3Na]^{3}-$	441.1244	0.1	*Psolus fabricii* (Psolidae, Dendrochirotida)	10.3390/md17110631
21	Psolusoside L	$C_{60}H_{91}O_{36}S_3Na_3$	9.5	494.4823	$[M-3Na]^{3-}$	494.4804	3.8	*Psolus fabricii* (Psolidae, Dendrochirotida)	10.3390/md17110631
22	Psolusoside M	$C_{60}H_{91}O_{36}S_3Na_3$	9.2	494.4823	$[M-3Na]^{3-}$	494.4795	5.6	*Psolus fabricii* (Psolidae, Dendrochirotida)	10.3390/md17110631
23	Psolusoside N	$C_{60}H_{91}O_{37}S_3Na_3$	8.6	499.8139	$[M-3Na]^{3-}$	499.8126	2.7	*Psolus fabricii* (Psolidae, Dendrochirotida)	10.3390/md17110631
24	Psolusoside O	$C_{60}H_{91}O_{37}S_3Na_3$	8.2	499.8139	$[M-3Na]^{3-}$	499.8117	4.5	*Psolus fabricii* (Psolidae, Dendrochirotida)	10.3390/md17110631
25	Psolusoside P	$C_{60}H_{90}O_{39}S_4Na_4$	- [a]	390.5991	$[M-4Na]^{4-}$	390.6001	-2.6	*Psolus fabricii* (Psolidae, Dendrochirotida)	10.3390/md17110631

26	Psolusoside Q	$C_{60}H_{90}O_{40}S_4Na_4$	- [a]	394.5978	$[M-4Na]^{4-}$	394.5989	-2.7	*Psolus fabricii* (Psolidae, Dendrochirotida)	10.3390/md17110631
27	Kuriloside A	$C_{56}H_{89}O_{30}SNa$	7.5	1273.5165	$[M-Na]^{-}$	1273.5144	1.6	*Thyonidium* (=*Duasmodactyla*) *kurilensis* (Cucumariidae, Dendrochirotida)	10.3390/md18110551
28	Kuriloside A1	$C_{58}H_{93}O_{31}SNa$	9.7	1317.5427	$[M-Na]^{-}$	1317.5413	1.1	*Thyonidium kurilensis* (Cucumariidae, Dendrochirotida)	10.3390/md18110551
29	Kuriloside A2	$C_{54}H_{85}O_{28}SNa$	9.5	1213.4954	$[M-Na]^{-}$	1213.4920	2.8	*Thyonidium kurilensis* (Cucumariidae, Dendrochirotida)	10.3390/md18110551
30	Kuriloside A3	$C_{54}H_{87}O_{29}SNa$	5.1	1231.5059	$[M-Na]^{-}$	1231.5036	1.9	*Thyonidium kurilensis* (Cucumariidae, Dendrochirotida)	10.3390/md19040187
31	Kuriloside C1	$C_{52}H_{83}O_{26}SNa$	12.0	1155.4899	$[M-Na]^{-}$	1155.4891	0.7	*Thyonidium kurilensis* (Cucumariidae, Dendrochirotida)	10.3390/md18110551
32	Kuriloside D	$C_{66}H_{105}O_{35}SNa$	9.0	1489.6163	$[M-Na]^{-}$	1489.6133	2.0	*Thyonidium kurilensis* (Cucumariidae, Dendrochirotida)	10.3390/md18110551
33	Kuriloside D1	$C_{66}H_{107}O_{36}SNa$	5.3	1507.6268	$[M-Na]^{-}$	1507.6279	-0.7	*Thyonidium kurilensis* (Cucumariidae, Dendrochirotida)	10.3390/md19040187
34	Kuriloside E	C54$H_{87}O_{29}SNa$	8.1	1231.5059	$[M-Na]^{-}$	1231.5034	2.0	*Thyonidium kurilensis* (Cucumariidae, Dendrochirotida)	10.3390/md18110551
35	Kuriloside F	$C_{61}H_{99}O_{34}SNa$	5.1	1407.5744	$[M-Na]^{-}$	1407.5736	0.6	*Thyonidium kurilensis* (Cucumariidae, Dendrochirotida)	10.3390/md18110551
36	Kuriloside G	$C_{61}H_{98}O_{37}S_2Na_2$	4.7	743.2620	$[M-2Na]^{2-}$	743.2591	3.9	*Thyonidium kurilensis* (Cucumariidae, Dendrochirotida)	10.3390/md19040187
37	Kuriloside H	$C_{64}H_{101}O_{42}S_3Na_3$	6.6	545.8315	$[M-3Na]^{3-}$	545.8299	3.0	*Thyonidium kurilensis* (Cucumariidae, Dendrochirotida)	10.3390/md19040187
38	Kuriloside I	$C_{54}H_{87}O_{35}S_3Na_3$	4.6	463.8069	$[M-3Na]^{3-}$	463.8062	1.5	*Thyonidium kurilensis* (Cucumariidae, Dendrochirotida)	10.3390/md19040187
39	Kuriloside I1	$C_{58}H_{91}O_{37}S_3Na_3$	7.0	491.8139	$[M-3Na]^{3-}$	491.8139	0.1	*Thyonidium kurilensis* (Cucumariidae, Dendrochirotida)	10.3390/md19040187
40	Kuriloside J	$C_{56}H_{90}O_{33}S_2Na_2$	6.1	677.2408	$[M-2Na]^{2-}$	677.2393	2.3	*Thyonidium kurilensis* (Cucumariidae, Dendrochirotida)	10.3390/md19040187
41	Kuriloside K	$C_{54}H_{88}O_{32}S_2Na_2$	4.8	656.2356	$[M-2Na]^{2-}$	656.2340	2.4	*Thyonidium kurilensis* (Cucumariidae, Dendrochirotida)	10.3390/md19040187
42	Kuriloside K1	$C_{56}H_{90}O_{33}S_2Na_2$	6.1	677.2408	$[M-2Na]^{2-}$	677.2392	2.4	*Thyonidium kurilensis* (Cucumariidae, Dendrochirotida)	10.3390/md19040187
43	DS-Kuriloside M	$C_{54}H_{88}O_{26}$	5.7	1151.5491	$[M-H]^{-}$	1151.5529	-3.3	*Thyonidium kurilensis* (Cucumariidae, Dendrochirotida)	10.3390/md19040187
44	DS-Kuriloside L	$C_{41}H_{64}O_{15}$	13.5	795.4172	$[M-H]^{-}$	795.4201	-3.6	*Thyonidium kurilensis* (Cucumariidae, Dendrochirotida)	10.3390/md19040187
45	Quadrangularisoside A	$C_{55}H_{85}O_{27}SNa$	9.0	1209.5004	$[M-Na]^{-}$	1209.4967	3.1	*Colochirus quadrangularis* (Cucumariidae, Dendrochirotida)	10.3390/md18080394
46	Quadrangularisoside A1	$C_{55}H_{85}O_{27}SNa$	8.8	1209.5004	$[M-Na]^{-}$	1209.4947	4.7	*Colochirus quadrangularis* (Cucumariidae, Dendrochirotida)	10.3390/md18080394
47	Quadrangularisoside B	$C_{55}H_{84}O_{28}S_2Na_2$	12.9	628.2301	$[M-2Na]^{2-}$	628.2276	3.9	*Colochirus quadrangularis* (Cucumariidae, Dendrochirotida)	10.3390/md18080394
48	Quadrangularisoside B1	$C_{55}H_{84}O_{28}S_2Na_2$	12.6	628.2301	$[M-2Na]^{2-}$	628.2294	1.1	*Colochirus quadrangularis* (Cucumariidae, Dendrochirotida)	10.3390/md18080394
49	Quadrangularisoside B2	$C_{53}H_{80}O_{27}S_2Na_2$	12.3	606.2170	$[M-2Na]^{2-}$	606.2153	2.8	*Colochirus quadrangularis* (Cucumariidae, Dendrochirotida)	10.3390/md18080394
50	Quadrangularisoside C	$C_{56}H_{86}O_{29}S_2Na_2$	12.3	643.2354	$[M-2Na]^{2-}$	643.2342	1.8	*Colochirus quadrangularis* (Cucumariidae, Dendrochirotida)	10.3390/md18080394
51	Quadrangularisoside C1	$C_{56}H_{88}O_{29}S_2Na_2$	13.6	644.2432	$[M-2Na]^{2-}$	644.2417	2.3	*Colochirus quadrangularis* (Cucumariidae, Dendrochirotida)	10.3390/md18080394
52	Quadrangularisoside D	$C_{55}H_{83}O_{31}S_3Na_3$	- [a]	445.1366	$[M-3Na]^{3-}$	445.1387	-4.8	*Colochirus quadrangularis* (Cucumariidae, Dendrochirotida)	10.3390/md18080394
53	Quadrangularisoside D1	$C_{55}H_{83}O_{31}S_3Na_3$	- [a]	445.1366	$[M-3Na]^{3-}$	445.1390	-5.5	*Colochirus quadrangularis* (Cucumariidae, Dendrochirotida)	10.3390/md18080394

54	Quadrangularisoside D_2	$C_{53}H_{79}O_{30}S_3Na_3$	10.4	430.4612	$[M-3Na]^{3-}$	430.4629	-4.0	*Colochirus quadrangularis* (Cucumariidae, Dendrochirotida)	10.3390/md18080394
55	Quadrangularisoside D_3	$C_{55}H_{83}O_{33}S_3Na_3$	6.9	455.7998	$[M-3Na]^{3-}$	455.8012	-3.0	*Colochirus quadrangularis* (Cucumariidae, Dendrochirotida)	10.3390/md18080394
56	Quadrangularisoside D_4	$C_{55}H_{83}O_{33}S_3Na_3$	6.8	455.7998	$[M-3Na]^{3-}$	455.8015	-3.6	*Colochirus quadrangularis* (Cucumariidae, Dendrochirotida)	10.3390/md18080394
57	Quadrangularisoside E	$C_{54}H_{81}O_{31}S_3Na_3$	10.3	440.4647	$[M-3Na]^{3-}$	440.4631	3.6	*Colochirus quadrangularis* (Cucumariidae, Dendrochirotida)	10.3390/md18080394
58	Chilensoside A	$C_{60}H_{92}O_{34}S_2Na_2$	5.7	710.2461	$[M-2Na]^{2-}$	710.2444	2.4	*Paracaudina chilensis* (Caudinidae, Molpadida)	10.3390/molecules27217655
59	Chilensoside A_1	$C_{60}H_{92}O_{33}S_2Na_2$	10.9	702.2487	$[M-2Na]^{2-}$	702.2487	-0.1	*Paracaudina chilensis* (Caudinidae, Molpadida)	10.3390/molecules27217655
60	Chilensoside B	$C_{60}H_{92}O_{33}S_2Na_2$	10.8	702.2487	$[M-2Na]^{2-}$	702.2477	1.4	*Paracaudina chilensis* (Caudinidae, Molpadida)	10.3390/molecules27217655
61	Chilensoside C	$C_{60}H_{91}O_{36}S_3Na_3$	9.8	494.4823	$[M-3Na]^{3-}$	494.4841	-3.7	*Paracaudina chilensis* (Caudinidae, Molpadida)	10.3390/molecules27217655
62	Chilensoside D	$C_{60}H_{90}O_{39}S_4Na_4$	- [a]	390.5991	$[M-4Na]^{4-}$	390.6005	-3.6	*Paracaudina chilensis* (Caudinidae, Molpadida)	10.3390/molecules27217655
63	Chilensoside E	$C_{60}H_{90}O_{39}S_4Na_4$	- [a]	390.5991	$[M-4Na]^{4-}$	390.5999	-2.0	*Paracaudina chilensis* (Caudinidae, Molpadida)	10.3390/md21020114
64	Chilensoside F	$C_{60}H_{90}O_{39}S_4Na_4$	- [a]	390.5991	$[M-4Na]^{4-}$	390.5998	-1.8	*Paracaudina chilensis* (Caudinidae, Molpadida)	10.3390/md21020114
65	Chilensoside G	$C_{66}H_{100}O_{44}S_4Na_4$	- [a]	431.1123	$[M-4Na]^{4-}$	431.1139	-3.7	*Paracaudina chilensis* (Caudinidae, Molpadida)	10.3390/md21020114
66	Chitonoidoside A	$C_{53}H_{83}O_{23}SNa$	16.3	1119.5051	$[M-Na]^{-}$	1119.5062	-1.0	*Psolus chitonoides* (Psolidae, Dendrochirotida)	10.3390/md19080449
67	Chitonoidoside A_1	$C_{53}H_{81}O_{24}SNa$	14.8	1133.4844	$[M-Na]^{-}$	1133.4828	1.4	*Psolus chitonoides* (Psolidae, Dendrochirotida)	10.3390/md19080449
68	Chitonoidoside B	$C_{65}H_{103}O_{32}SNa$	13.4	1427.6159	$[M-Na]^{-}$	1427.6130	2.0	*Psolus chitonoides* (Psolidae, Dendrochirotida)	10.3390/md19080449
69	Chitonoidoside C	$C_{53}H_{80}O_{27}S_2Na_2$	12.8	606.2170	$[M-2Na]^{2-}$	606.2160	1.6	*Psolus chitonoides* (Psolidae, Dendrochirotida)	10.3390/md19080449
70	Chitonoidoside D	$C_{59}H_{90}O_{32}S_2Na_2$	10.7	687.2434	$[M-2Na]^{2-}$	687.2421	1.9	*Psolus chitonoides* (Psolidae, Dendrochirotida)	10.3390/md19080449
71	Chitonoidoside E	$C_{65}H_{102}O_{35}S_2Na_2$	11.5	753.2827	$[M-2Na]^{2-}$	753.2836	-1.2	*Psolus chitonoides* (Psolidae, Dendrochirotida)	10.3390/md19080449
72	Chitonoidoside E_1	$C_{65}H_{100}O_{36}S_2Na_2$	10.9	760.2723	$[M-2Na]^{2-}$	760.2716	1.0	*Psolus chitonoides* (Psolidae, Dendrochirotida)	10.3390/md19120696
73	Chitonoidoside F	$C_{54}H_{82}O_{28}S_2Na_2$	12.7	621.2223	$[M-2Na]^{2-}$	621.2223	-0.1	*Psolus chitonoides* (Psolidae, Dendrochirotida)	10.3390/md19120696
74	Chitonoidoside G	$C_{66}H_{104}O_{36}S_2Na_2$	11.2	768.2880	$[M-2Na]^{2-}$	768.2867	1.7	*Psolus chitonoides* (Psolidae, Dendrochirotida)	10.3390/md19120696
75	Chitonoidoside H	$C_{59}H_{90}O_{32}S_2Na_2$	11.0	687.2434	$[M-2Na]^{2-}$	687.2427	1.0	*Psolus chitonoides* (Psolidae, Dendrochirotida)	10.3390/md19120696
76	Chitonoidoside I	$C_{65}H_{100}O_{37}S_2Na_2$	10.1	768.2698	$[M-2Na]^{2-}$	768.2668	3.9	*Psolus chitonoides* (Psolidae, Dendrochirotida)	10.3390/md20060369
77	Chitonoidoside J	$C_{66}H_{101}O_{40}S_3Na_3$	9.3	543.1683	$[M-3Na]^{3-}$	543.1681	0.3	*Psolus chitonoides* (Psolidae, Dendrochirotida)	10.3390/md20060369
78	Chitonoidoside K	$C_{67}H_{104}O_{43}S_4Na_4$	- [a]	431.1214	$[M-4Na]^{4-}$	431.1223	-2.1	*Psolus chitonoides* (Psolidae, Dendrochirotida)	10.3390/md20060369
79	Chitonoidoside K_1	$C_{67}H_{104}O_{44}S_4Na_4$	- [a]	435.1201	$[M-4Na]^{4-}$	435.1200	0.3	*Psolus chitonoides* (Psolidae, Dendrochirotida)	10.3390/md20060369
80	Chitonoidoside L	$C_{67}H_{102}O_{44}S_4Na_4$	- [a]	434.6162	$[M-4Na]^{4-}$	434.6182	-4.6	*Psolus chitonoides* (Psolidae, Dendrochirotida)	10.3390/md20060369
81	Magnumoside A_3	$C_{41}H_{63}O_{15}SNa$	12.8	827.3893	$[M-Na]^{-}$	827.3888	0.6	*Neothynidium* (=*Massinium*) *magnum* (Phyllophoridae, Dendrochirotida)	10.3390/md15080256

82	Magnumoside A_4	$C_{41}H_{63}O_{15}SNa$	13.1	827.3893	$[M-Na]^-$	827.3870	2.8	*Neothynidium* (=*Massinium*) *magnum* (Phyllophoridae, Dendrochirotida)	10.3390/md15080256
83	Magnumoside B_3	$C_{53}H_{83}O_{24}SNa$	10.6	1135.5000	$[M-Na]^-$	1135.4991	0.8	*Neothynidium* (=*Massinium*) *magnum* (Phyllophoridae, Dendrochirotida)	10.1177/1934578x1701201013
84	Magnumoside C_1	$C_{53}H_{82}O_{28}S_2Na_2$	5.2	615.2223	$[M-2Na]^{2-}$	615.2216	1.1	*Neothynidium* (=*Massinium*) *magnum* (Phyllophoridae, Dendrochirotida)	10.3390/md15080256
85	Magnumoside C_2	$C_{53}H_{82}O_{28}S_2Na_2$	5.4	615.2223	$[M-2Na]^{2-}$	615.2200	3.7	*Neothynidium* (=*Massinium*) *magnum* (Phyllophoridae, Dendrochirotida)	10.3390/md15080256
86	Magnumoside C_3	$C_{53}H_{82}O_{27}S_2Na_2$	9.1	607.2248	$[M-2Na]^{2-}$	607.2250	-0.3	*Neothynidium* (=*Massinium*) *magnum* (Phyllophoridae, Dendrochirotida)	10.1177/1934578x1701201013
87	Magnumoside C_4	$C_{53}H_{82}O_{27}S_2Na_2$	9.2	607.2248	$[M-2Na]^{2-}$	607.2236	2.0	*Neothynidium* (=*Massinium*) *magnum* (Phyllophoridae, Dendrochirotida)	10.3390/md15080256
88	Colochiroside A_1	$C_{55}H_{85}O_{26}SNa$	13.2	1193.5055	$[M-Na]^-$	1193.5060	-0.4	*Colochirus robustus* (Cucumariidae, Dendrochirotida)	10.1177/1934578x1601100316
89	Colochiroside A_2	$C_{53}H_{83}O_{24}SNa$	15.0	1135.5000	$[M-Na]^-$	1135.4973	2.4	*Colochirus robustus* (Cucumariidae, Dendrochirotida)	10.1177/1934578x1601100316
90	Colochiroside A_3	$C_{53}H_{83}O_{24}SNa$	15.2	1135.5000	$[M-Na]^-$	1135.4983	1.5	*Colochirus robustus* (Cucumariidae, Dendrochirotida)	10.1177/1934578x1601100316
91	Colochiroside B_1	$C_{55}H_{85}O_{26}SNa$	8.1	1193.5055	$[M-Na]^-$	1193.5031	2.0	*Colochirus robustus* (Cucumariidae, Dendrochirotida)	10.1177/1934578X1501001014
92	Colochiroside B_2	$C_{55}H_{85}O_{26}SNa$	8.3	1193.5055	$[M-Na]^-$	1193.5049	0.5	*Colochirus robustus* (Cucumariidae, Dendrochirotida)	10.1177/1934578X1501001014
93	Colochiroside B_3	$C_{55}H_{83}O_{26}SNa$	10.4	1191.4899	$[M-Na]^-$	1191.4878	1.7	*Colochirus robustus* (Cucumariidae, Dendrochirotida)	10.1177/1934578X1501001014
94	Colochiroside C	$C_{53}H_{80}O_{27}S_2Na_2$	12.2	606.2170	$[M-2Na]^{2-}$	606.2165	0.8	*Colochirus robustus* (Cucumariidae, Dendrochirotida)	10.1177/1934578X1501001014
95	Colochiroside D	$C_{54}H_{83}O_{26}SNa$	13.2	1179.4899	$[M-Na]^-$	1179.4888	0.9	*Colochirus robustus* (Cucumariidae, Dendrochirotida)	10.1177/1934578x1601100316
96	Colochiroside E	$C_{49}H_{75}O_{22}SNa$	12.5	1047.4476	$[M-Na]^-$	1047.4462	1.4	*Colochirus robustus* (Cucumariidae, Dendrochirotida)	10.1177/1934578x1601100611
97	Neothyonidioside	$C_{53}H_{81}O_{24}SNa$	13.9	1133.4844	$[M-Na]^-$	1133.4850	-0.5	*Colochirus robustus* (Cucumariidae, Dendrochirotida)	10.1177/1934578X1501001014
98	Lefevreoside B	$C_{55}H_{85}O_{25}SNa$	14.5	1177.5106	$[M-Na]^-$	1177.5131	-2.1	*Colochirus robustus* (Cucumariidae, Dendrochirotida)	10.1177/1934578X1501001014
99	Lefevreoside C	$C_{55}H_{85}O_{25}SNa$	14.2	1177.5106	$[M-Na]^-$	1177.5130	-2.0	*Colochirus robustus* (Cucumariidae, Dendrochirotida)	10.1177/1934578X1501001014
100	Hemoiedemoside B	$C_{54}H_{81}O_{31}S_3Na_3$	10.6	440.4647	$[M-3Na]^{3-}$	440.4646	0.2	*Colochirus robustus* (Cucumariidae, Dendrochirotida)	10.1177/1934578X1501001014
101	Typicoside A_1	$C_{55}H_{83}O_{25}SNa$	13.7	1175.4950	$[M-Na]^-$	1175.4897	4.5	*Actinocucumis typica* (Cucumariidae, Dendrochirotida)	10.1177/1934578X1300800307
102	Typicoside A_2	$C_{55}H_{85}O_{25}SNa$	14.4	1177.5106	$[M-Na]^-$	1177.5100	0.5	*Actinocucumis typica* (Cucumariidae, Dendrochirotida)	10.1177/1934578X1300800307
103	Typicoside B_1	$C_{56}H_{87}O_{26}SNa$	13.9	1207.5212	$[M-Na]^-$	1207.5209	0.2	*Actinocucumis typica* (Cucumariidae, Dendrochirotida)	10.1177/1934578X1300800307
104	Typicoside C_1	$C_{54}H_{84}O_{28}S_2Na_2$	8.9	622.2301	$[M-2Na]^{2-}$	622.2300	0.1	*Actinocucumis typica* (Cucumariidae, Dendrochirotida)	10.1177/1934578X1300800307
105	Typicoside C_2	$C_{56}H_{86}O_{29}S_2Na_2$	12.0	643.2354	$[M-2Na]^{2-}$	643.2357	-0.5	*Actinocucumis typica* (Cucumariidae, Dendrochirotida)	10.1177/1934578X1300800307
106	Fallaxoside C_1	$C_{53}H_{80}O_{32}S_2Na_2$	4.8	646.2043	$[M-2Na]^{2-}$	646.2038	0.7	*Cucumaria fallax* (Cucumariidae, Dendrochirotida)	10.1177/1934578X1601100718
107	Fallaxoside C_2	$C_{53}H_{82}O_{31}S_2Na_2$	5.2	639.2146	$[M-2Na]^{2-}$	639.2141	0.8	*Cucumaria fallax* (Cucumariidae, Dendrochirotida)	10.1177/1934578X1601100718

108	Fallaxoside D_1	$C_{53}H_{79}O_{35}S_3Na_3$	4.3	457.1193	$[M-3Na]^{3-}$	457.1208	-3.2	*Cucumaria fallax* (Cucumariidae, Dendrochirotida)	10.1177/1934578X1601100718
109	Fallaxoside D_2	$C_{53}H_{81}O_{34}S_3Na_3$	4.7	452.4596	$[M-3Na]^{3-}$	452.4583	2.9	*Cucumaria fallax* (Cucumariidae, Dendrochirotida)	10.1177/1934578X1601100718
110	Fallaxoside D_6	$C_{59}H_{93}O_{35}S_3Na_3$	6.9	485.8225	$[M-3Na]^{3-}$	485.8241	-3.2	*Cucumaria fallax* (Cucumariidae, Dendrochirotida)	10.3390/molecules21070939
111	Fallaxoside D_7	$C_{53}H_{83}O_{34}S_3Na_3$	4.6	453.1315	$[M-3Na]^{3-}$	453.1328	-2.9	*Cucumaria fallax* (Cucumariidae, Dendrochirotida)	10.3390/molecules21070939
112	Violaceuside A	$C_{55}H_{85}O_{25}SNa$	14.6	1177.5106	$[M-Na]^{-}$	1177.5100	0.5	*Pseudocolochirus violaceus* (Cucumariidae, Dendrochirotida)	10.1177/1934578X1400900329
113	Violaceusoside C	$C_{53}H_{81}O_{24}SNa$	14.3	1133.4844	$[M-Na]^{-}$	1133.4825	1.7	*Pseudocolochirus violaceus* (Cucumariidae, Dendrochirotida)	10.1177/1934578X1400900329
114	Violaceusoside D	$C_{56}H_{86}O_{29}S_2Na_2$	12.4	643.2354	$[M-2Na]^{2-}$	643.2335	2.9	*Pseudocolochirus violaceus* (Cucumariidae, Dendrochirotida)	10.1177/1934578X1400900329
115	Violaceusoside E	$C_{53}H_{80}O_{27}S_2Na_2$	12.5	606.2170	$[M-2Na]^{2-}$	606.2164	0.9	*Pseudocolochirus violaceus* (Cucumariidae, Dendrochirotida)	10.1177/1934578X1400900329
116	Violaceuside II	$C_{53}H_{80}O_{27}S_2Na_2$	12.3	606.2170	$[M-2Na]^{2-}$	606.2170	-0.1	*Pseudocolochirus violaceus* (Cucumariidae, Dendrochirotida)	10.1177/1934578X1400900329
117	Holothurinoside A	$C_{60}H_{96}O_{29}$	9.8	1279.5965	$[M-Na]^{-}$	1279.5946	1.4	*Pseudocolochirus violaceus* (Cucumariidae, Dendrochirotida)	10.1177/1934578X1400900329
118	Philinopside E	$C_{53}H_{81}O_{24}SNa$	14.1	1133.4844	$[M-Na]^{-}$	1133.4835	0.8	*Pseudocolochirus violaceus* (Cucumariidae, Dendrochirotida)	10.1177/1934578X1400900329
119	Liouvilloside A	$C_{56}H_{85}O_{32}S_3Na_3$	10.7	455.1401	$[M-3Na]^{3-}$	455.1408	-1.6	*Pseudocolochirus violaceus* (Cucumariidae, Dendrochirotida)	10.1177/1934578X1400900329
120	Cladoloside A_2	$C_{57}H_{88}O_{24}$	15.7	1155.5593	$[M-H]^{-}$	1155.5581	1.0	*Cladolabes schmeltzii* (Sclerodactylidae, Dendrochirotida)	10.1177/1934578X1400901006
121	Cladoloside B	$C_{59}H_{92}O_{26}$	13.4	1215.5804	$[M-H]^{-}$	1215.5789	1.2	*Cladolabes schmeltzii* (Sclerodactylidae, Dendrochirotida)	10.1177/1934578X1300801107
122	Cladoloside B_1	$C_{63}H_{100}O_{29}$	13.9	1319.6278	$[M-H]^{-}$	1319.6344	-5.0	*Cladolabes schmeltzii* (Sclerodactylidae, Dendrochirotida)	10.1177/1934578X1300801107
123	Cladoloside B_2	$C_{63}H_{98}O_{29}$	12.7	1317.6121	$[M-H]^{-}$	1317.6111	0.8	*Cladolabes schmeltzii* (Sclerodactylidae, Dendrochirotida)	10.1177/1934578X1300801107
124	Cladoloside C	$C_{70}H_{110}O_{34}$	12.5	1493.6806	$[M-H]^{-}$	1493.6813	-0.5	*Cladolabes schmeltzii* (Sclerodactylidae, Dendrochirotida)	10.1177/1934578X1300801107
125	Cladoloside C_1	$C_{70}H_{112}O_{34}$	13.6	1495.6962	$[M-H]^{-}$	1495.7019	-3.8	*Cladolabes schmeltzii* (Sclerodactylidae, Dendrochirotida)	10.1177/1934578X1300801107
126	Cladoloside C_2	$C_{66}H_{106}O_{31}$	14.8	1393.6645	$[M-H]^{-}$	1393.6681	-2.6	*Cladolabes schmeltzii* (Sclerodactylidae, Dendrochirotida)	10.1177/1934578X1300801107
127	Cladoloside D	$C_{68}H_{106}O_{33}$	12.2	1449.6544	$[M-H]^{-}$	1449.6589	-3.1	*Cladolabes schmeltzii* (Sclerodactylidae, Dendrochirotida)	10.1177/1934578X1300801107
128	Cladoloside D_1	$C_{68}H_{108}O_{33}$	13.3	1451.6700	$[M-H]^{-}$	1451.6737	-2.5	*Cladolabes schmeltzii* (Sclerodactylidae, Dendrochirotida)	10.1016/j.carres.2018.08.003
129	Cladoloside D_2	$C_{66}H_{104}O_{32}$	8.8	1407.6438	$[M-H]^{-}$	1407.6470	-2.3	*Cladolabes schmeltzii* (Sclerodactylidae, Dendrochirotida)	10.1016/j.carres.2018.08.003
130	Cladoloside E_1	$C_{62}H_{98}O_{28}$	14.5	1289.6172	$[M-H]^{-}$	1289.6149	1.8	*Cladolabes schmeltzii* (Sclerodactylidae, Dendrochirotida)	10.1016/j.carres.2015.06.005
131	Cladoloside E_2	$C_{62}H_{96}O_{28}$	13.3	1287.6015	$[M-H]^{-}$	1287.5976	3.1	*Cladolabes schmeltzii* (Sclerodactylidae, Dendrochirotida)	10.1016/j.carres.2015.06.005
132	Cladoloside F_1	$C_{63}H_{100}O_{28}$	15.1	1303.6328	$[M-H]^{-}$	1303.6296	2.5	*Cladolabes schmeltzii* (Sclerodactylidae, Dendrochirotida)	10.1016/j.carres.2015.06.005
133	Cladoloside F_2	$C_{63}H_{98}O_{28}$	13.9	1301.6172	$[M-H]^{-}$	1301.6181	-0.7	*Cladolabes schmeltzii* (Sclerodactylidae, Dendrochirotida)	10.1016/j.carres.2015.06.005
134	Cladoloside G	$C_{69}H_{108}O_{33}$	13.0	1463.6700	$[M-H]^{-}$	1463.6735	-2.4	*Cladolabes schmeltzii* (Sclerodactylidae, Dendrochirotida)	10.1016/j.carres.2015.06.005
135	Cladoloside H_1	$C_{70}H_{112}O_{33}$	14.7	1479.7013	$[M-H]^{-}$	1479.7033	-1.3	*Cladolabes schmeltzii* (Sclerodactylidae, Dendrochirotida)	10.1016/j.carres.2015.06.005
136	Cladoloside I_1	$C_{63}H_{99}O_{32}SNa$	12.9	1399.5846	$[M-Na]^{-}$	1399.5878	-2.3	*Cladolabes schmeltzii* (Sclerodactylidae, Dendrochirotida)	10.1016/j.carres.2017.04.016

137	Cladoloside I$_2$	$C_{63}H_{97}O_{32}SNa$	11.8	1397.5689	[M−Na]$^-$	1397.5643	3.3	*Cladolabes schmeltzii* (Sclerodactylidae, Dendrochirotida)	10.1016/j.carres.2017.04.016
138	Cladoloside J$_1$	$C_{59}H_{91}O_{29}SNa$	12.7	1295.5372	[M−Na]$^-$	1295.5389	-1.3	*Cladolabes schmeltzii* (Sclerodactylidae, Dendrochirotida)	10.1016/j.carres.2017.04.016
139	Cladoloside K$_1$	$C_{70}H_{111}O_{37}SNa$	12.7	1575.6530	[M−Na]$^-$	1575.6537	-0.4	*Cladolabes schmeltzii* (Sclerodactylidae, Dendrochirotida)	10.1016/j.carres.2017.04.016
140	Cladoloside K$_2$	$C_{68}H_{107}O_{36}SNa$	8.3	1531.6268	[M−Na]$^-$	1531.6284	-1.0	*Cladolabes schmeltzii* (Sclerodactylidae, Dendrochirotida)	10.1016/j.carres.2017.04.016
141	Cladoloside L$_1$	$C_{66}H_{103}O_{34}SNa$	12.3	1471.6057	[M−Na]$^-$	1471.6095	-2.6	*Cladolabes schmeltzii* (Sclerodactylidae, Dendrochirotida)	10.1016/j.carres.2017.04.016
142	Cladoloside M	$C_{71}H_{114}O_{35}$	13.3	1525.7068	[M−H]$^-$	1525.7092	-1.6	*Cladolabes schmeltzii* (Sclerodactylidae, Dendrochirotida)	10.1016/j.carres.2018.08.003
143	Cladoloside M$_1$	$C_{67}H_{106}O_{32}$	12.7	1421.6594	[M−H]$^-$	1421.6609	-1.0	*Cladolabes schmeltzii* (Sclerodactylidae, Dendrochirotida)	10.1016/j.carres.2018.08.003
144	Cladoloside M$_2$	$C_{71}H_{112}O_{35}$	12.1	1523.6911	[M−H]$^-$	1523.6951	-2.6	*Cladolabes schmeltzii* (Sclerodactylidae, Dendrochirotida)	10.1016/j.carres.2018.08.003
145	Cladoloside N	$C_{69}H_{108}O_{34}$	11.9	1479.6649	[M−H]$^-$	1479.6703	-3.6	*Cladolabes schmeltzii* (Sclerodactylidae, Dendrochirotida)	10.1016/j.carres.2018.08.003
146	Cladoloside O	$C_{64}H_{102}O_{30}$	13.5	1349.6383	[M−H]$^-$	1349.6420	-2.7	*Cladolabes schmeltzii* (Sclerodactylidae, Dendrochirotida)	10.1016/j.carres.2018.08.004
147	Cladoloside P	$C_{69}H_{108}O_{34}$	11.8	1479.6649	[M−H]$^-$	1479.6650	-0.1	*Cladolabes schmeltzii* (Sclerodactylidae, Dendrochirotida)	10.1016/j.carres.2018.08.004
148	Cladoloside P$_1$	$C_{69}H_{110}O_{34}$	13.0	1481.6806	[M−H]$^-$	1481.6842	-2.4	*Cladolabes schmeltzii* (Sclerodactylidae, Dendrochirotida)	10.1016/j.carres.2018.08.004
149	Cladoloside P$_2$	$C_{65}H_{102}O_{31}$	12.3	1377.6332	[M−H]$^-$	1377.6378	-3.3	*Cladolabes schmeltzii* (Sclerodactylidae, Dendrochirotida)	10.1016/j.carres.2018.08.004
150	Cladoloside P$_3$	$C_{65}H_{104}O_{31}$	14.0	1379.6489	[M−H]$^-$	1379.6525	-2.6	*Cladolabes schmeltzii* (Sclerodactylidae, Dendrochirotida)	10.1016/j.carres.2018.08.004
151	Cladoloside Q	$C_{69}H_{108}O_{34}$	11.6	1479.6649	[M−H]$^-$	1479.6693	-3.0	*Cladolabes schmeltzii* (Sclerodactylidae, Dendrochirotida)	10.1016/j.carres.2018.08.003
152	Cladoloside R	$C_{69}H_{108}O_{34}$	11.8	1479.6649	[M−H]$^-$	1479.6686	-2.5	*Cladolabes schmeltzii* (Sclerodactylidae, Dendrochirotida)	10.1016/j.carres.2018.08.004
153	Holotoxin A$_1$	$C_{66}H_{104}O_{31}$	13.1	1391.6489	[M−H]$^-$	1391.6507	-1.3	*Cladolabes schmeltzii* (Sclerodactylidae, Dendrochirotida)	10.1177/1934578X1300801107
154	Cucumarioside A$_1$	$C_{55}H_{86}O_{22}$	17.1	1097.5538	[M−H]$^-$	1097.5552	-1.3	*Eupentacta fraudatrix* (Sclerodactylidae, Dendrochirotida)	10.1177/1934578X1200700426
155	Cucumarioside A$_2$	$C_{57}H_{88}O_{24}$	13.9	1155.5593	[M−H]$^-$	1155.5560	2.8	*Eupentacta fraudatrix* (Sclerodactylidae, Dendrochirotida)	10.1177/1934578X1200700710
156	Cucumarioside A$_3$	$C_{59}H_{94}O_{23}$	18.9	1169.6113	[M−H]$^-$	1169.6103	0.9	*Eupentacta fraudatrix* (Sclerodactylidae, Dendrochirotida)	10.1177/1934578X1200700426
157	Cucumarioside A$_4$	$C_{57}H_{90}O_{23}$	14.9	1141.5800	[M−H]$^-$	1141.5798	0.2	*Eupentacta fraudatrix* (Sclerodactylidae, Dendrochirotida)	10.1177/1934578X1200700426
158	Cucumarioside A$_6$	$C_{55}H_{84}O_{22}$	16.3	1095.5381	[M−H]$^-$	1095.5384	-0.2	*Eupentacta fraudatrix* (Sclerodactylidae, Dendrochirotida)	10.1177/1934578X1200700426
159	Cucumarioside A$_7$	$C_{55}H_{86}O_{23}$	9.7	1113.5487	[M−H]$^-$	1113.5498	-1.0	*Eupentacta fraudatrix* Sclerodactylidae, Dendrochirotida)	10.1177/1934578X1200700710
160	Cucumarioside A$_9$	$C_{55}H_{90}O_{23}$	8.4	1117.5800	[M−H]$^-$	1117.5783	1.5	*Eupentacta fraudatrix* (Sclerodactylidae, Dendrochirotida)	10.1177/1934578X1200700710
161	Cucumarioside A$_{10}$	$C_{48}H_{74}O_{20}$	11.5	969.4701	[M−H]$^-$	969.4673	2.9	*Eupentacta fraudatrix* (Sclerodactylidae, Dendrochirotida)	10.1177/1934578X1200700710
162	Cucumarioside A$_{11}$	$C_{55}H_{86}O_{23}$	10.0	1113.5487	[M−H]$^-$	1113.5501	-1.2	*Eupentacta fraudatrix* (Sclerodactylidae, Dendrochirotida)	10.1177/1934578X1200700710
163	Cucumarioside A$_{12}$	$C_{54}H_{84}O_{23}$	9.9	1099.5331	[M−H]$^-$	1099.5288	3.9	*Eupentacta fraudatrix* (Sclerodactylidae, Dendrochirotida)	10.1177/1934578X1200700426
164	Cucumarioside A$_{13}$	$C_{55}H_{84}O_{23}$	12.3	1111.5331	[M−H]$^-$	1111.5334	-0.3	*Eupentacta fraudatrix* (Sclerodactylidae, Dendrochirotida)	10.1177/1934578X1200700710
165	Cucumarioside A$_{14}$	$C_{55}H_{88}O_{24}$	6.4	1131.5593	[M−H]$^-$	1131.5554	3.4	*Eupentacta fraudatrix* (Sclerodactylidae, Dendrochirotida)	10.1177/1934578X1200700710

166	Cucumarioside A_{15}	$C_{55}H_{88}O_{22}$	18.7	1099.5694	$[M-H]^-$	1099.5696	-0.1	*Eupentacta fraudatrix* (Sclerodactylidae, Dendrochirotida)	10.1177/1934578X1200700426
167	Cucumarioside D	$C_{61}H_{94}O_{27}$	14.2	1257.5910	$[M-H]^-$	1257.5956	-3.7	*Eupentacta fraudatrix* (Sclerodactylidae, Dendrochirotida)	10.1177/1934578X1801300207
168	Cucumarioside H_2	$C_{60}H_{93}O_{30}SNa$	7.7	1325.5478	$[M-Na]^-$	1325.5468	0.7	*Eupentacta fraudatrix* (Sclerodactylidae, Dendrochirotida)	10.1080/14786419.2011.602637
169	Cucumarioside H_3	$C_{53}H_{81}O_{27}SNa$	8.8	1181.4691	$[M-Na]^-$	1181.4690	0.1	*Eupentacta fraudatrix* (Sclerodactylidae, Dendrochirotida)	10.1080/14786419.2011.602637
170	Cucumarioside H_4	$C_{62}H_{97}O_{30}SNa$	11.7	1353.5791	$[M-Na]^-$	1353.5732	4.3	*Eupentacta fraudatrix* (Sclerodactylidae, Dendrochirotida)	10.1080/14786419.2011.602637
171	Cucumarioside H_5	$C_{60}H_{91}O_{29}SNa$	12.4	1307.5372	$[M-Na]^-$	1307.5371	0.1	*Eupentacta fraudatrix* (Sclerodactylidae, Dendrochirotida)	10.1080/14786419.2011.602637
172	Cucumarioside H_6	$C_{60}H_{93}O_{29}SNa$	13.6	1309.5529	$[M-Na]^-$	1309.5561	-2.5	*Eupentacta fraudatrix* (Sclerodactylidae, Dendrochirotida)	10.1177/1934578X1100600806
173	Cucumarioside H_7	$C_{60}H_{95}O_{29}SNa$	14.9	1311.5685	$[M-Na]^-$	1311.5688	-0.2	*Eupentacta fraudatrix* (Sclerodactylidae, Dendrochirotida)	10.1177/1934578X1100600806
174	Cucumarioside H_8	$C_{58}H_{89}O_{29}NaS$	6.3	1281.5216	$[M-Na]^-$	1281.5205	0.8	*Eupentacta fraudatrix* (Sclerodactylidae, Dendrochirotida)	10.1177/1934578X1100600806
175	Cucumarioside I_1	$C_{60}H_{92}O_{32}S_2Na_2$	11.9	694.2512	$[M-2Na]^{2-}$	694.2526	-2.0	*Eupentacta fraudatrix* (Sclerodactylidae, Dendrochirotida)	10.1177/1934578X1300800805
176	Cucumarioside I_2	$C_{60}H_{90}O_{32}S_2Na_2$	11.3	693.2434	$[M-2Na]^{2-}$	693.2432	0.3	*Eupentacta fraudatrix* (Sclerodactylidae, Dendrochirotida)	10.1080/14786419.2013.778851
177	Cucumarioside I_3	$C_{60}H_{92}O_{33}S_2Na_2$	6.7	702.2487	$[M-2Na]^{2-}$	702.2497	-1.5	*Eupentacta fraudatrix* (Sclerodactylidae, Dendrochirotida)	10.1177/1934578X1300800805
178	Cucumarioside I_4	$C_{53}H_{80}O_{30}S_2Na_2$	7.4	630.2093	$[M-2Na]^{2-}$	630.2117	-3.7	*Eupentacta fraudatrix* (Sclerodactylidae, Dendrochirotida)	10.1177/1934578X1300800805
179	Cucumarioside A_0-1	$C_{60}H_{93}O_{30}SNa$	11.2	1325.5478	$[M-Na]^-$	1325.5552	-5.6	*Cucumaria djakonovi* (Cucumariidae, Dendrochirotida)	- [b]
180	Frondoside D	$C_{60}H_{95}O_{30}SNa$	9.1	1327.5634	$[M-Na]^-$	1327.5691	-4.3	*Cucumaria djakonovi* (Cucumariidae, Dendrochirotida)	- [b]
181	Okhotoside A_1-1	$C_{55}H_{85}O_{26}SNa$	12.3	1193.5055	$[M-Na]^-$	1193.5105	-4.2	*Cucumaria djakonovi* (Cucumariidae, Dendrochirotida)	- [b]
182	Turquetoside A	$C_{54}H_{84}O_{27}S_2Na_2$	14.1	614.2326	$[M-2Na]^{2-}$	614.2348	-3.6	*Staurocucumis turqueti* (Cucumariidae, Dendrochirotida)	10.1016/j.bse.2013.08.012
183	Cucumarioside C_1	$C_{60}H_{92}O_{26}$	14.7	1227.5804	$[M-H]^-$	1227.5885	-6.6	*Solaster pacificus* (Solasteridae, Valvatida)	10.3390/biom11030427
184	Cucumarioside C_2	$C_{60}H_{92}O_{26}$	15.1	1227.5804	$[M-H]^-$	1227.5872	-5.5	*Solaster pacificus* (Solasteridae, Valvatida)	10.3390/biom11030427
185	Pacificusoside A	$C_{57}H_{86}O_{27}$	8.1	1201.5284	$[M-H]^-$	1201.5347	-5.3	*Solaster pacificus* (Solasteridae, Valvatida)	10.3390/biom11030427
186	Pacificusoside B	$C_{53}H_{82}O_{24}$	10.5	1101.5123	$[M-H]^-$	1101.5172	-4.4	*Solaster pacificus* (Solasteridae, Valvatida)	10.3390/biom11030427
187	Pacificusoside C	$C_{60}H_{94}O_{26}$	15.9	1229.5961	$[M-H]^-$	1229.6025	-5.2	*Solaster pacificus* (Solasteridae, Valvatida)	10.3390/biom11030427
188	Pacificusoside E	$C_{54}H_{82}O_{22}$	14.9	1081.5225	$[M-H]^-$	1081.5232	-0.6	*Solaster pacificus* (Solasteridae, Valvatida)	10.3390/md20030216
189	Pacificusoside G	$C_{54}H_{82}O_{22}$	14.5	1081.5225	$[M-H]^-$	1081.5251	-2.4	*Solaster pacificus* (Solasteridae, Valvatida)	10.3390/md20030216
190	Pacificusoside H	$C_{55}H_{83}O_{25}SNa$	14.5	1175.4950	$[M-Na]^-$	1175.4983	-2.8	*Solaster pacificus* (Solasteridae, Valvatida)	10.3390/md20030216
191	Pacificusoside J	$C_{54}H_{84}O_{25}$	10.1	1131.5229	$[M-H]^-$	1131.5243	-1.2	*Solaster pacificus* (Solasteridae, Valvatida)	10.3390/md20030216

[a] The data have been obtained by direct infusion-ESI MS; [b] Data not published

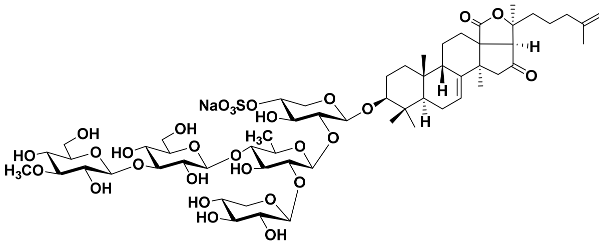

Cucumarioside A_2-2

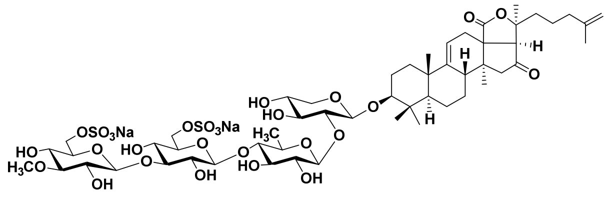

Psolusoside A

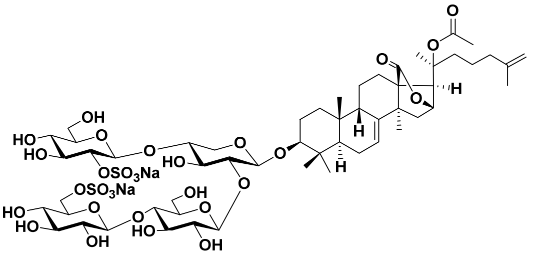

Psolusoside B

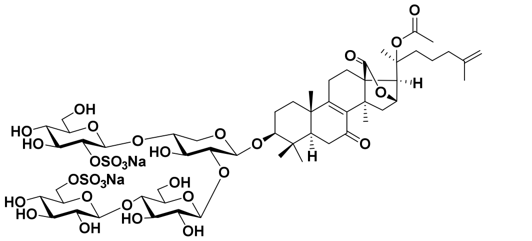

Psolusoside B_1

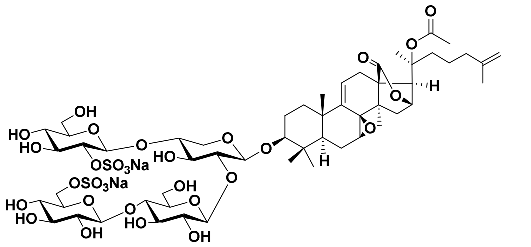

Psolusoside B_2

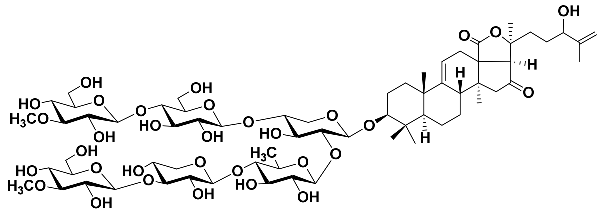

Psolusoside C_1

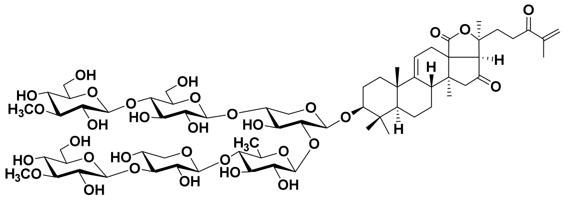

Psolusoside C_2

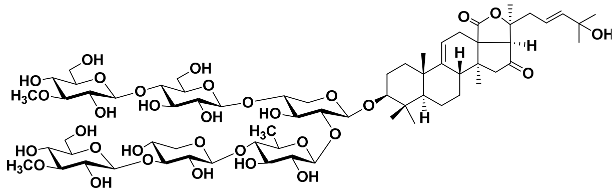

Psolusoside C_3

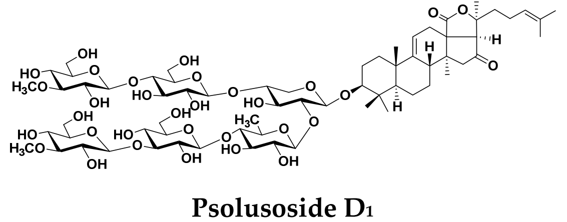

Psolusoside D_1

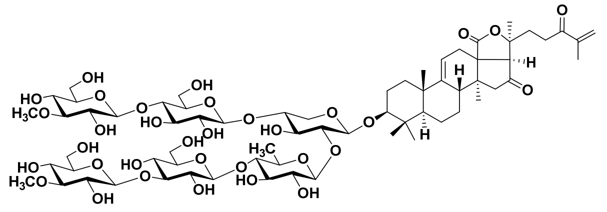

Psolusoside D_2

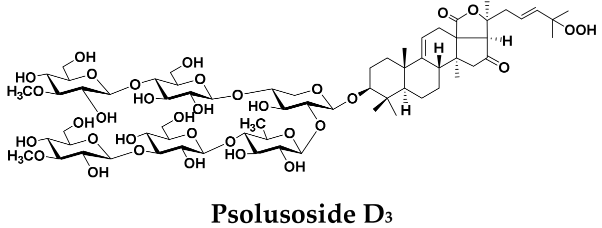

Psolusoside D_3

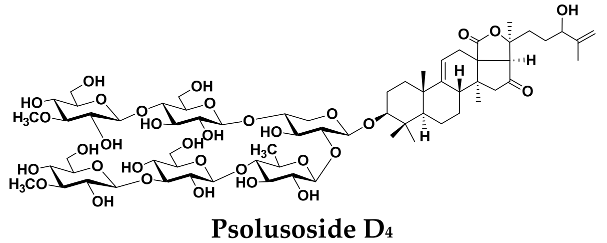

Psolusoside D_4

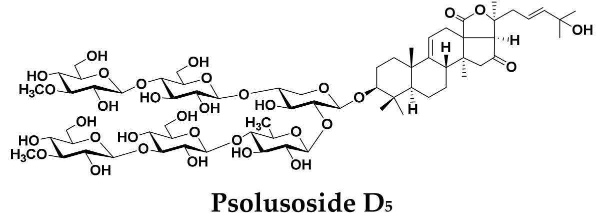

Psolusoside D_5

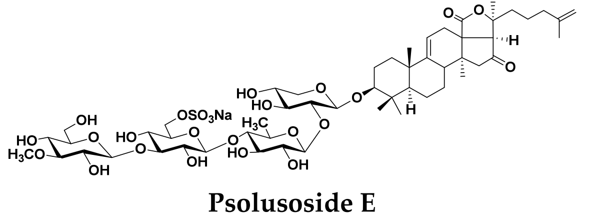

Psolusoside E

Figure S1. Structures of cucumarioside A_2-2, psolusosides B, B_1, B_2, C_1–C_3, D_1-D_5, and E.

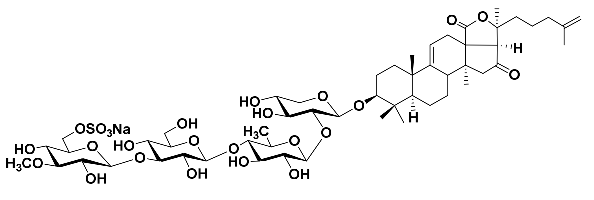

Psolusoside F

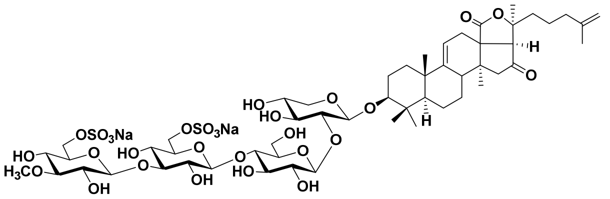

Psolusoside G

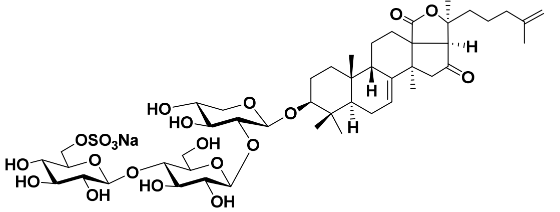

Psolusoside H

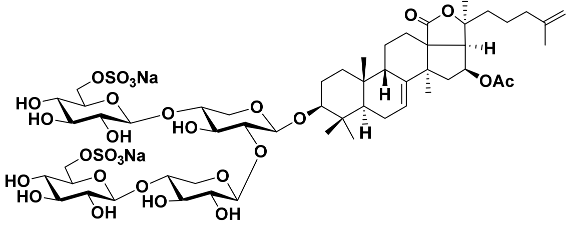

Psolusoside I

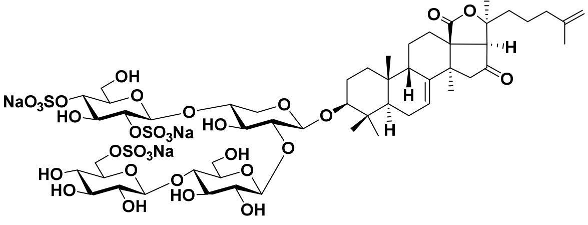

Psolusoside J

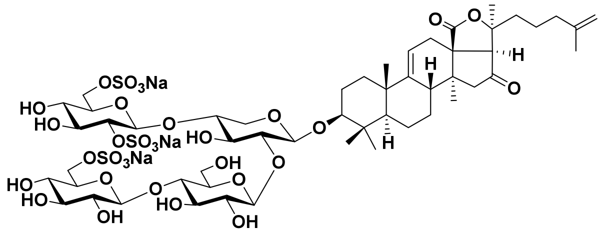

Psolusoside K

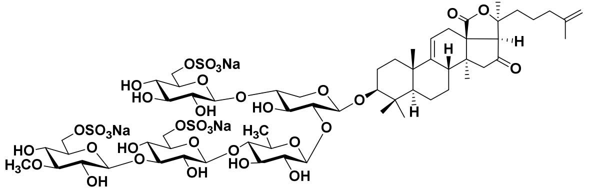

Psolusoside L

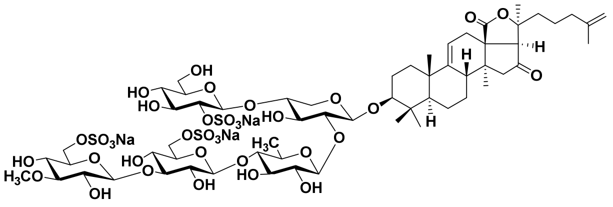

Psolusoside M

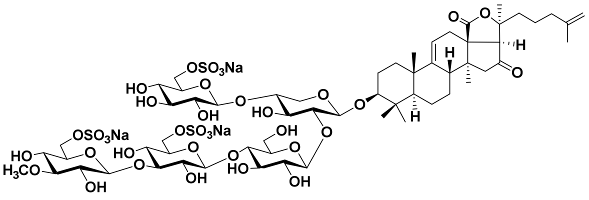

Psolusoside N

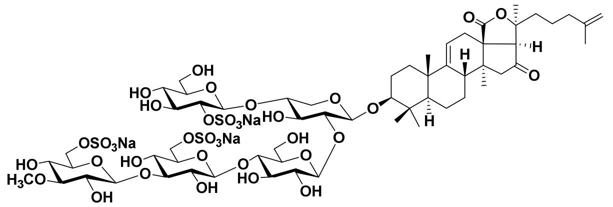

Psolusoside O

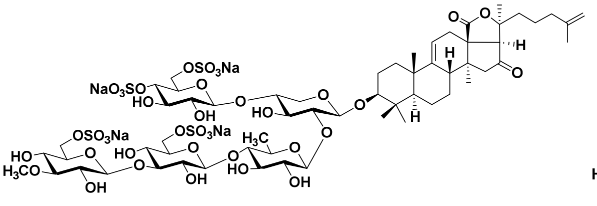

Psolusoside P

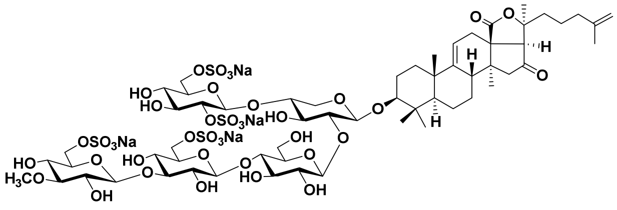

Psolusoside Q

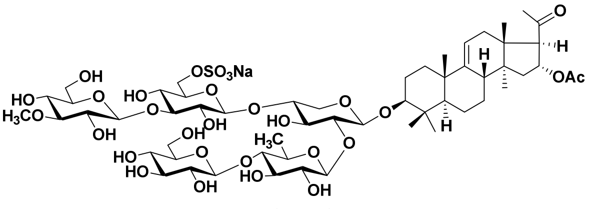

Kuriloside A

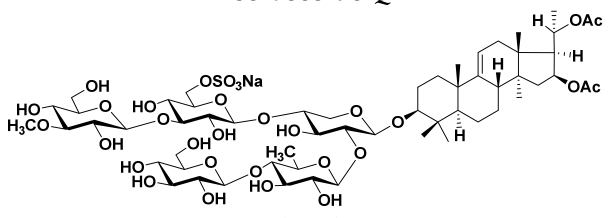

Kuriloside A_1

Figure S2. Structures of psolusosides F, G, H, I, J, K, L, M, N, O, P, and Q, kurilosides A and A_1.

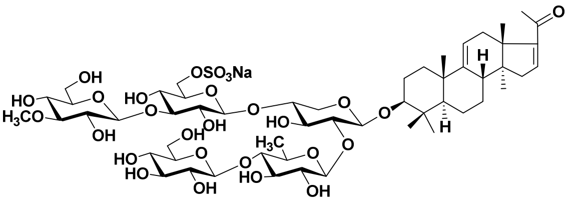

Kuriloside A$_2$

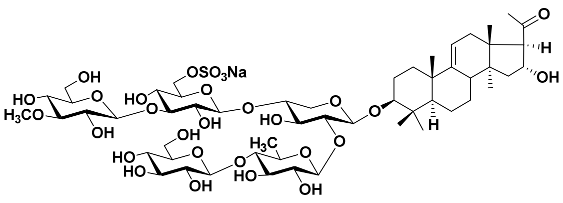

Kuriloside A$_3$

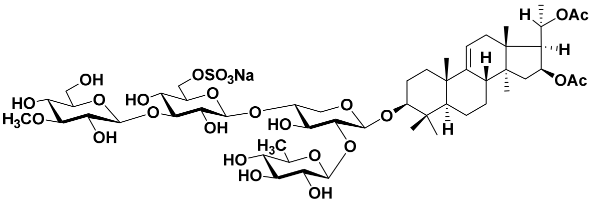

Kuriloside C$_1$

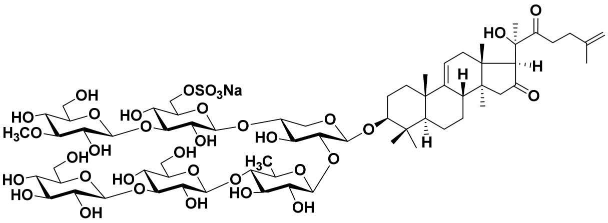

Kuriloside D

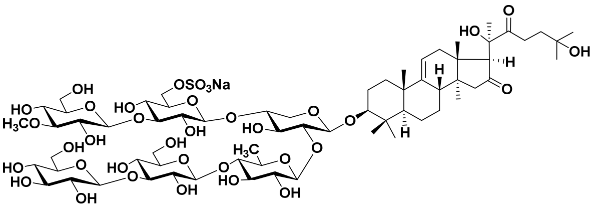

Kuriloside D$_1$

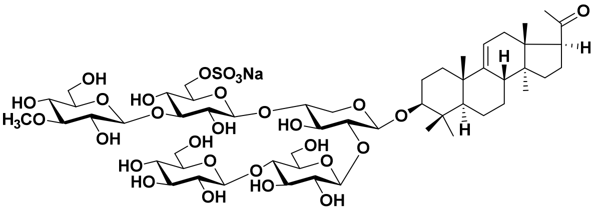

Kuriloside E

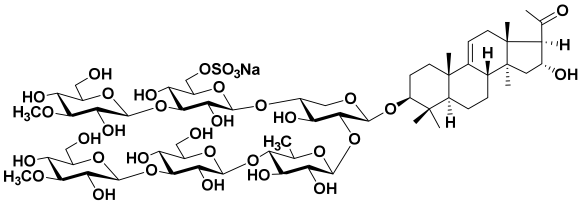

Kuriloside F

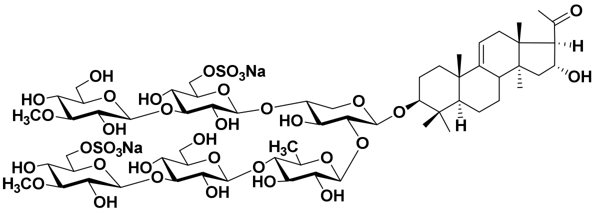

Kuriloside G

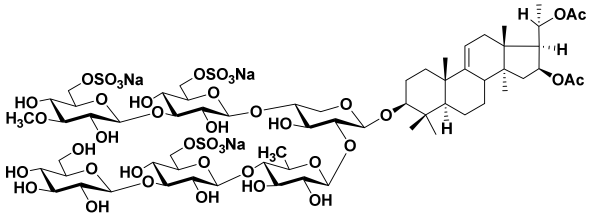

Kuriloside H

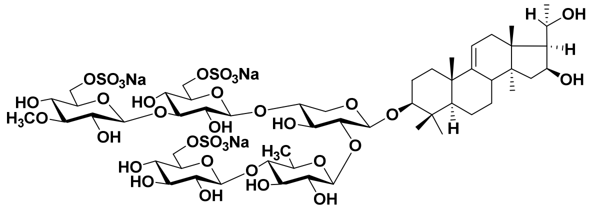

Kuriloside I

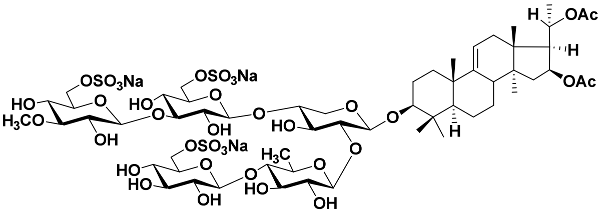

Kuriloside I$_1$

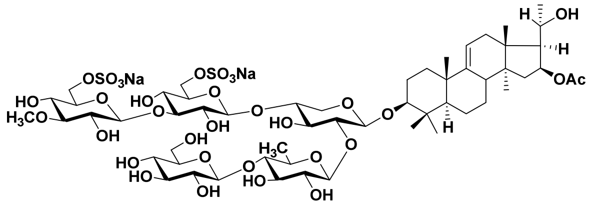

Kuriloside J

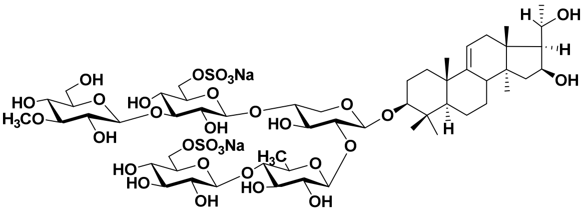

Kuriloside K

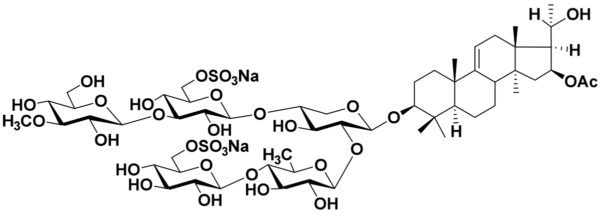

Kuriloside K$_1$

Figure S3. Structures of kurilosides A$_2$, A$_3$, C$_1$, D, D$_1$, E, F, G, H, I, I$_1$, J, K, and K$_1$.

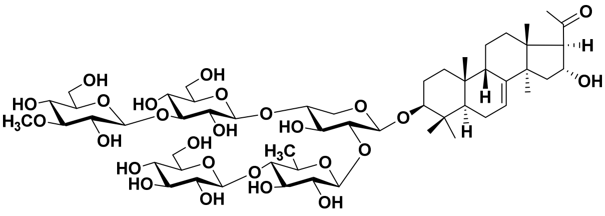

DS-Kuriloside M

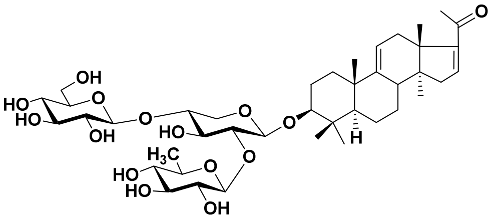

DS-Kuriloside L

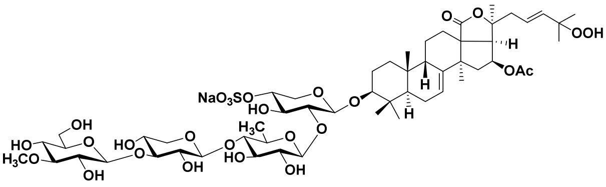

Quadrangularisoside A

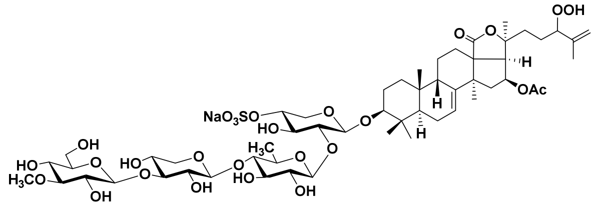

Quadrangularisoside A$_1$

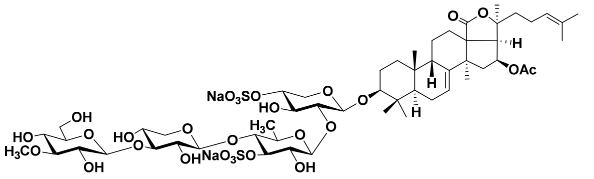

Quadrangularisoside B

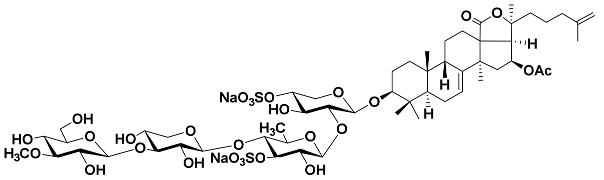

Quadrangularisoside B$_1$

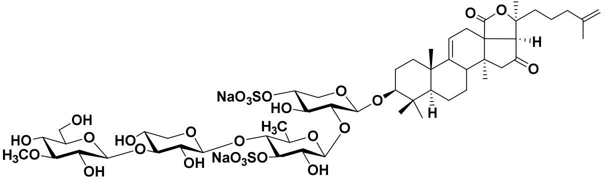

Quadrangularisoside B$_2$

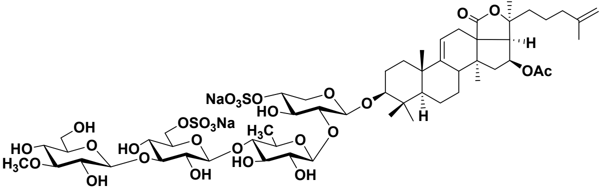

Quadrangularisoside C

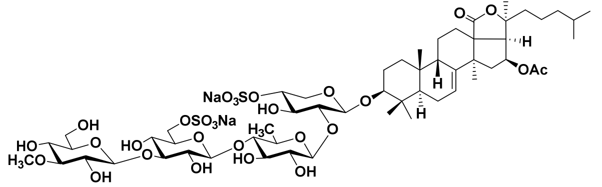

Quadrangularisoside C$_1$

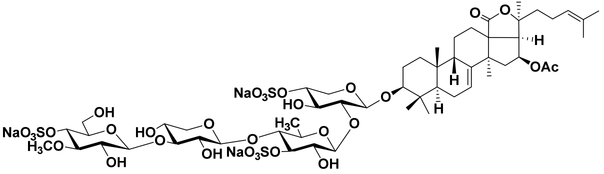

Quadrangularisoside D

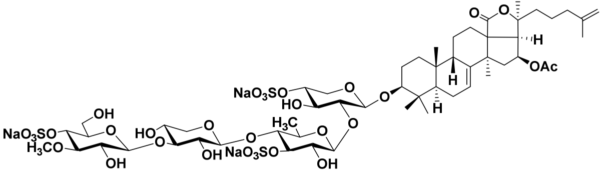

Quadrangularisoside D$_1$

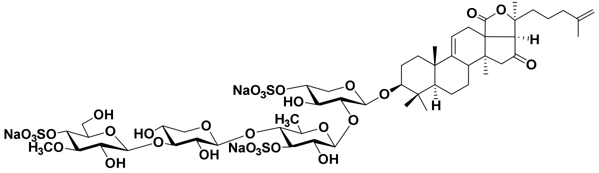

Quadrangularisoside D$_2$

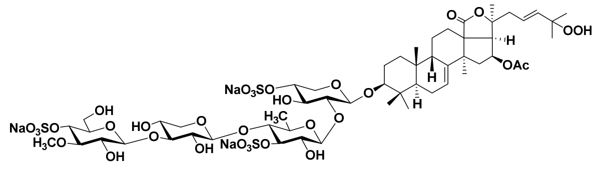

Quadrangularisoside D$_3$

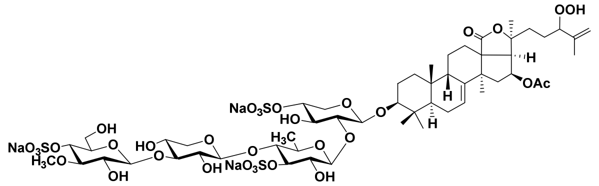

Quadrangularisoside D$_4$

Figure S4. Structures of DS-kurilosides L and M, quadrangularisosides A, A$_1$, B, B$_1$, B$_2$, C, C$_1$, D, and D$_1$–D$_4$.

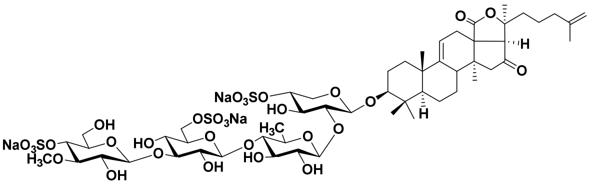

Quadrangularisoside E

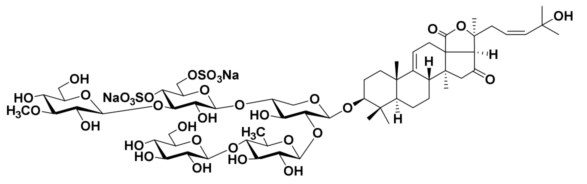

Chilensoside A

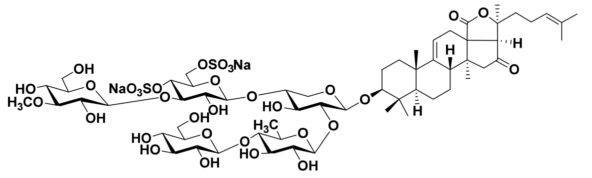

Chilensoside A1

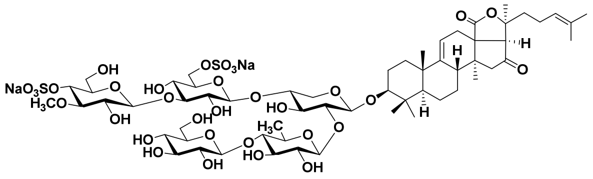

Chilensoside B

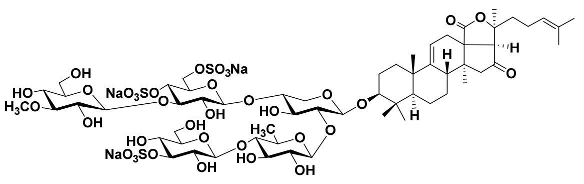

Chilensoside C

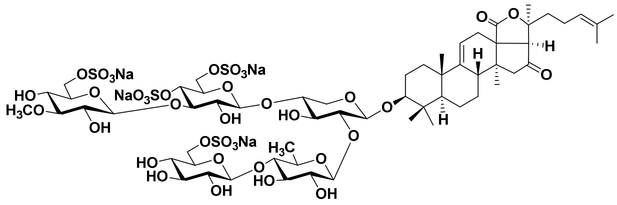

Chilensoside D

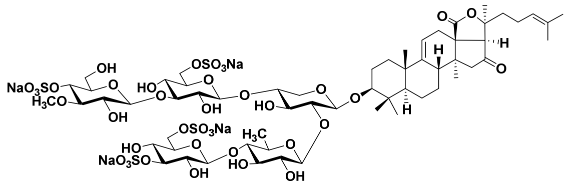

Chilensoside E

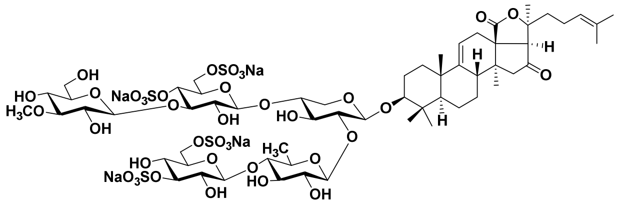

Chilensoside F

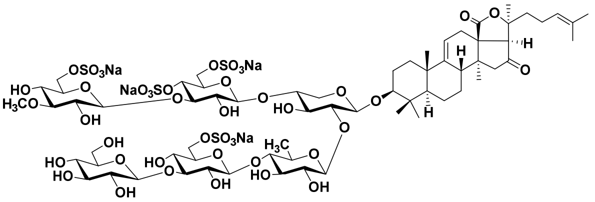

Chilensoside G

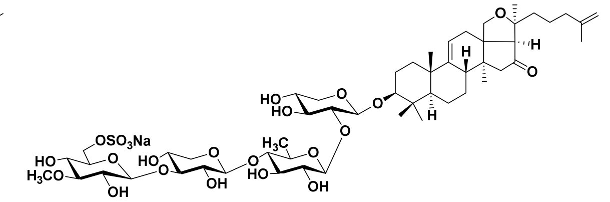

Chitonoidoside A

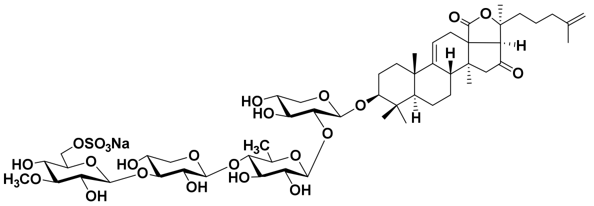

Chitonoidoside A1

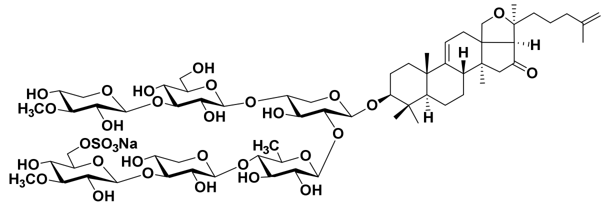

Chitonoidoside B

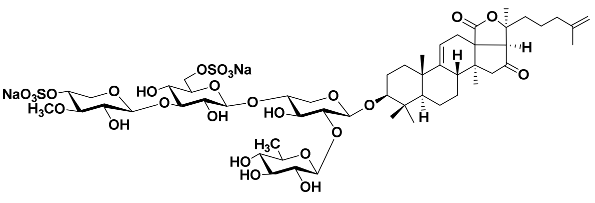

Chitonoidoside C

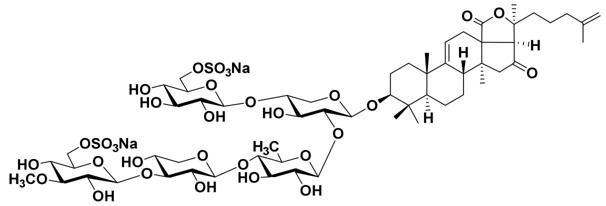

Chitonoidoside D

Figure S5. Structures of quadrangularisoside E, chilensosides A, A1, B, C, D, E, F, and G, and chitonoidosides A, A1, B, C, and D.

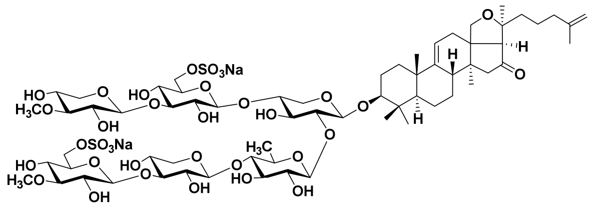

Chitonoidoside E

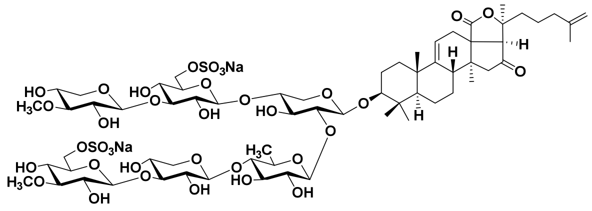

Chitonoidoside E1

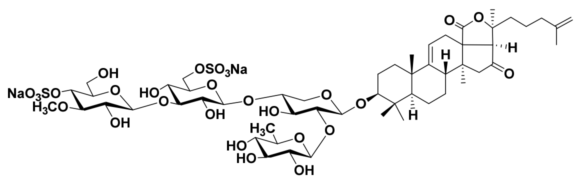

Chitonoidoside F

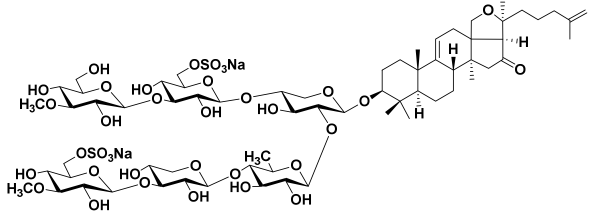

Chitonoidoside G

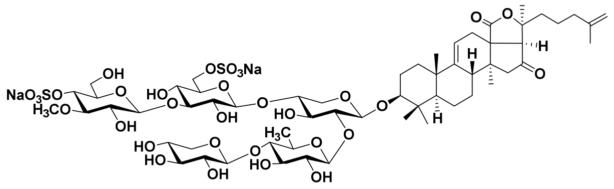

Chitonoidoside H

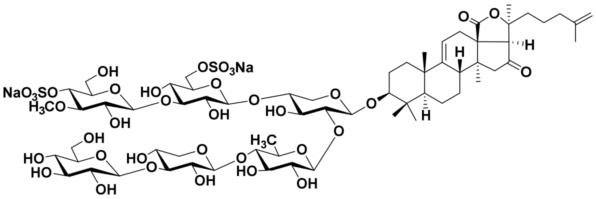

Chitonoidoside I

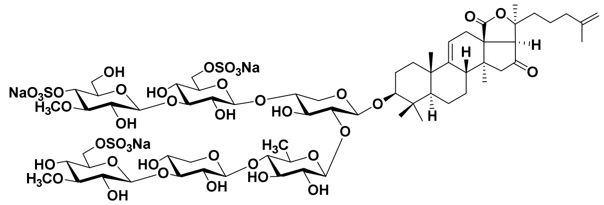

Chitonoidoside J

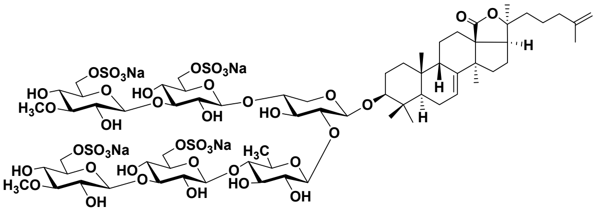

Chitonoidoside K

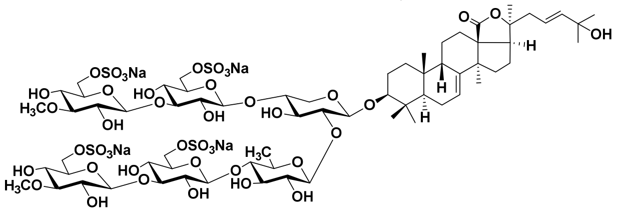

Chitonoidoside K1

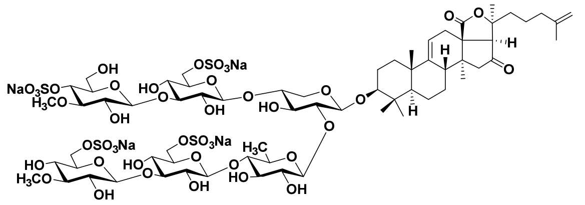

Chitonoidoside L

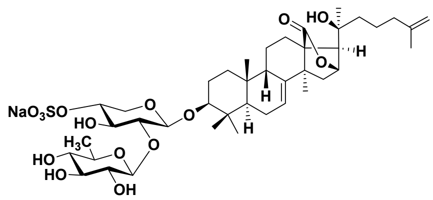

Magnumoside A3

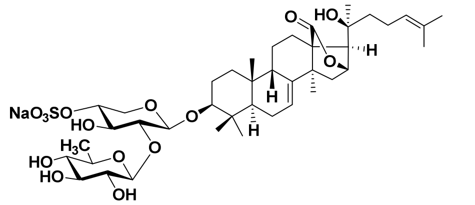

Magnumoside A4

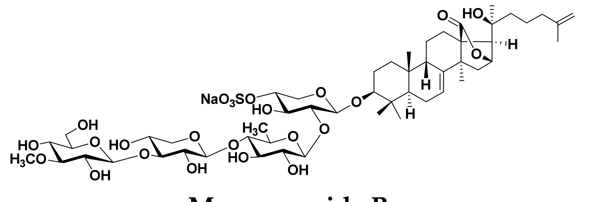

Magnumoside B3

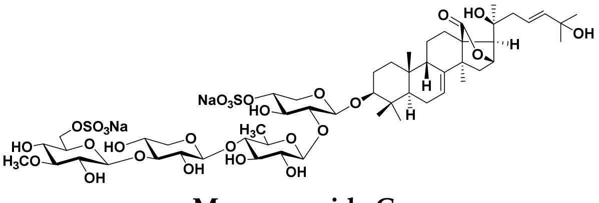

Magnumoside C1

Figure S6. Structures of chitonoidosides E, E1, F, G, H, I, J, K, K1, and L, magnumosides A3, A4, B3, and C1.

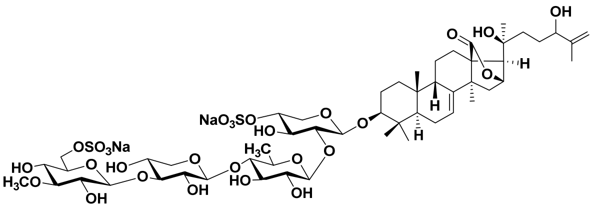

Magnumoside C2

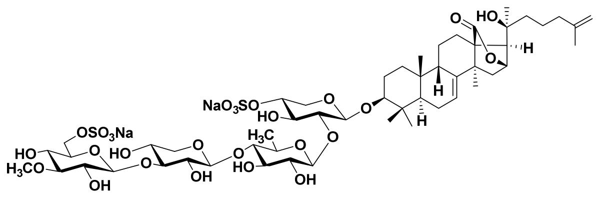

Magnumoside C3

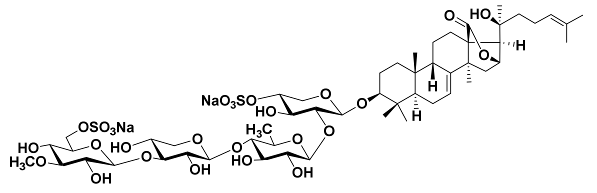

Magnumoside C4

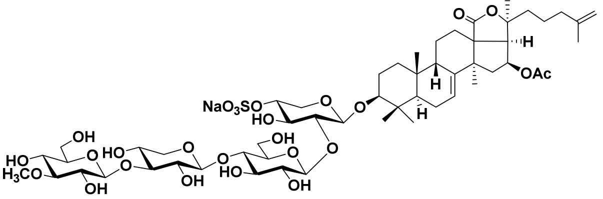

Colochiroside A1

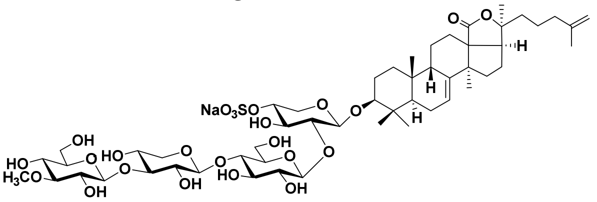

Colochiroside A2

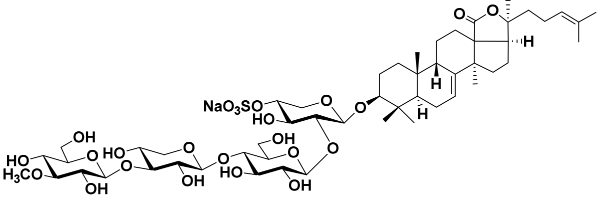

Colochiroside A3

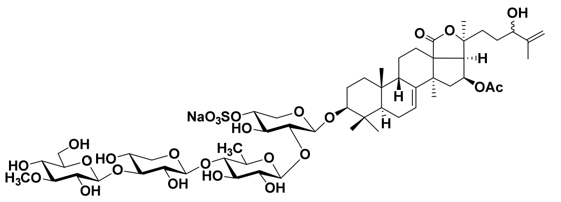

Colochiroside B1

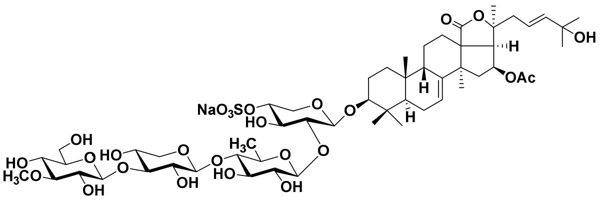

Colochiroside B2

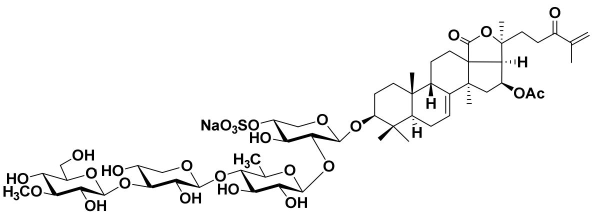

Colochiroside B3

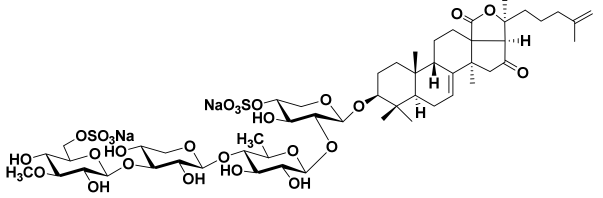

Colochiroside C

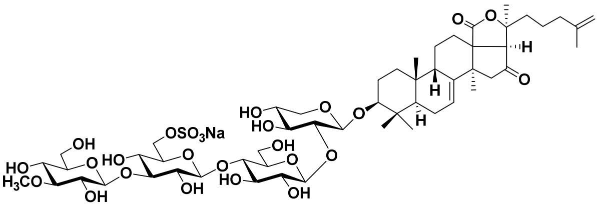

Colochiroside D

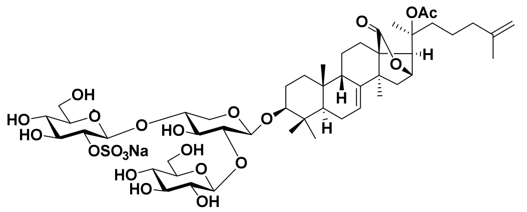

Colochiroside E

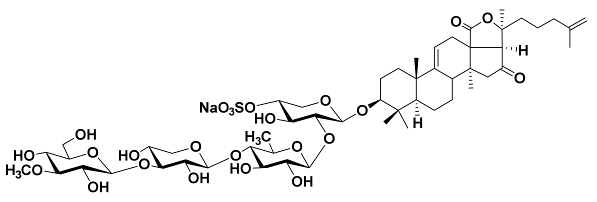

Neothyonidioside

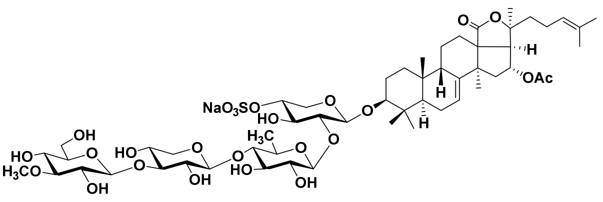

Lefevreoside B

Figure S7. Structures of magnumosides C2–C4, colochirosides A1–A3, B1–B3, C, D, and E, neothyonidioside and lefevreoside B.

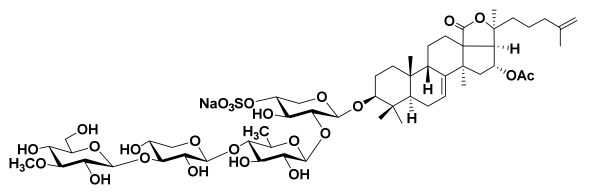

Lefevreoside C

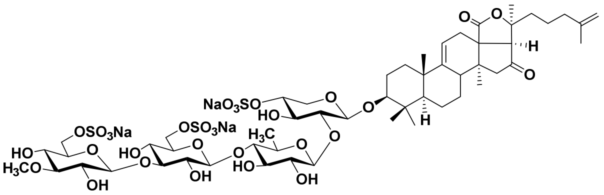

Hemoiedemoside B

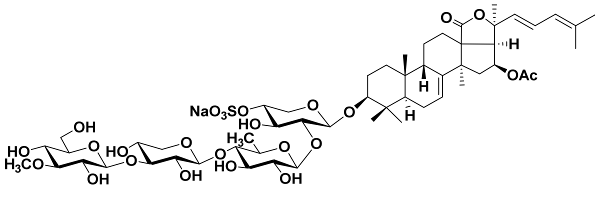

Typicoside A1

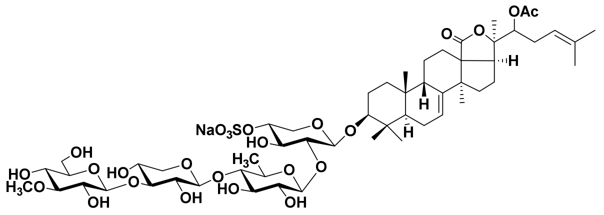

Typicoside A2

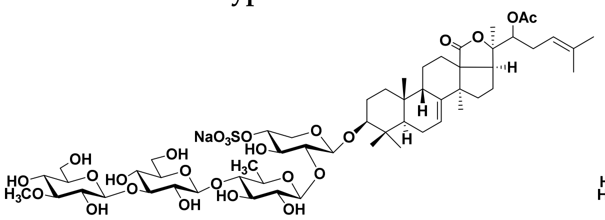

Typicoside B1

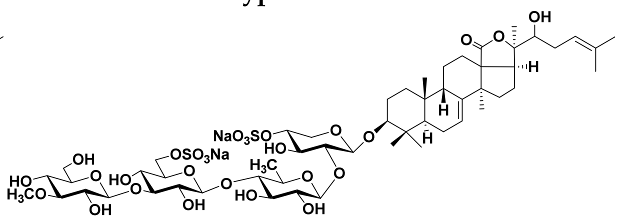

Typicoside C1

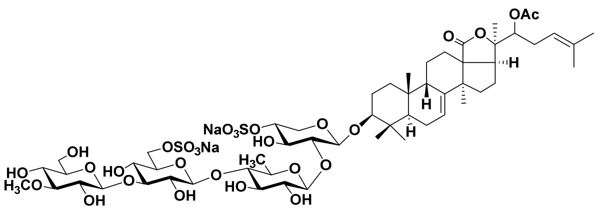

Typicoside C2

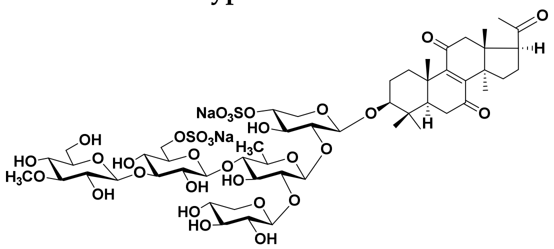

Fallaxoside C1

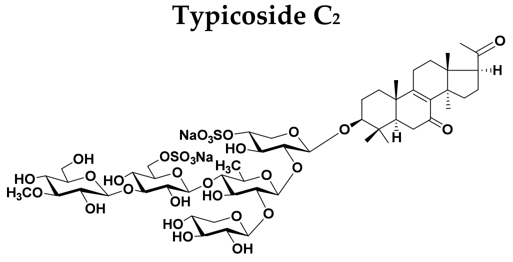

Fallaxoside C2

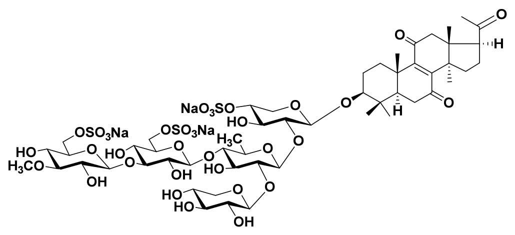

Fallaxoside D1

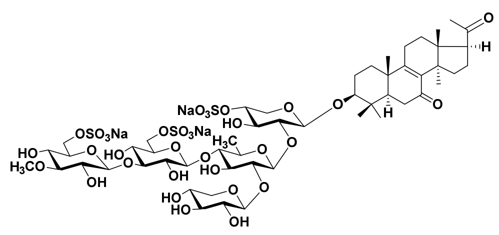

Fallaxoside D2

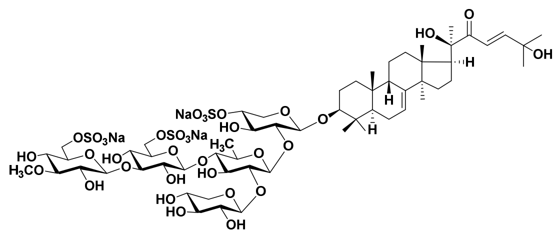

Fallaxoside D6

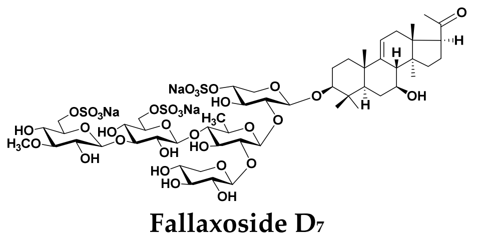

Fallaxoside D7

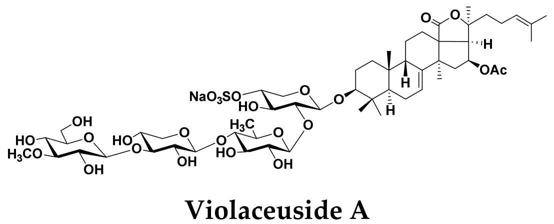

Violaceuside A

Figure S8. Structures of hemoiedemoside B, lefevreoside C, typicosides A1, A2, B1, C1, and C2, fallaxosides C1, C2, D1, D2, D6, and D7 and violaceuside A.

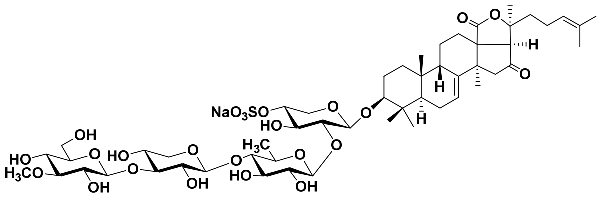

Violaceusoside C

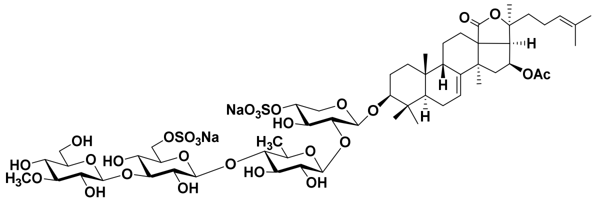

Violaceusoside D

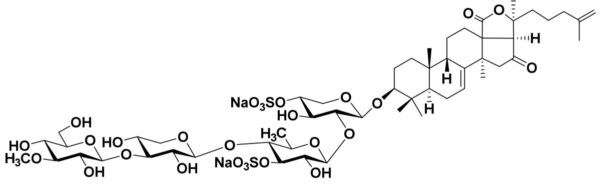

Violaceusoside E

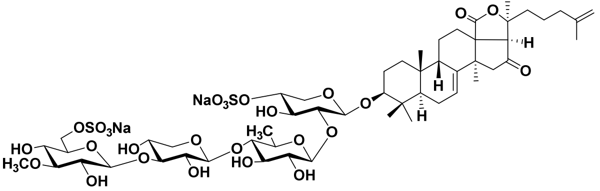

Violaceuside II

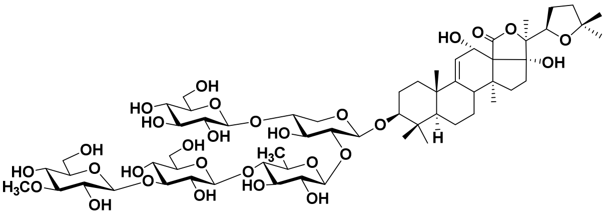

Holothurinoside A

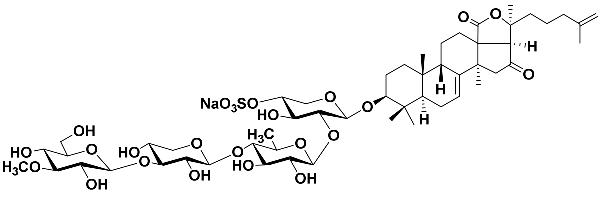

Philinopside E

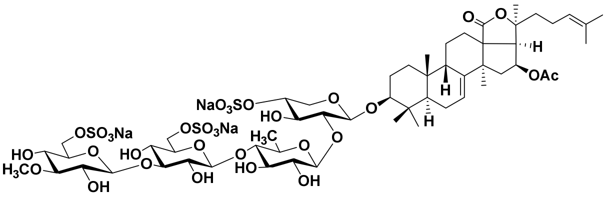

Liouvilloside A

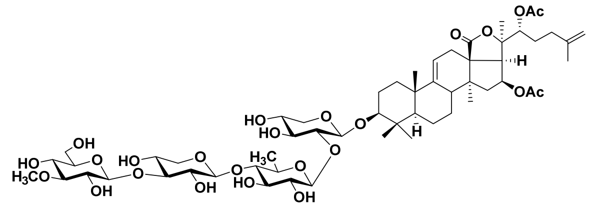

Cladoloside A_2

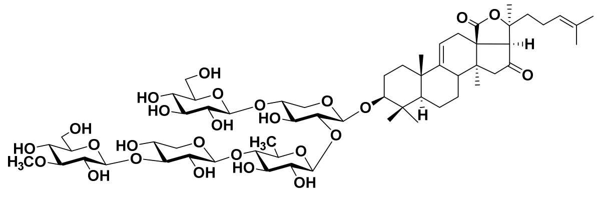

Cladoloside B

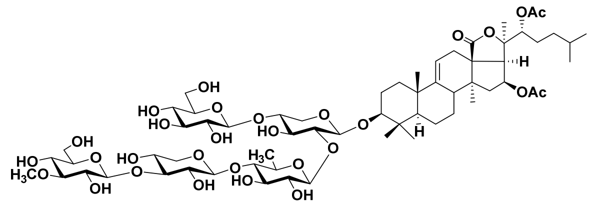

Cladoloside B_1

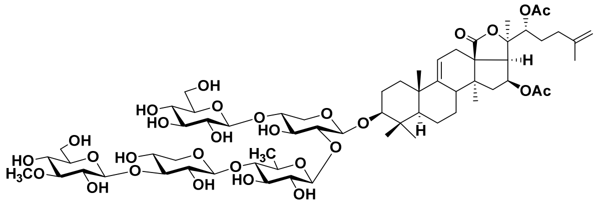

Cladoloside B_2

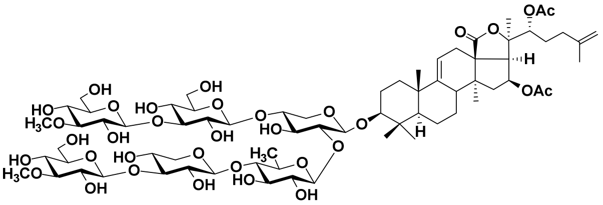

Cladoloside C

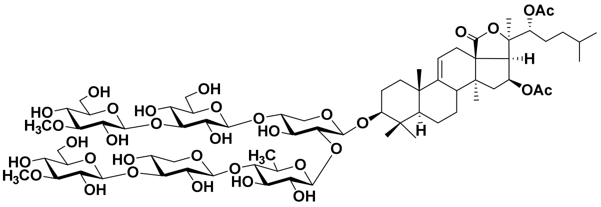

Cladoloside C_1

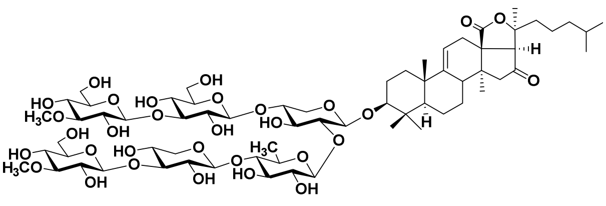

Cladoloside C_2

Figure S9. Structures of violaceusosides C, D, and E, violaceuside II, holothurinoside A, liouvilloside A, philinopside E and cladolosides A_2, B, B_1, B_2, C, C_1, and C_2.

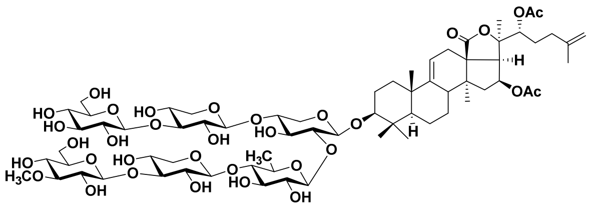

Cladoloside D

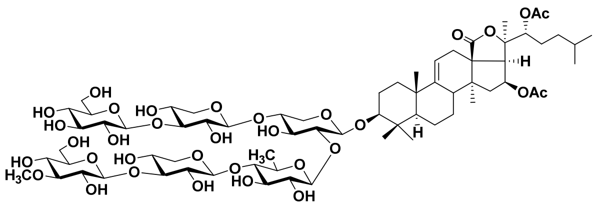

Cladoloside D1

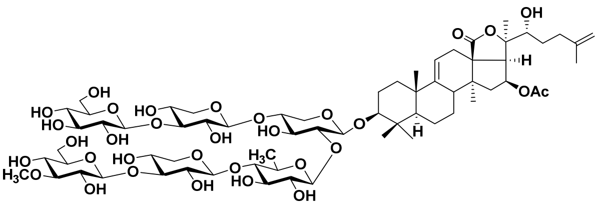

Cladoloside D2

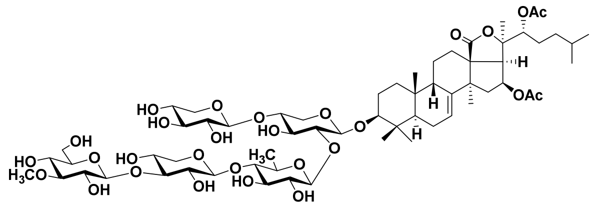

Cladoloside E1

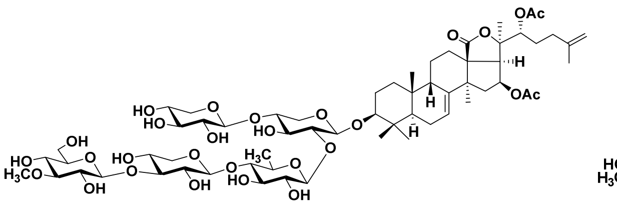

Cladoloside E2

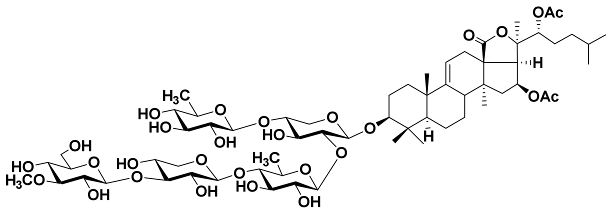

Cladoloside F1

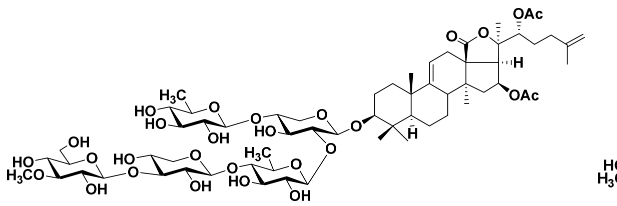

Cladoloside F2

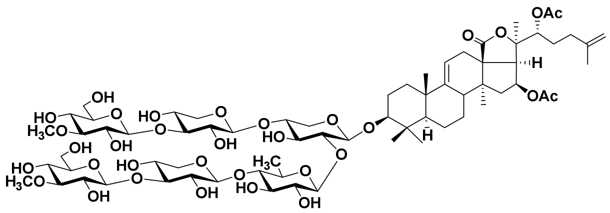

Cladoloside G

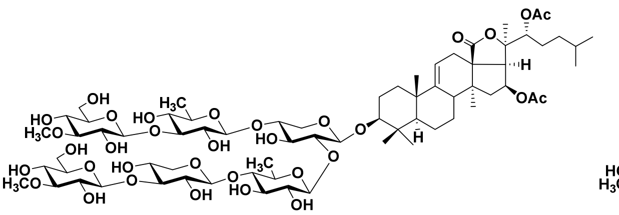

Cladoloside H1

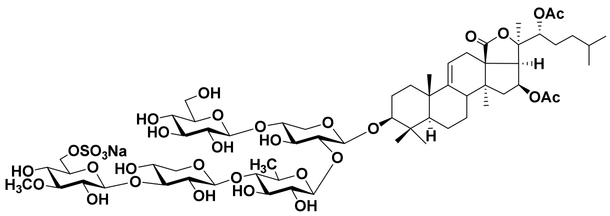

Cladoloside I1

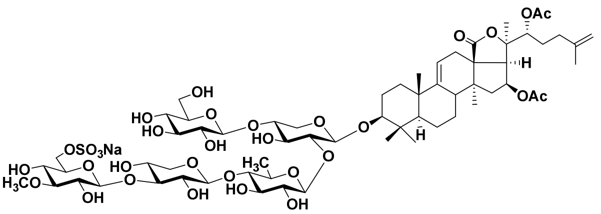

Cladoloside I2

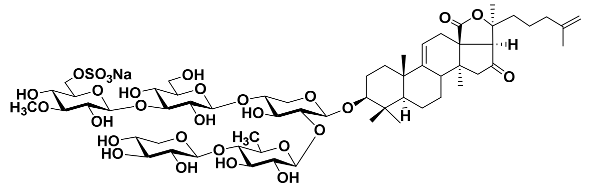

Cladoloside J1

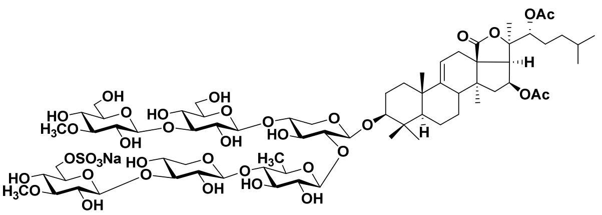

Cladoloside K1

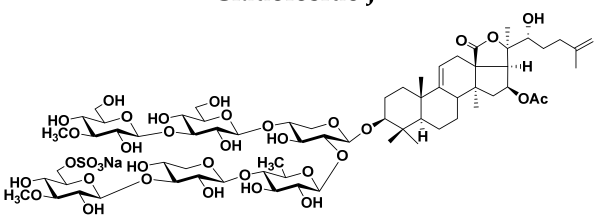

Cladoloside K2

Figure S10. Structures of cladolosides D, D_1, D_2, E_1, E_2, F_1, F_2, G, H_1, I_1, I_2, J_1, K_1, and K_2.

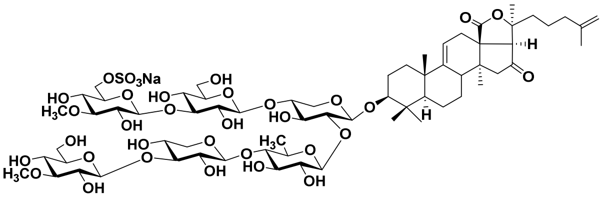

Cladoloside L1

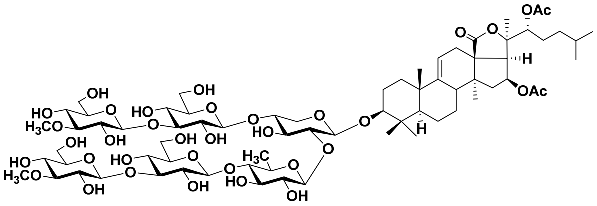

Cladoloside M

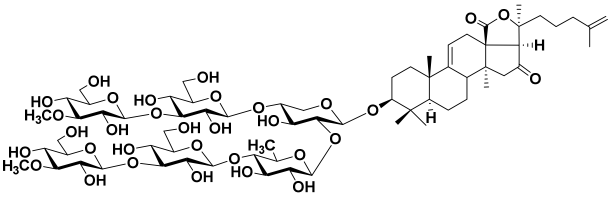

Cladoloside M1

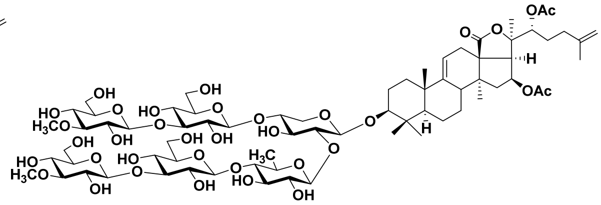

Cladoloside M2

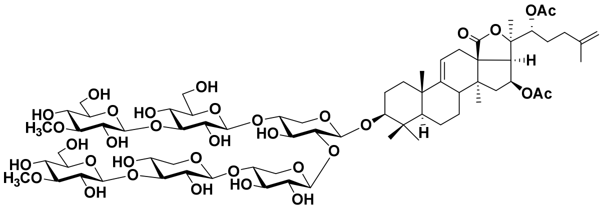

Cladoloside N

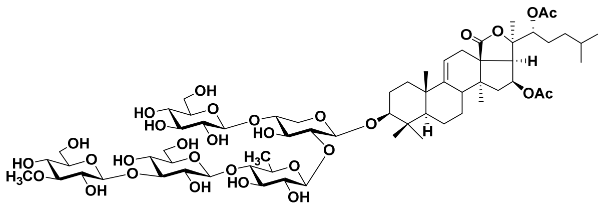

Cladoloside O

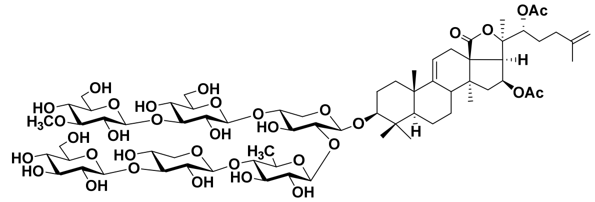

Cladoloside P

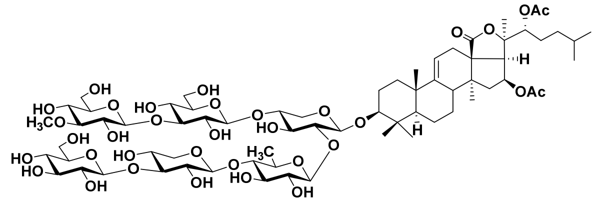

Cladoloside P1

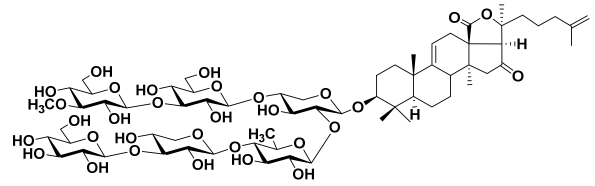

Cladoloside P2

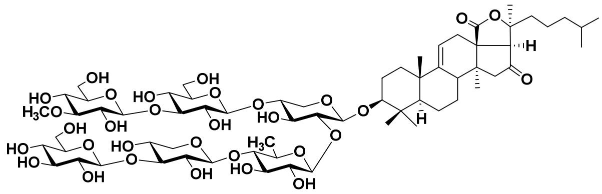

Cladoloside P3

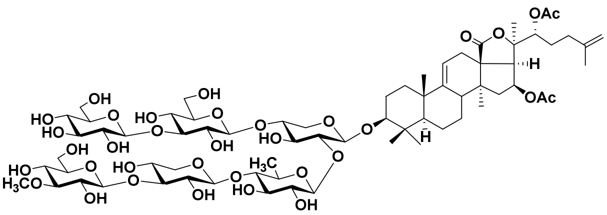

Cladoloside Q

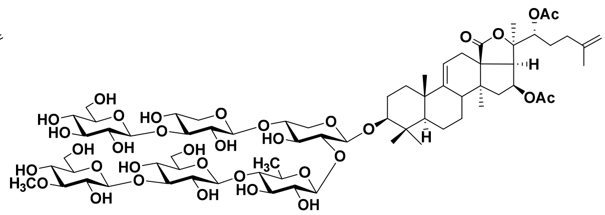

Cladoloside R

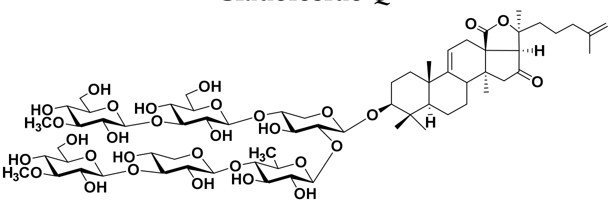

Holotoxin A1

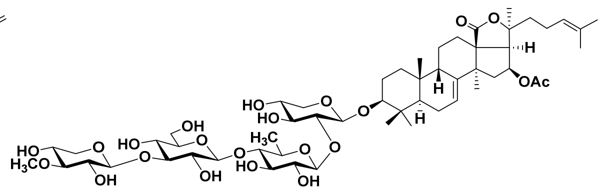

Cucumarioside A1

Figure S11. Structures of cladolosides L1, M, M1, M2, N, O, P, P1–P3, Q, and R, holotoxin A1 and cucumarioside A1.

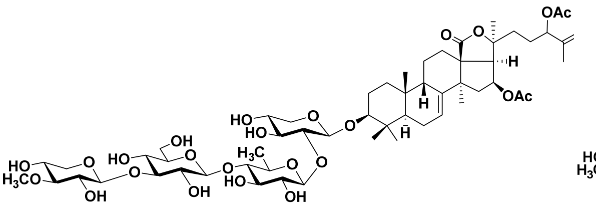

Cucumarioside A_2

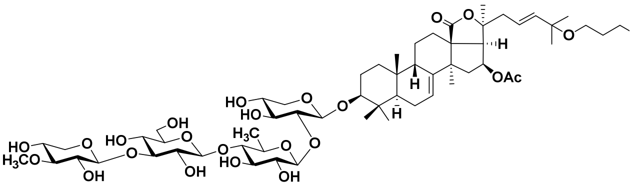

Cucumarioside A_3

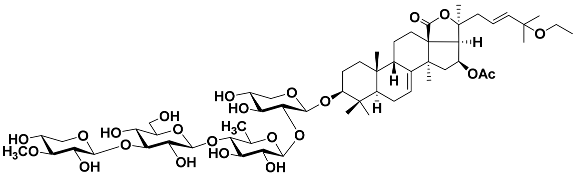

Cucumarioside A_4

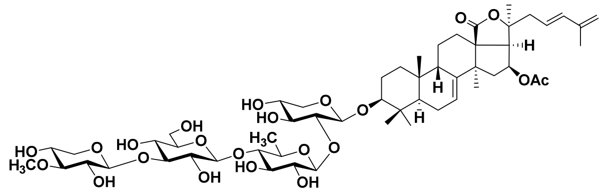

Cucumarioside A_6

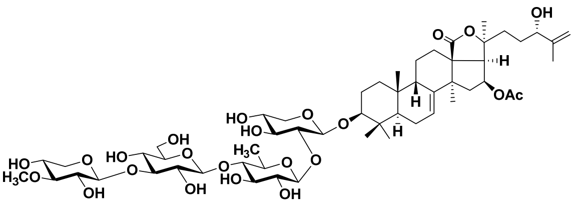

Cucumarioside A_7

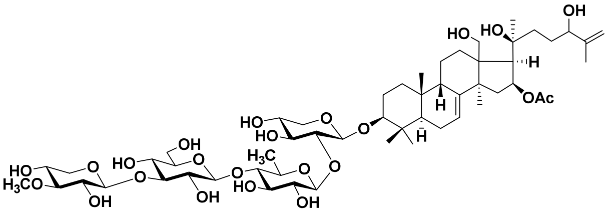

Cucumarioside A_9

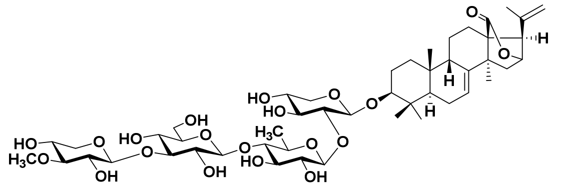

Cucumarioside A_{10}

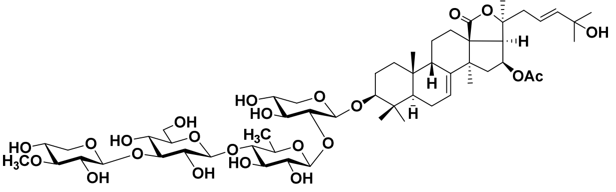

Cucumarioside A_{11}

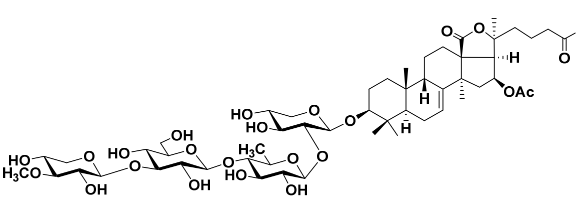

Cucumarioside A_{12}

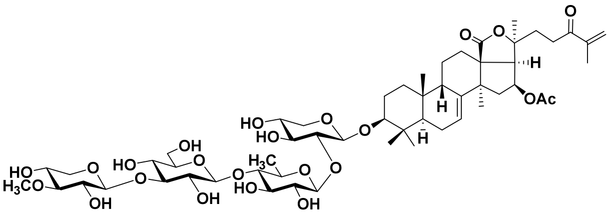

Cucumarioside A_{13}

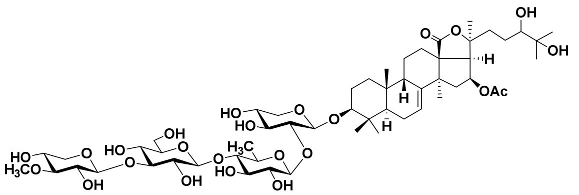

Cucumarioside A_{14}

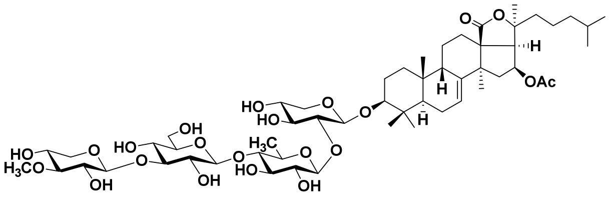

Cucumarioside A_{15}

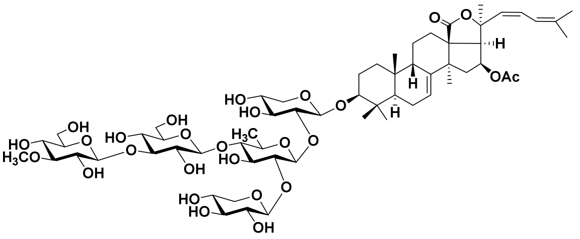

Cucumarioside D

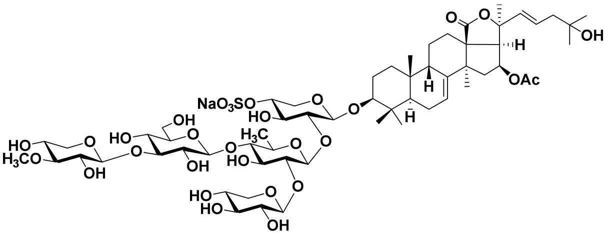

Cucumarioside H_2

Figure S12. Structures of cucumariosides A_2–A_4, A_6, A_7, A_9–A_{15}, D, and H_2.

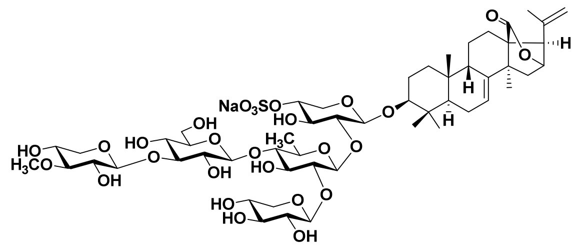

Cucumarioside H_3

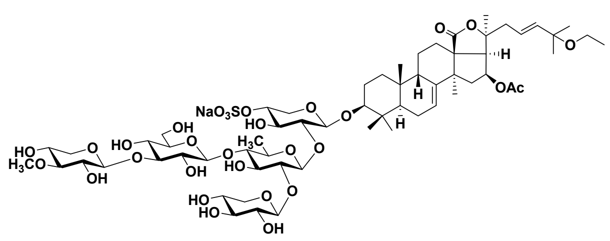

Cucumarioside H_4

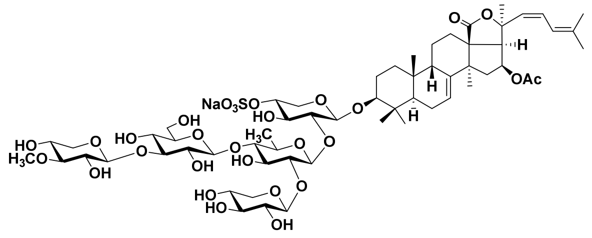

Cucumarioside H_5

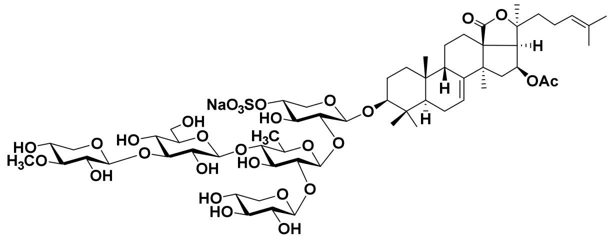

Cucumarioside H_6

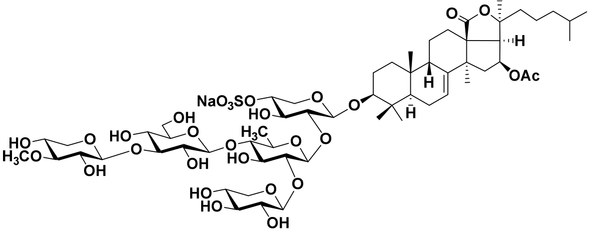

Cucumarioside H_7

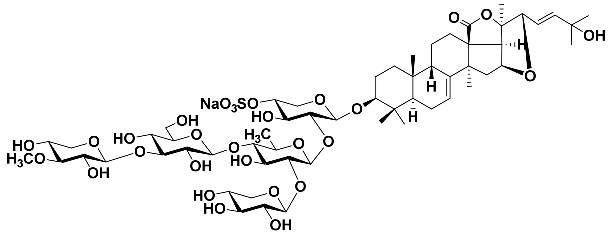

Cucumarioside H_8

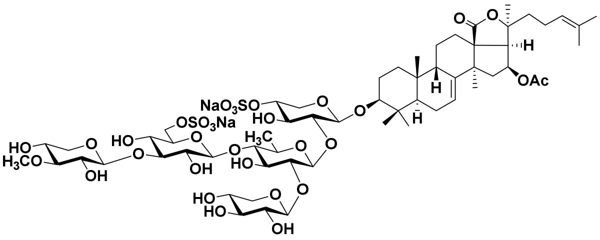

Cucumarioside I_1

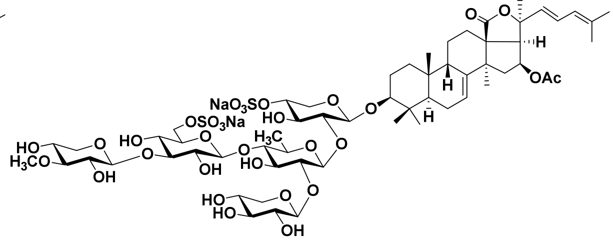

Cucumarioside I_2

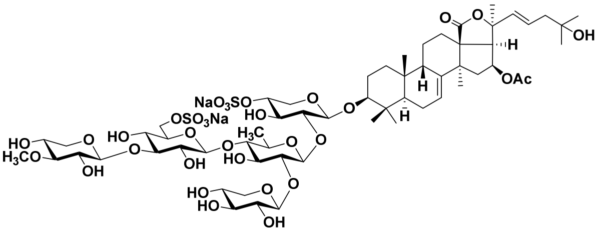

Cucumarioside I_3

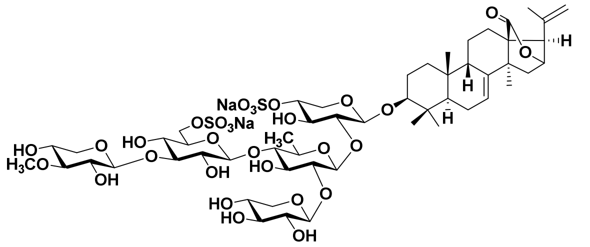

Cucumarioside I_4

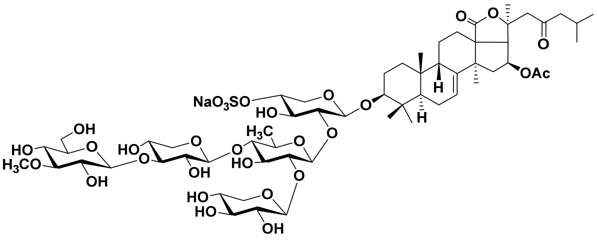

Cucumarioside A_0-1

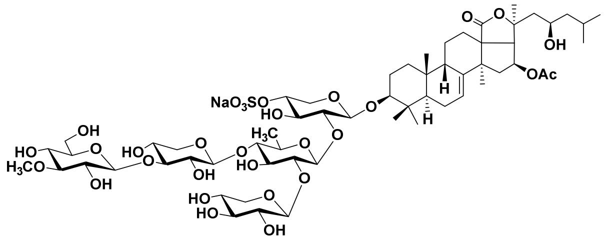

Frondoside D

Figure S13. Structures of cucumariosides H_3–H_8, and I_1–I_4, cucumarioside A_0-1 and frondoside D.

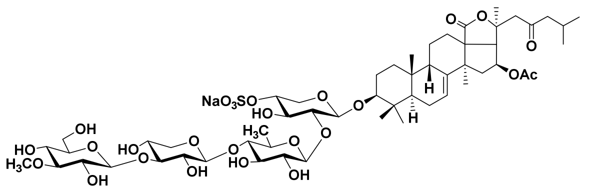

Okhotoside A1-1

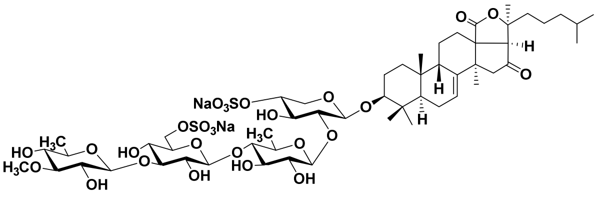

Turquetoside A

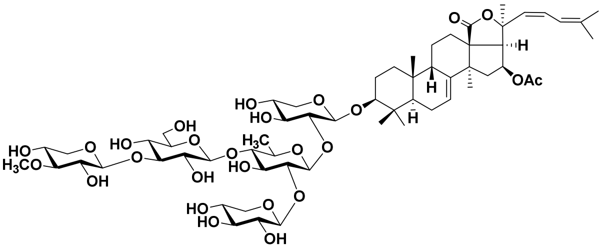

Cucumarioside C1

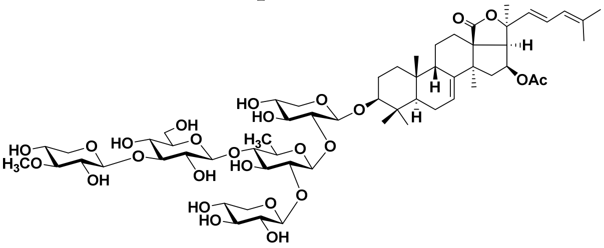

Cucumarioside C2

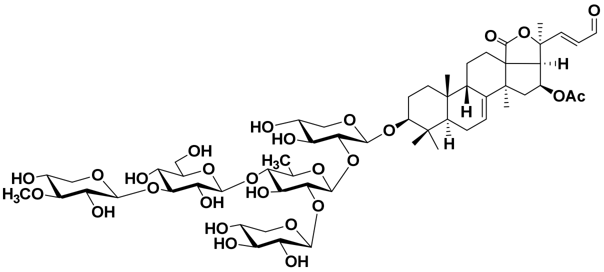

Pacificusoside A

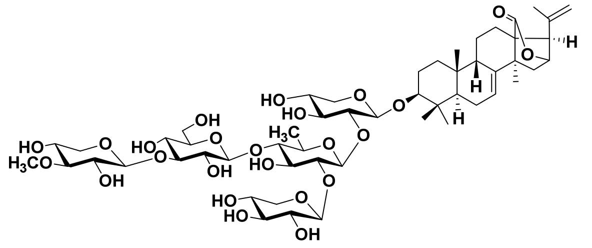

Pacificusoside B

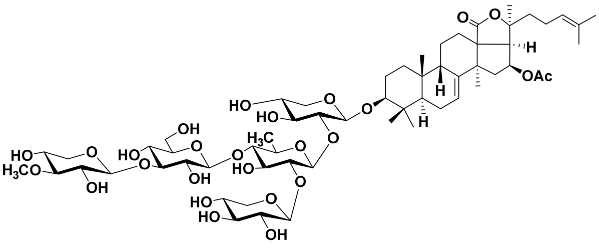

Pacificusoside C

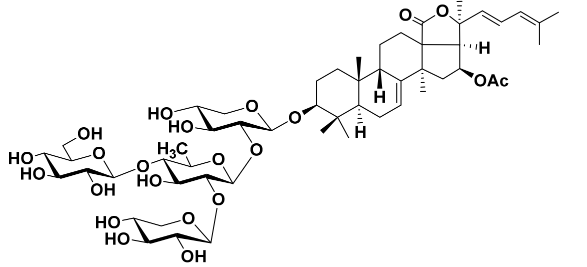

Pacificusoside E

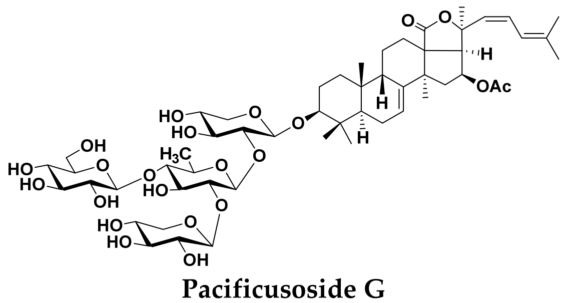

Pacificusoside G

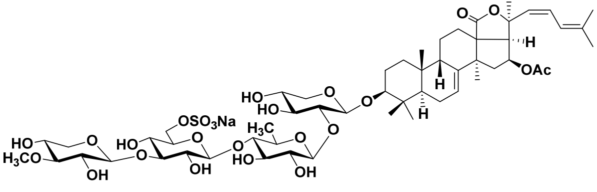

Pacificusoside H

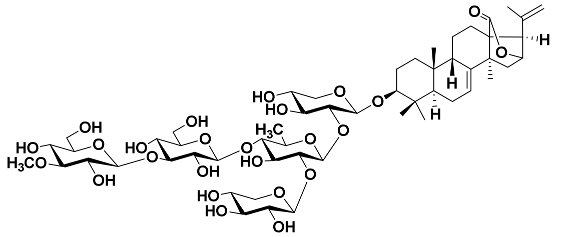

Pacificusoside J

Figure S14. Structures of okhotoside A1-1, turquetoside A, cucumariosides C1 and C2, pacificusosides A, B, C, E, G, H, and J.

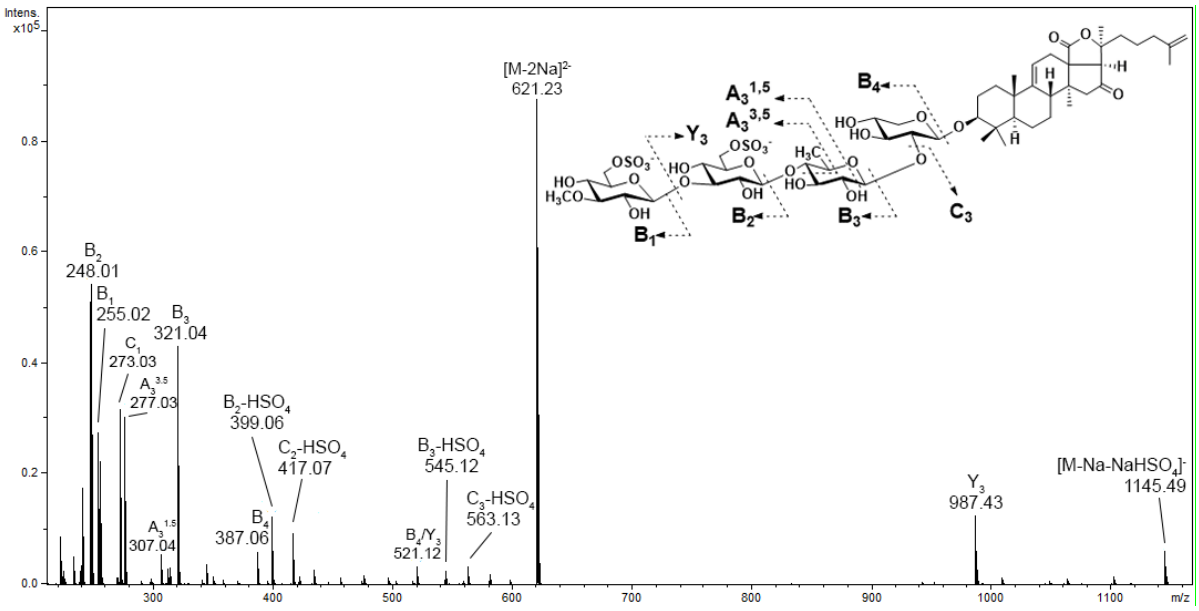

Figure S15. The MS/MS spectrum of $[M{-}2Na]^{2-}$ precursor ion of psolusoside A.

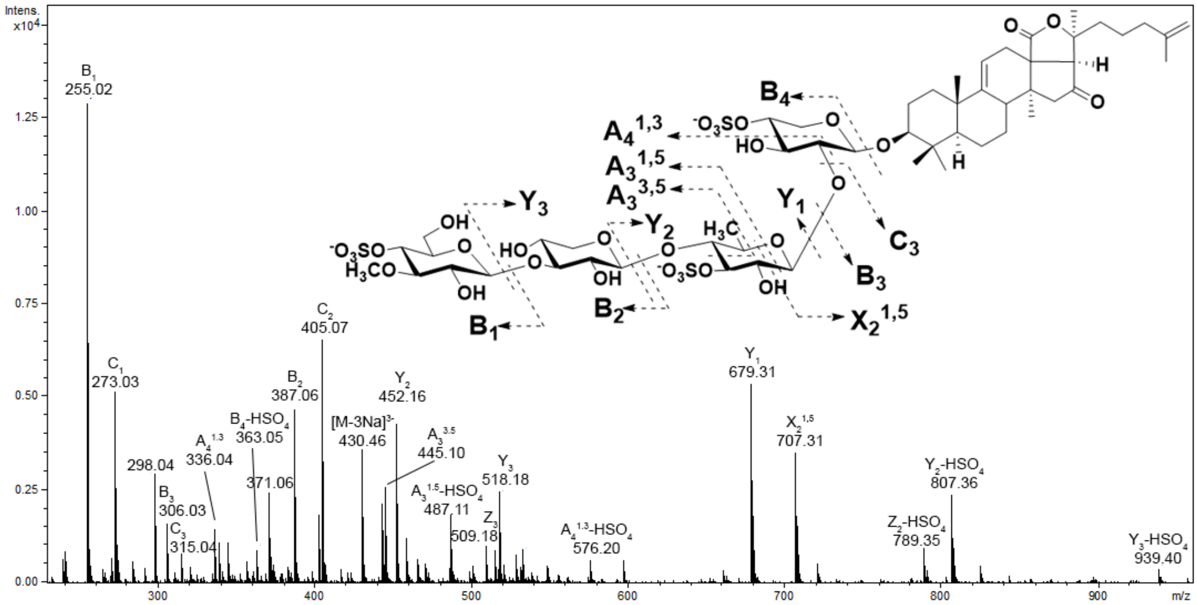

Figure S16. The MS/MS spectrum of $[M{-}3Na]^{3-}$ precursor ion of quadrangularisoside D_2.

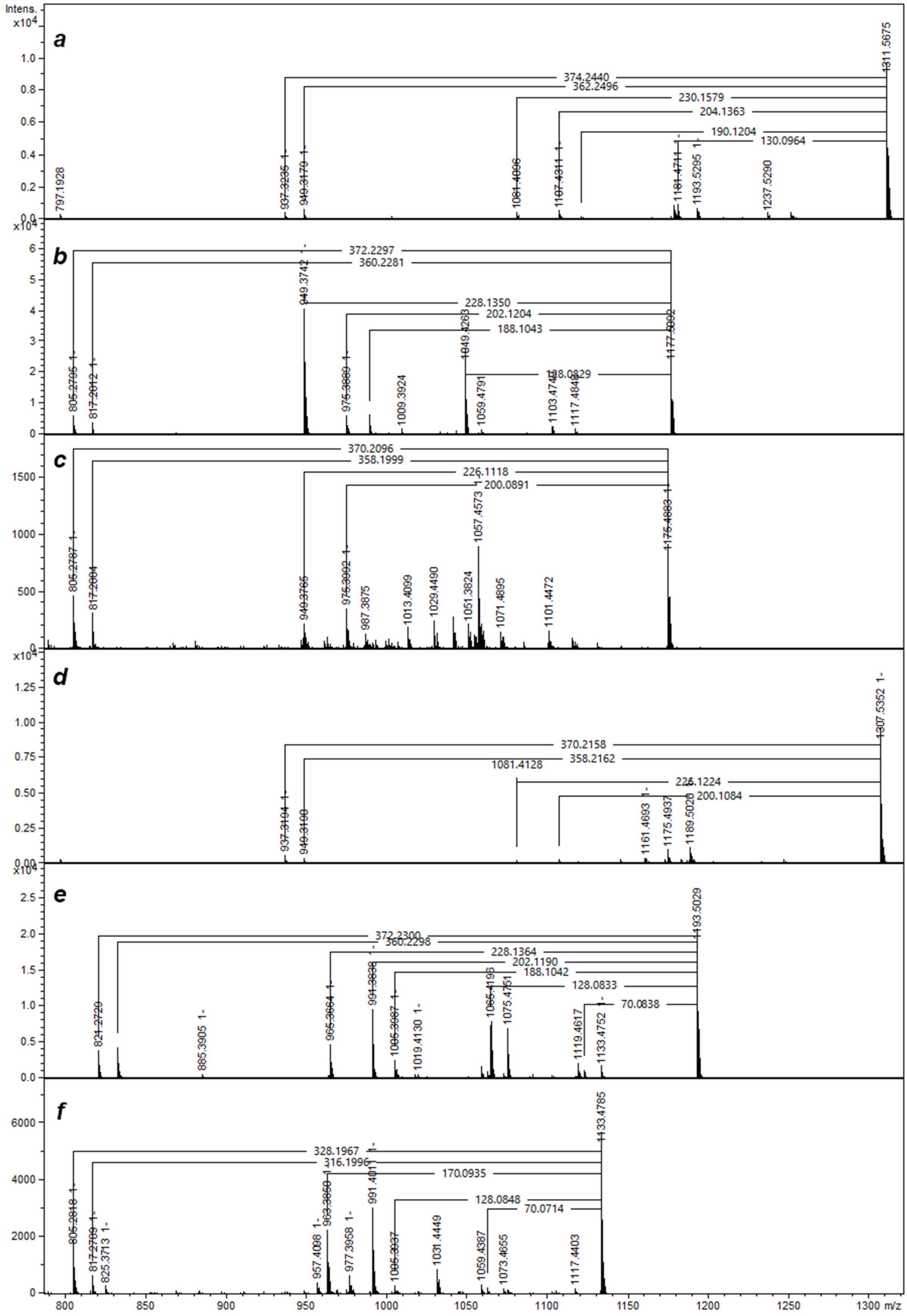

Figure S17. The MS/MS spectra of [M–Na]$^-$ precursor ions of cucumarioside H_7 (a), lefevreoside B (b), typicoside A_1 (c), cucumarioside H_5 (d), colochiroside A_1 (e), and philinopside E (f).

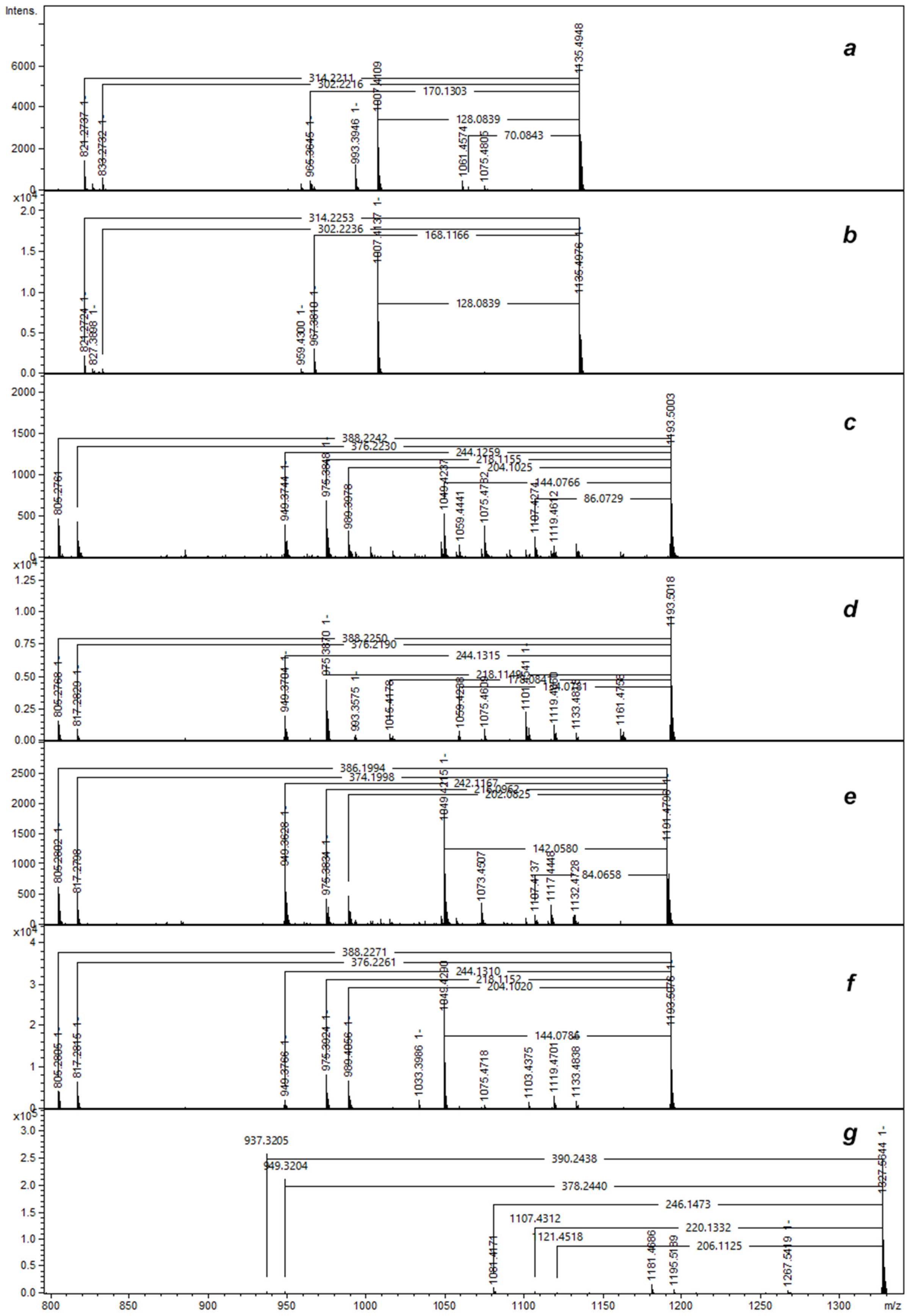

Figure S18. The MS/MS spectra of [M–Na]⁻ precursor ions of colochiroside A2 (a), colochiroside A3 (b), colochiroside B1 (c), colochiroside B2 (d), colochiroside B3 (e), okhotoside A1-1 (f), and frondoside D (g).

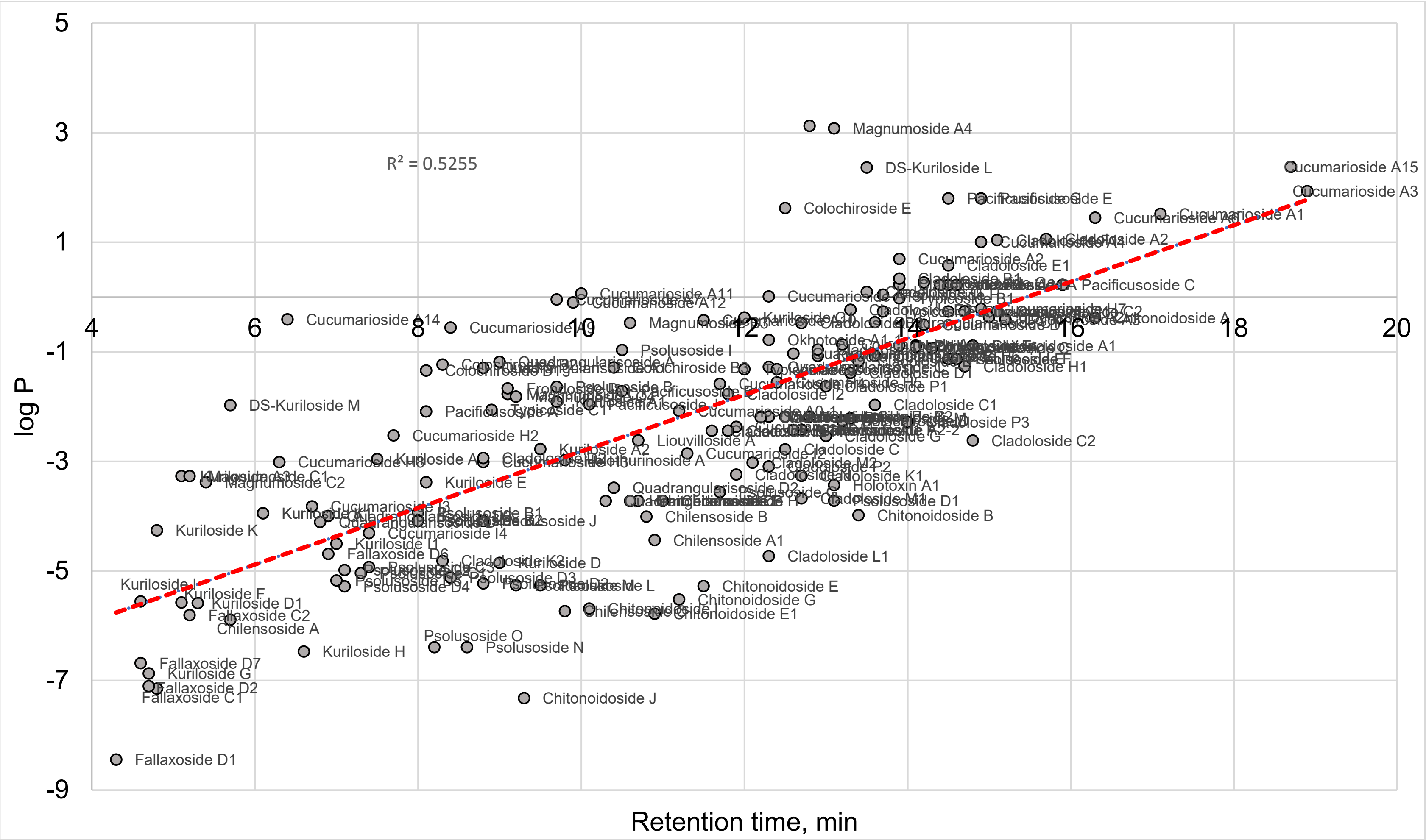

Figure S19. The plot of log P vs. retention time of triterpene glycosides analyzed by LC-MS.

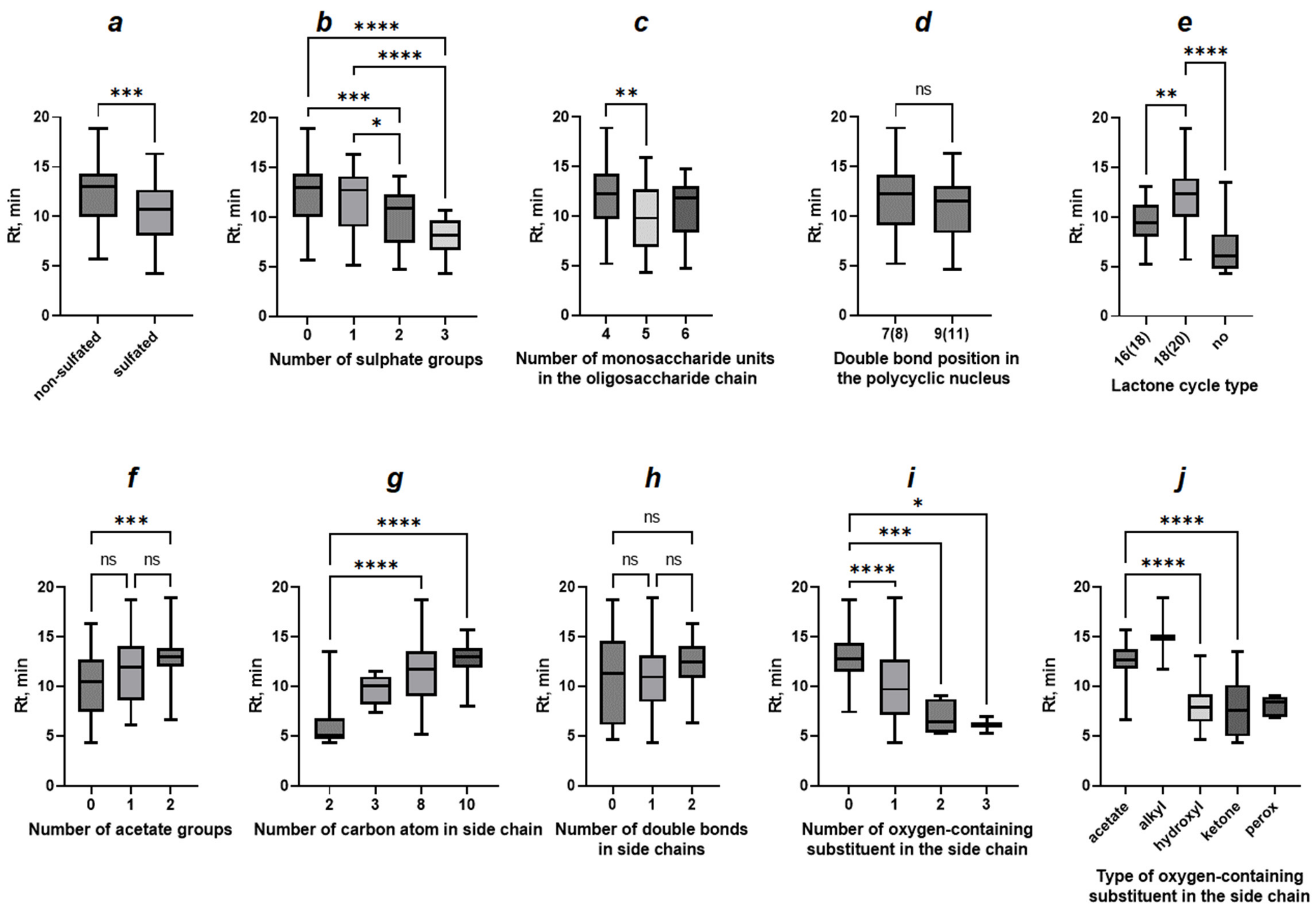

Figure S20. Variations in the retention times of triterpene glycosides related to some structural features: (a) the presence of a sulfate group; (b) the number of sulfate groups; (c) the number of monosaccharide units; (d) the position of the double bond in the polycyclic nucleus of the aglycone; (e) the presence and the type of a lactone cycle; (f) the number of acetoxy groups; (g) the number of carbon atoms in the side chain; (h) the number of double bonds in the side chain; (i) the number of oxygen-containing substituents in the side chain; (j) the type of oxygen-containing substituent in the side chain. Asterisks (* $p < 0.05$, ** $p < 0.01$, *** $p < 0.001$ **** $p < 0.0001$) indicate significant differences between groups.

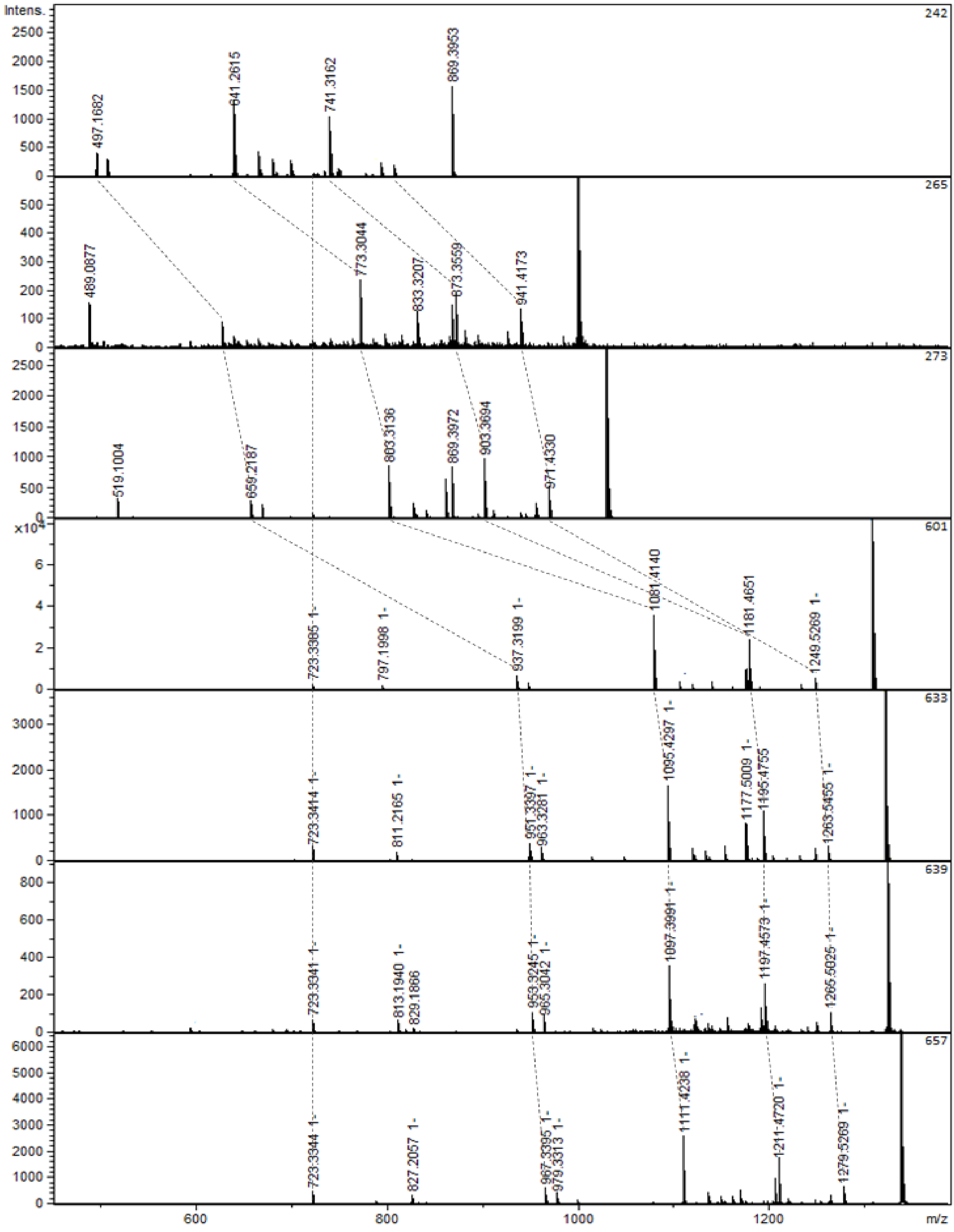

Figure S21. The MS/MS spectra of [M–Na]$^-$ precursor ions of cucumarioside H$_6$ (**601**) and structure-related compounds detected in *E. fraudatrix* extract (**242**, **265**, **273**, **633**, **639**, **357**).
